# Synthesis, SAR Analysis, and Antifungal Potency against Plant Pathogenic Fungi of New Azidopyrazoles

**DOI:** 10.1021/acsomega.6c07653

**Published:** 2026-09-22

**Authors:** Daniel Canseco-González, Rosa E. Sánchez-Fernández, Elizabeth Navarro-Cerón, Stephania Olivares-Sánchez, Alejandro O. Viviano-Posadas, Alejandro Dorazco-González, Ricardo Parra-Unda, Gilmar López-Armenta, Adrián L. Orjuela, Jorge Alí-Torres

**Affiliations:** † SECIHTI-Laboratorio Nacional de Investigación y Servicio Agroalimentario y Forestal, Universidad Autónoma Chapingo, Texcoco de Mora, CP 56230, México; ‡ Laboratorio Nacional de Investigación y Servicio Agroalimentario y Forestal, Universidad Autónoma Chapingo, Texcoco de Mora, CP 56230, México; § Instituto de Química, Universidad Nacional Autónoma de México, Circuito Exterior, Ciudad Universitaria, México CDMX CP 04510, México; ∥ Unidad de Investigaciones en Salud Publica “Dra. Kaethe Willlms”, Facultad de Ciencias Químico-Biológicas, Universidad Autónoma de Sinaloa, Culiacán, Sinaloa CP 80013, México; ⊥ Instituto de Investigaciones Científicas y Servicios de Alta Tecnología (INDICASAT AIP), Panamá City 0801 Panamá; # Departamento de Química, Universidad Nacional de Colombia - Sede Bogotá, Bogota 111321, Colombia

## Abstract

The development of potent synthetic antifungal agents for plants is an ongoing challenge of modern chemistry that impacts biological, medicinal and environmental sciences. Herein, a concise Structure–Activity Relationship (SAR) model involving seven synthetic azidopyrazoles toward three pathogens of agricultural products and ornamental plants: *Fusarium oxysporum*, *Rhizoctonia* sp., and *Alternaria alternata* is described. The set of azidopyrazoles **1–7** includes a central fragment of 3-azido-1*H*-pyrazole substitutes in different positions of the ring (5-substituted, R = H, **1**; R = −CH_3_, **2**; R = –Ph, **3**; R = –PhOCH_3_, **5**; 4-substituted, R = –Ph, **4**; R = –(4-Cl-Ph), **6**) and 3-substituted 5-azido-1*H*-pyrazole (R = thiophene, **7**) which are prepared via a high-yielding (>85%), one-step synthesis from commercial cheap aminopyrazoles. Compounds **3** and **7** showed the greatest inhibition with IC_50_ values of 26.52 ± 1.14 and 26.75 ± 1.36 μg/mL for *F. oxysporum*; 18.63 ± 1.08 and 14.39 ± 1.09 μg/mL for *Rhizoctonia* sp.; and 15.53 ± 1.14 and 21.02 ± 1.37 μg/mL for *A. alternata*. SAR analysis revealed that aryl substitution at C5 increased markedly the antifungal potency. In contrast, unsubstituted and alkyl-substituted pyrazoles (**1**, **2**) had low activity. Combining **3** with Ag-nanoparticles (NPs) doubled its efficacy only against *Rhizoctonia* sp. (AgNPs, 10 nm, 100 ng/mL). From IC_50_ = 18.63 ± 1.08 to 9.65 ± 1.38 μg/mL). Molecular docking with MEP36 supported these results: compound **3** had the highest binding affinity (−6.8 kcal/mol) due to π–alkyl interaction with Val274 residue. Furthermore, root mean square deviation analyses confirmed the tight binding mode.

## Graphical abstract

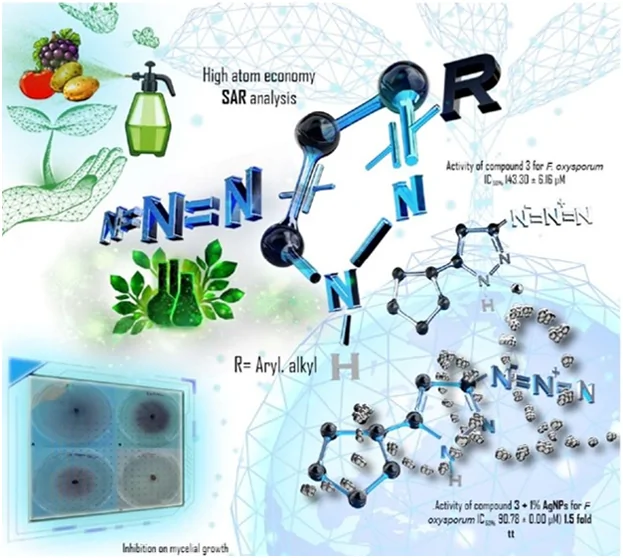

## Introduction

The chemistry of organic azides has been extensively investigated since the 19th century. The first documented synthesis of an azido-substituted organic compound occurred in 1864, when Griess synthesized phenyl azide^1^ (Figure 1A). Organic azides are versatile due to their chemical nature. They consist of three nitrogen atoms arranged linearly, forming a polyatomic anion characterized by its high reactivity^2^ (Figure 1B). For example, reactions of organic azides with alkynes yield 1,4–1,2,3-triazoles via Huisgen 1,3-dipolar cycloadditions^3^ or imines can be formed when reacting with carbonyl compounds (Aza-Wittig reaction).^4^

**Figure 1:**
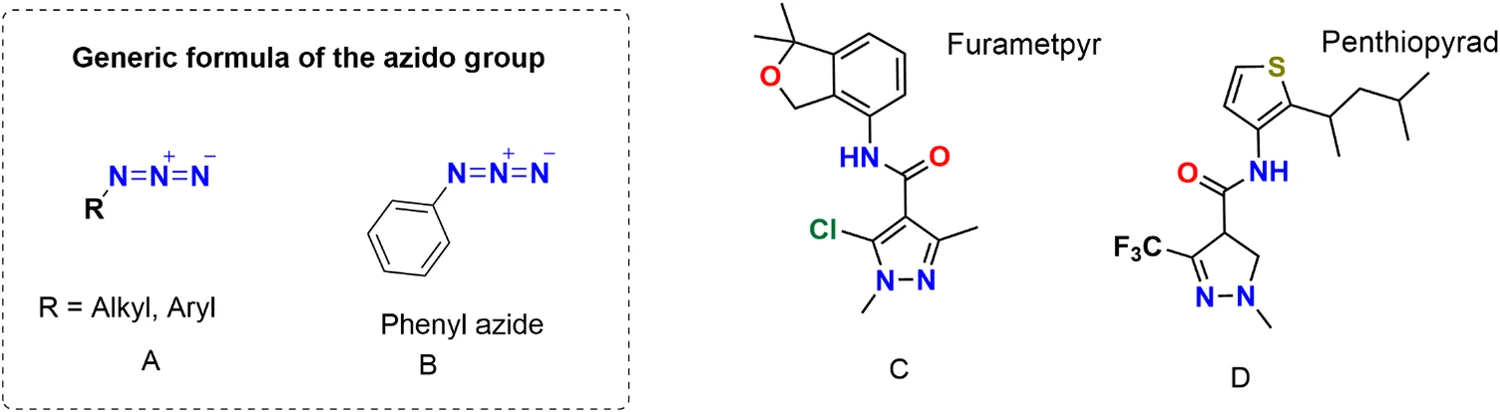
(A–B) Generic formula of the azido group and phenyl azide. (C–D) Commercial fungicides based on pyrazole frameworks.

Azido-substituted organic compounds have applications in various fields, including the explosive-related industry (i.e., sodium azide NaN_3_)^5^ and medicinal chemistry.^6^ Although NaN_3_ is applied as a pest control agent, its high toxicity and phytotoxicity limit its desirability for use.^7,8^ On the other hand, pyrazoles are five-membered heterocyclic compounds with two adjacent nitrogen atoms.^9,10^ These molecules are found in biology, medicine, material chemistry, and agriculture such as fungicides^6^ or agrochemicals, but their synthesis typically involves multiple steps.^11^

Consequently, atom economy^12^ emerges as a pivotal factor in contemporary chemical synthesis, underscoring the imperative for enhancing process sustainability. Atom economy, a term coined in the field of synthetic chemistry, signifies the principle of leveraging novel synthetic methodologies to optimize the integration of starting materials into the final products. Recently, functionalized pyrazole derivatives have been evaluated as pesticides due to properties such as low toxicity and activity.^13^ Recently, pyrazole derivatives have attracted attention for their applications as herbicides,^14^ fungicides,^15–17^ and insecticides.^14,18–20^ For instance, furametpyr^21^ and penthiopyrad^21^ (Figure 1C,D), both fungicides employed in various crops for pest and disease management, have noted advantages and disadvantages.

Furametpyr is an aromatic pyrazole amide which is used to manage rice sheath rust by inhibiting the biosynthesis of aspartate and glutamate in the target fungus. Penthiopyrad is a broad-spectrum fungicide, demonstrates efficacy against diseases resistant to strobilurins and demethylation inhibitors, yet has documented serious mammalian toxicity, including effects on liver, thyroid, adrenal, and kidney glands, which may increase cancer risk.^22^

Despite the established applications of azido-substituted organic molecules in several fields, their use as antifungal compounds against plant pathogenic fungi is practically unexplored. This work presents a concise yet comprehensive rationalized synthesis of seven novel aryl and nonaryl azido-pyrazoles, exhibiting high atom economy in a single-step synthesis. Furthermore, it encompasses a comprehensive examination of their structure–activity relationship (SAR) against three fungal plant pathogens. This SAR is supported by in-silico calculations, such as molecular docking and molecular dynamic simulations.

## Experimental Section

### Chemicals and Materials

Amino pyrazole precursors were purchased from Aldrich and used directly without further purification. NaNO_2_ and sodium azide (NaN_3_) salts were also purchased from commercial distributors. All solvents used for the syntheses of azido pyrazole derivatives were used as received from Aldrich. In most cases HPLC quality solvents were used as water or ethyl acetate used for extraction procedures. Unless specified otherwise, NMR spectra were recorded at 25 °C on a Bruker Advance spectrometer operating at 300 (^1^H NMR) and 75 MHz (^13^C NMR), respectively. The chemical shifts (δ) are given in ppm relative to TMS as internal standard. Assignments are based on homo- and heteronuclear shift correlation spectroscopy. Low resolution mass spectrometry experiments were recorded on a Shimadzu GCMSQP2010 Plus in the EI mode, 70 eV, 200 °C via direct inlet probe at positive polarity.

### General Procedure to Synthesize Azidopyrazoles **1**–**7**

The new azido pyrazole derivatives **1–7** were successfully synthesized using a modified established procedure,^23^ starting from primary amine pyrazoles via the formation of the diazonium salt group, which was subsequently replaced by the azido group using NaN_3_. Scheme 1 shows the general reaction and chemical structures of azido pyrazoles **1–7**.

**Scheme 1:**
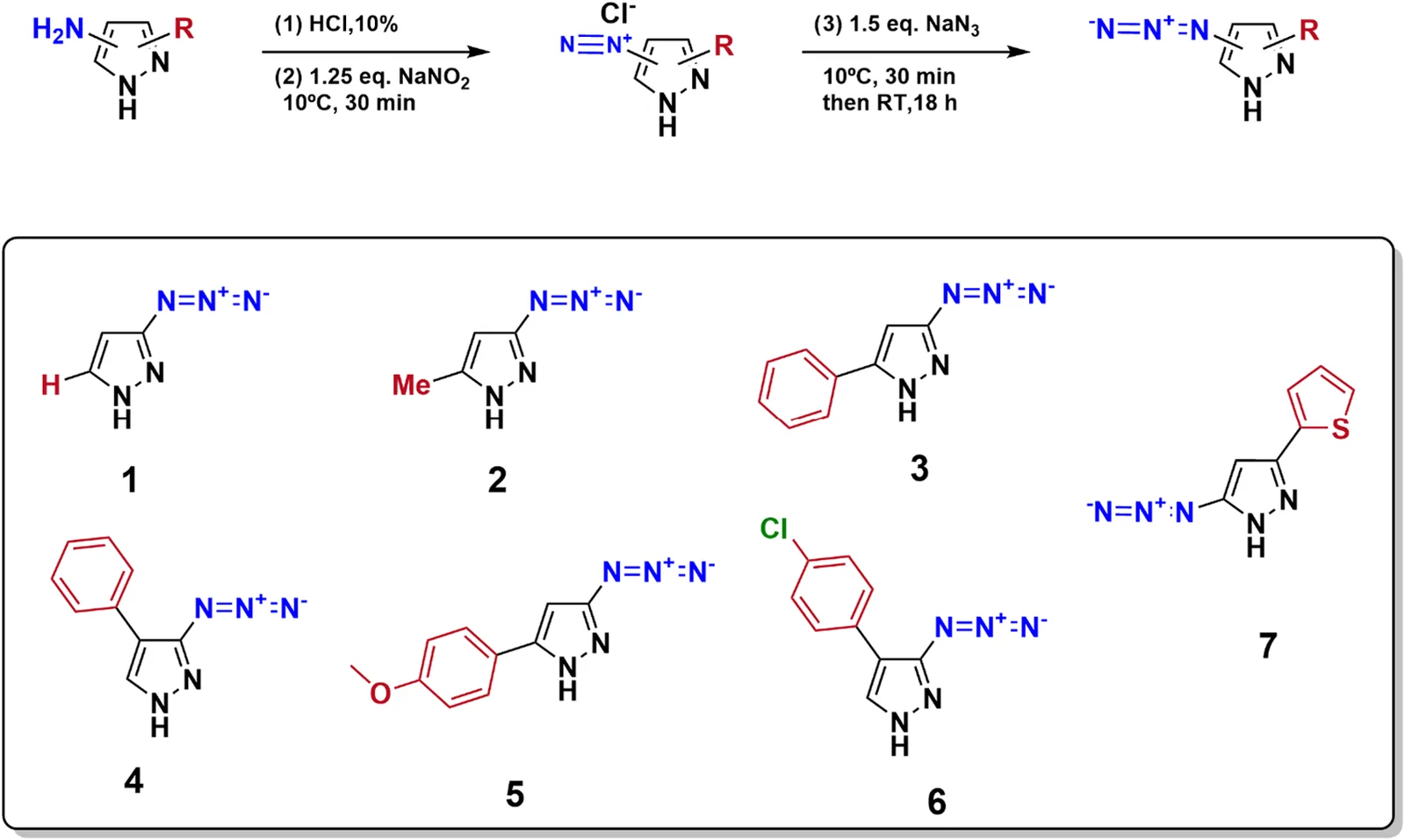
Synthetic Path and Chemical Structure of the Azido Pyrazoles **1–7**

### Chemical Synthesis

One equiv of the corresponding amino pyrazole was placed in a round-bottomed flask and dissolved in a 10% water/hydrochloric acid solution. A homogeneous solution was observed, indicating complete dissolution of the starting material.

The solution was cooled to 10 °C using a water/ice bath and stirred for 10 min. Subsequently, 1.25 equiv of NaNO_2_ were slowly added to the ammonium salt formed, and the mixture was stirred for 30 min at 10 °C. Next, 1.50 equiv of NaN_3_ were added and the reaction continued at 10 °C for an additional 30 min. The final solution was stirred for another 18 h at room temperature.

Note: For compound **7** the reaction was left stirred for 6 h only, otherwise the formation of a polymeric compound is obtained, and the yield of the desirable compound is affected.

### Isolation

For all compounds, the crude products were isolated by liquid–liquid extraction using ethyl acetate/water.

Prior to extraction, the crude product solution was neutralized with NaHCO_3_ until the pH reached neutral. The extraction procedure involved three rounds of extraction with ethyl acetate, followed by washing with water, brine, and drying with anhydrous Na_2_SO_4_. The mixed organic phases containing ethyl acetate and the crude product were evaporated using a rotary evaporator until the desired residue was obtained. The yielded products are color powders (reddish, greenish, orange or yellow).

### Purification

The purification process involved reprecipitation using a CHCl_3_/pentane system. A solution of the crude product was formed with minimal CHCl_3_, which was then added to cold pentane cooled to −15 °C. This method yielded pure products that were dried under vacuum until no solvents were detected by spectroscopic analyses.

### Chemical Synthesis of 3-Azido-1*H*-pyrazole, **1**

Following the general procedure to obtain the desirable compound, 500 mg, 6.02 mmol of 1*H*-pyrazol-3-amine was reacted with NaNO_2_ (520 mg, 7.525 mmol) and subsequently with NaN_3_ (587 mg, 9.030 mmol) after, isolation and purification a cotton-like whitish solid corresponding to pure azido-pyrazole **1** was yielded. (578 mg, 88%). ^1^H NMR (CDCl_3_, 300 MHz): δ 12.37­(s, 1H, H_pyrazole, broad_), 7.56 (d, *J* = 2.4 Hz, 1H_pyrazolel_), 6.01 (d, *J* = 2.4 Hz, 1H_pyrazolel_). ^13^C­{1H} NRM (CDCl_3_, 75 MHz): δ 148.36 (C_quaternary_), 131.03 (CH_pyrazole_), 95.97 (CH_pyrazole_). MW (C_3_H_3_N_5_ 109.089 g/mol). HRMS-EI­(+), (C_3_H_3_N_5_ 109.089 g/mol); [M + H]^+^ = calculated 110.04670 *m*/*z*, [M + H]^+^ = found 110.0466 *m*/*z* (78%). IR (cm^–1^): 1185.36 (N_3_ confirmation band),1626.75, 1744.15 (CH_Ar‑pyrazole_), 2111.19 (N_3_ principal intense band), 3127.59 (CH_Ar‑pyrazole_).

### Chemical Synthesis of 3-Azido-5-methyl-1*H*-pyrazole, **2**

Following the general procedure to obtain the desirable compound, 600 mg, 6.18 mmol of 5-methyl-1*H*-pyrazol-3-amine was reacted with NaNO_2_ (603 mg, 7.73 mmol) and subsequently with NaN_3_ (603 mg, 9.27 mmol), after isolation and purification a whitish solid corresponding to pure azido-pyrazole **2** was yielded. (711 mg, 93%). ^1^H NMR (CDCl_3_, 300 MHz): δ 11.85 (s, 1H, H_pyrazole, broad_), 5.76 (s, 1H, H_pyrazole_), 2.32 (s, 3H, alkyl). ^13^C­{1H} NRM (CDCl_3_, 75 MHz): δ 148.51 (C_quaternary_), 141.98 (CH_pyrazole_), 94.96 (CH _pyrazole_), 11.28 (CH_3_). MW (C_4_H_5_N_5_ 123.054 g/mol). HRMS-DART­(+), (C_4_H_5_N_5_ 123.054 g/mol); [M + H]^+^ = calculated 124.06232 *m*/*z*, [M + H]^+^ = found 124.06192 *m*/*z* (100%). IR­(cm^–1^): 1248.80­(N_3_ confirmation band),1653.65 (C=NH), 2116.81 (N_3_ principal intense band), 2877.29, 2983.81 (CH_3_), 3129.37 (CH_Ar‑pyrazole_), 3364.54 (C=NH)

### Chemical Synthesis of 3-Azido-5-phenyl-1*H*-pyrazole, **3**

Following the general procedure to obtain the desirable compound, 500 mg, 3.14 mmol of 5-phenyl-1*H*-pyrazol-3-amine was reacted with NaNO_2_ (271 mg, 3.92 mmol) and subsequently with NaN_3_ (306 mg, 4.71 mmol), after isolation and purification an orangish solid corresponding to pure azido-pyrazole **3** was yielded. (547 mg, 94%). ^1^H NMR (CDCl_3_, 300 MHz): δ 11.48 (s, 1H, H_pyrazole, broad_), 7.58–7.55 (m, 2H_Ar_, Ph_,_), 7.48–7.39 (m, 3H_Ar_, Ph_,_) 6.13 (s, 1H, H_pyrazole_). ^13^C­{^1^H}­NRM (CDCl_3_, 75 MHz): δ 149.35 (C_quaternary_), 145.49 (CH_pyrazole_), 129.92–128.72 (CH_Ar,_ Ph, overlapping signals), 128.79 (C_quaternary,_ Ph), 125.52 (CH_Ar,_ Ph) 93.38 (CH _pyrazole_). MW (C_9_H_7_N_5_ 185.185 g/mol). HRMS-DART­(+), (C_9_H_7_N_5_ 185.185 g/mol); [M + H]^+^ = calculated 186.07797 *m*/*z*, [M + H]^+^ = found 186.07711 *m*/*z* (100%). IR­(cm^–1^): 1001.13, 1067.65, 1143.49 (H_Ar_-Ph),1244.46 (N_3_ confirmation band), 1465.81 (Ph),1653.25 (C=NH), 2118.38 (N_3_ principal intense band), 3130.72 (CH_Ar‑pyrazole_).

### Chemical Synthesis of 3-Azido-4-phenyl-1*H*-pyrazole, **4**

Following the general procedure to obtain the desirable compound, 500 mg, 3.14 mmol of 4-phenyl-1*H*-pyrazol-3-amine was reacted with NaNO_2_ (271 mg, 3.92 mmol) and subsequently with NaN_3_ (306 mg, 4.71 mmol) after, isolation and purification a yellowish solid corresponding to pure azido-pyrazole **4** was yielded. (523 mg, 90%). ^1^H NMR (CDCl_3_, 300 MHz): δ 10.07 (s, 1H, H_pyrazole, broad_), 7.71 (s, 1H, H_pyrazole_) 7.69–7.24 (m, 5H_Ar_, Ph_,_). ^13^C­{^1^H} NRM (CDCl_3_, 75 MHz): δ 145.66 (CH_pyrazole_), 130.77 (CH_Ar,_ Ph), 128.83 (C_quaternary,_ Ph), 127.98 (CH_Ar,_ Ph), 126.95 (CH_Ar,_ Ph), 126.80 (CH_Ar,_ Ph), 112.75 (C_quaternary‑pyrazole_). MW (C_9_H_7_N_5_ 185.185 g/mol), HRMS-DART­(+), (C_9_H_7_N_5_ 185.185 g/mol); [M]^+^ = calculated 185.07010 *m*/*z*, [M]^+^ = found 185.0707 *m*/*z* (58%). IR­(cm^–1^): 1004.63, 1067.18, 1090.36 (H_Ar_-Ph),1251.46 (N_3_ confirmation band), 1467.35 (Ph),1601.52 (C=NH), 2119.20 (N_3_ principal intense band), 2945.45 (CH_Ar_), 3156.04 (CH_Ar‑pyrazole_).

### Chemical Synthesis of 3-Azido-5-(4-methoxyphenyl)-1*H*-pyrazole, **5**

Following the general procedure to obtain the desirable compound, 500 mg, 2.64 mmol of 5-(4-methoxyphenyl)-1*H*-pyrazol-3-amine was reacted with NaNO_2_ (228 mg, 3.30 mmol) and subsequently with NaN_3_ (258 mg, 3.96 mmol), after isolation and purification a yellowish solid corresponding to pure azido-pyrazole **5** was yielded. (510 mg, 90%). ^1^H NMR (CDCl_3_, 300 MHz): δ 7.45 (d, *J* = 8.7 Hz, 1H _Phenyl_), 6.97 (d, *J* = 8.7 Hz, 1H_Phenyl_), 6.01 (s, 1H, H_pyrazole_) 3.85 (s, 3H, Alkyl_,_). ^13^C­{^1^H} NRM (CDCl_3_, 75 MHz): δ 160.50 (C_quaternary,_ Ph), 145.40 (CH_pyrazole_), 126.95 (CH_Ar,_ Ph), 114.78 (CH_Ar,_ Ph), 93.13 (CH_pyrazole_), 55.57 (O–CH_3_). MW (C_10_H_9_N_5_O 215.211 g/mol). HRMS-DART­(+) high resolution, (C_10_H_9_N_5_O 215.11 g/mol); [M + H]^+^ = calculated 216.08853 *m*/*z* (40%), [M + H]^+^ = found 216.08775 *m*/*z* (100%). IR­(ϑ cm^–1^): 1027.99, 1054.59, 1113.00 (H_Ar_-Ph),1175.36 (N_3_ confirmation band), 1458.84 (Ph),1665.99 (C=NH), 2122.42 (N_3_ principal intense band), 2841.93 (O–CH_3_), 3258.23 (CH_Ar‑Ph_).

### Chemical Synthesis of 3-Azido-4-(4-chlorophenyl)-1*H*-pyrazole, **6**

Following the general procedure to obtain the desirable compound, 500 mg, 2.58 mmol of 4-(4-chlorophenyl)-1*H*-pyrazol-3-amine was reacted with NaNO_2_ (223 mg, 3.23 mmol) and subsequently with NaN_3_ (252 mg, 3.87 mmol) after isolation and purification a greenish solid corresponding to pure azido-pyrazole **6** was yielded. (490 mg, 87%). ^1^H NMR (CDCl_3_, 300 MHz): δ 7.69 (s, 1H, Hpyrazole), 7.50 (d, *J* = 8.7 Hz, 1H Phenyl), 7.33 (d, *J* = 8.7 Hz, 1H Phenyl). ^13^C­{^1^H} NRM (CDCl_3_, 75 MHz): δ 147.63 (C quaternary), 132.62 (C quaternary), 129.29 (C quaternary), 128.97 (CHAr, Ph), 127.94 (CHAr, Ph), 111.64 (CH pyrazole). MW (C_9_H_6_ClN_5_ 219.630 g/mol). HRMS-DART­(+), (C_9_H_6_ClN_5_ 219.63 g/mol); [M + H]^+^ = calculated 220.03900 *m*/*z* (100%), [M + H]^+^ = found 220.03805m/z (28%). IR­(cm^–1^): 864.61 (C–Cl), 1092.68, 1015.06 (doublet, CHAr), 1157.31 (N3 confirmation band), 1455.65 (Ph),1657.64 (C=NH), 2131.59 (N3 principal intense band), 2841, 3169.07 (CHAr-Ph).

### Chemical Synthesis of 5-Azido-3-(thiophen-2-yl)-1*H*-pyrazole, **7**

Following the general procedure to obtain the desirable compound, 500 mg, 3.02 mmol of 3-(thiophen-2-yl)-1*H*-pyrazol-5-amine was reacted with NaNO_2_ 261 mg, 3.78 mmol and subsequently with NaN_3_ 295 mg, 4.54 mmol, after isolation and purification a brownish solid corresponding to pure azido-pyrazole **7** was yielded. (493 mg, 85%). ^1^H NMR (CDCl_3_, 300 MHz): δ 10.07 (s, 1H, H_pyrazole, broad_), 7.35 (dd, *J* = 5.0, 1.2 Hz, 1H_thiophen‑2‑yl_), 7.27 (dd, *J* = 3.7, 1.2 Hz, 1H_thiophen‑2‑yl_), 7.09 (dd, *J* = 5.1, 3.6 Hz, 1H_thiophen‑2‑yl_), 6.09 (s, 1H, H_pyrazole_). ^13^C­{^1^H} NRM (CDCl_3_, 75 MHz): δ 149.19 (C_quaternary‑pyrazole_), 139.99 (C_quaternary_), 130.92 (C_quaternary_), 128.12 (CH_Ar,thiophen‑2‑yl_), 126.29 (CH_Ar,thiophen‑2‑yl_), 125.10 (CH_Ar,thiophen‑2‑yl_), 94.01 (CH_pyrazole_). MW (C_7_H_5_N_5_S 191.213 g/mol). HRMS-DART­(+), (C_7_H_5_N_5_S 191.21 g/mol); [M + H]^+^ = calculated 192.03439 *m*/*z*, [M + H]^+^ = found 192.03300 *m*/*z* (100%). IR­(cm^–1^): 1061.00, 1080.22, 998.28 (CH_Ar_), 1333.63 (N_3_ confirmation band), 2113.24 (N_3_ principal intense band), 3022.72, 3088.48, 3130.90 (CH_Ar_).

### Evaluation of Antifungal Activity

The antifungal activity was assessed by examining the impact of triazole and azido pyrazole derivatives on the radial growth of three phytopathogenic fungi: *Rhizoctonia* sp., *Fusarium oxysporum*, and *Alternaria alternata*. The agar dilution method^24^ involves the dissolution of compounds in methanol or acetone, followed by their combination with Potato Dextrose Agar and water. Each compound, with a concentration of 100 μg/mL, was meticulously prepared and placed into Petri dishes. Following this, the compounds were solidified, inoculated with fungal mycelium, and subsequently incubated at a temperature of 28 °C for a period of 12 days Captan 50 served as the positive control, while negative controls included PDA-water, PDA-methanol, and PDA-acetone. Bioassays were conducted with four replicates in a randomized design. Mycelial diameter measurements were taken daily over 7 days, calculating inhibition percentages compared to control cultures at specific intervals for each fungus.^24^

### Synergistic Effect of Compound **3** with Ag Nanoparticles (Ag-NPs)

Compound **3** was evaluated at a concentration of 10 μg/mL by agar dilution method Ag-NPs were added culture media at 1% of compound **3** concentration (100 ng/mL). Ag-NPs stock suspensions at 0.02 mg/mL with a particle size of 10 nm were used. The percentage of inhibition of each treatment was calculated using as negative control PDA-water and PDA-Ag-NPs at 100 ng/mL.

### Determination of Mean Inhibitory Concentration (IC_50_) Values

The determination of the IC_50_ values of the antifungal compounds **3**, **3** + AgNPs, **7**, and Captan 50 on the radial growth of the fungal pathogens was conducted using the agar dilution method at a concentration range of 5 to 100 μg/mL (27.02 to 540.34 μM) for compound **3** and **3** + AgNPs, (26.15 to 522.99 μM) for compound **7**, and **1** to 50 μg/mL (3.33 to 166.34 μM) for Captan 50 and by calculating the percentage of inhibition at each concentration.^24^

### Statistical Analysis

The data obtained from the evaluation of the antifungal activity were then subjected to one-way analysis of variance (ANOVA, α = 0.05), followed by Tukey’s multiple comparison tests. The statistical analysis of bioactivity data and the determination of IC_50_ values were performed using the GraphPad Prism software, version 5.00 (GraphPad Software Inc., San Diego, CA, USA). The data are expressed as the mean ± standard deviation (SD) or standard error (SE). In accordance with the methodologies employed by Sánchez-Fernández et al.,^24^ a *p* value of 0.05 or less was considered to indicate statistical significance.

### Protein Sequence Alignment

To identify conserved residues potentially involved in substrate interaction or molecular recognition, a multiple sequence alignment was performed using MEP36-like proteins from three phytopathogenic fungi. The sequences analyzed included *Rhizoctonia solani* (KAF8751731.1) and *A. alternata* (XP_018383305), both retrieved from the NCBI protein database, as well as *F. oxysporum*, obtained from a crystallographic structure available in the Protein Data Bank (PDB ID: 7QFY;)^25^ This latter sequence corresponds to a structurally resolved MEP36 protein, providing a reference for identifying functional residues.

All sequences were formatted in FASTA and submitted to the online version of MAFFT v7 using^26^ default parameters, which apply the FFT-NS-2 algorithm for efficient and accurate alignment of moderately similar sequences. MAFFT was selected due to its proven ability to detect conserved regions across homologous proteins.

The alignment was manually examined to identify residues potentially involved in ligand binding, as inferred from the crystallographic structure of the *F. oxysporum* MEP36 protein. Special attention was paid to positional correspondence and chemical conservation of key residues across the three sequences. This comparative approach enabled the assessment of functional conservation among MEP36 homologues from different fungal species, providing preliminary insights that may inform future studies, such as comparative structural modeling or molecular docking with shared ligands.

### Computational Details

Seven azido-pyrazole derivatives **1–7** and their corresponding amino-pyrazole precursors (**1P–7P)** were modeled and optimized in their geometries through DFT (B3LYP/6–31 + G­(d,p)) calculations using the Gaussian 16 suite of programs.^27^ Frequency calculations confirmed that all optimized structures corresponded to local minima on the potential energy surface by the absence of imaginary vibrational modes. Solvent effects (water) were incorporated using the SMD solvent model.^28^ NBO charges were computed during optimization and used for accurate partial atomic charge assignments in subsequent docking simulations.^29^

The biological target selected for docking was the Zn­(II)-metalloproteinase MEP36 from *F. oxysporum*, using the crystallographic structure deposited under PDB ID: 7QFY. The protein was prepared using AutoDock Tools,^30^ by removing crystallographic water molecules and cofactors, adding polar hydrogens, and assigning Gasteiger charges.^31^ The docking grid was centered on the catalytic pocket of the enzyme, including residues involved in substrate coordination and interaction with the catalytic Zn­(II) ion.

Molecular docking simulations were performed using four different programs: AutoDock Vina,^32^ mina,^33^ Qvina2,^34^ and Qvinaw.^35^ Ligands were prepared in PDBQT format with torsional flexibility assigned according to AutoDock Tools protocols. Each program generated multiple binding poses per ligand, with the top-ranked pose selected based on its predicted binding affinity (in kcal/mol).

To improve the reliability and consistency of the docking predictions, a consensus scoring approach was employed by calculating the average binding affinity across all four programs for the top-ranked pose of each ligand.

To assess the stability of the ligand–protein complex with the highest binding affinity, a 100 ns molecular dynamics (MD) simulation was performed using AmberTools.^36^ The complex was prepared using the ff14SB force field for the protein^37^ and GAFF for the ligand,^38^ solvated in a TIP3P water box with a 10 Å buffer,^38^ and neutralized by adding counterions. After a two-step energy minimization and gradual heating to 300 K, the system was equilibrated under NPT conditions. The production run was conducted at 300 K and 1 atm using a 2 fs time step. Electrostatic interactions were calculated using the Particle Mesh Ewald (PME) method, and bond constraints involving hydrogen atoms were enforced using the SHAKE algorithm. Trajectory analysis included root-mean-square deviation (RMSD), root-mean-square fluctuation (RMSF) and hydrogen bond analysis.

To complement the docking scores, binding free energies (Δ*G*_bind_) were estimated using the Linear Interaction Energy (LIE) method. Following standard single-trajectory protocols, the interaction energy between the ligand and the surrounding environment (protein + solvent) was calculated across the simulation frames. The electrostatic (*V*_ele_) and van der Waals (*V*_vdW_) interaction energies were extracted, and the final Δ*G*_bind_ was computed using the empirical equation: Δ*G*_bind_ = α⟨Δ*V*_vdW_⟩ + β⟨Δ*V*_ele_⟩ + γ, where the scaling parameters were set to α = 0.18, β = 0.50, and γ = 0.0 kcal/mol.

## Results and Discussion

### Synthesis of Azidopyrazole Compounds (**1**–**7**)

The synthesis of compounds **1**–**7** was successfully accomplished using a modified synthetic protocol.^23^ The process commenced with commercially available primary amine pyrazoles^39^ substituted at the C3 and C5 positions of the five-membered heterocycle (Scheme 2). During the procedure, an *in situ* diazonium salt intermediate was generated by reacting to the primary amine pyrazoles with NaNO_2_ in an acidic medium. Subsequent substitution of the diazonium group by an azido group from NaN_3_ afforded the target products.

**Scheme 2:**
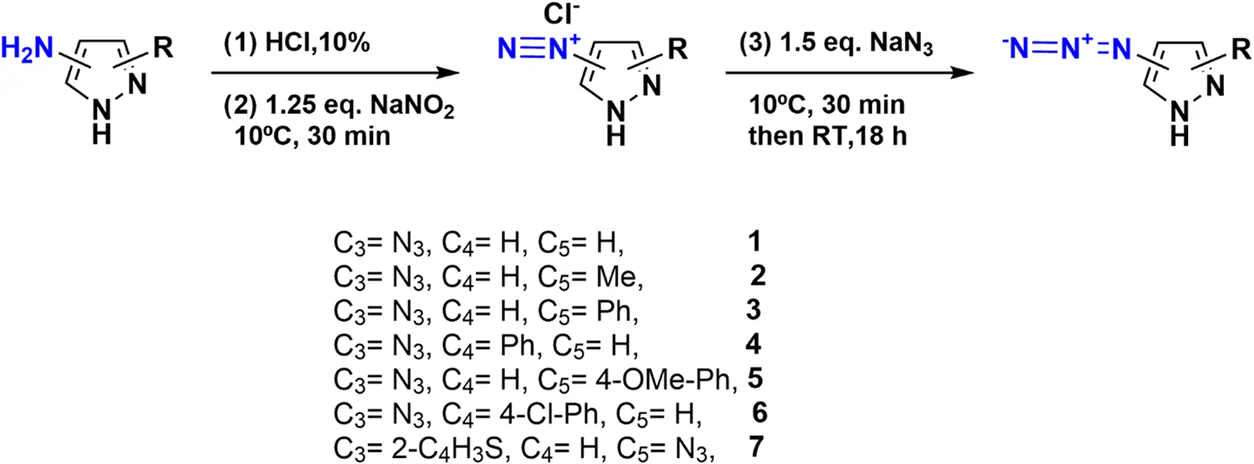
Synthesis of the Novel Azidopyrazoles **1–7** from Primary Amine Pyrazoles

Progress of the reactions was monitored via thin layer chromatography (TLC), and upon completion (i.e., full consumption of the starting material), the reactions were quenched with NaHCO_3_, thereby neutralizing the acidic pH to neutrality (pH 7.0–7.5). Product isolation was achieved through extraction with ethyl acetate. For the preparation of compounds **1–7**, independent batches (more than three per compound) were synthesized. Notably, for compounds **1–6**, extending the reaction time overnight (∼18 h) led to a modest increase in yield, although the azidation step typically required only three to 4 h for completion.

In contrast, for compound **7** (the thiophene-substituted azido pyrazole), extending the reaction beyond six to 8 h resulted in significant formation of an undesirable polymeric byproduct, adversely affecting overall yield. Single-step yields for compounds **1–7** ranged between 85% and 94% (to see details see Experimental Section).

### Characterization of Azidopyrazole Compounds **1**–**7**

The identity and purity of compounds **1–7** were confirmed by ^1^H, ^13^C NMR spectra, HRMS-ESI­(+) for **2–7**, HRMS-IE­(+) for **1** and FT-IR experiments (Figures S1–S28).

As anticipated, signals corresponding to the pyrazole frameworks were observed. The presence of pyrazole protons was detected in the range of 5.76 to 7.71 ppm, thereby indicating their aromatic nature, as illustrated in Table 1. For compounds **1–4** and **7**, the N–H proton of the pyrazoles manifested as broad signals ranging from 10.07 to 12.37 ppm on the ^1^H NMR spectra, indicative of their acidity character.

**Table 1:**
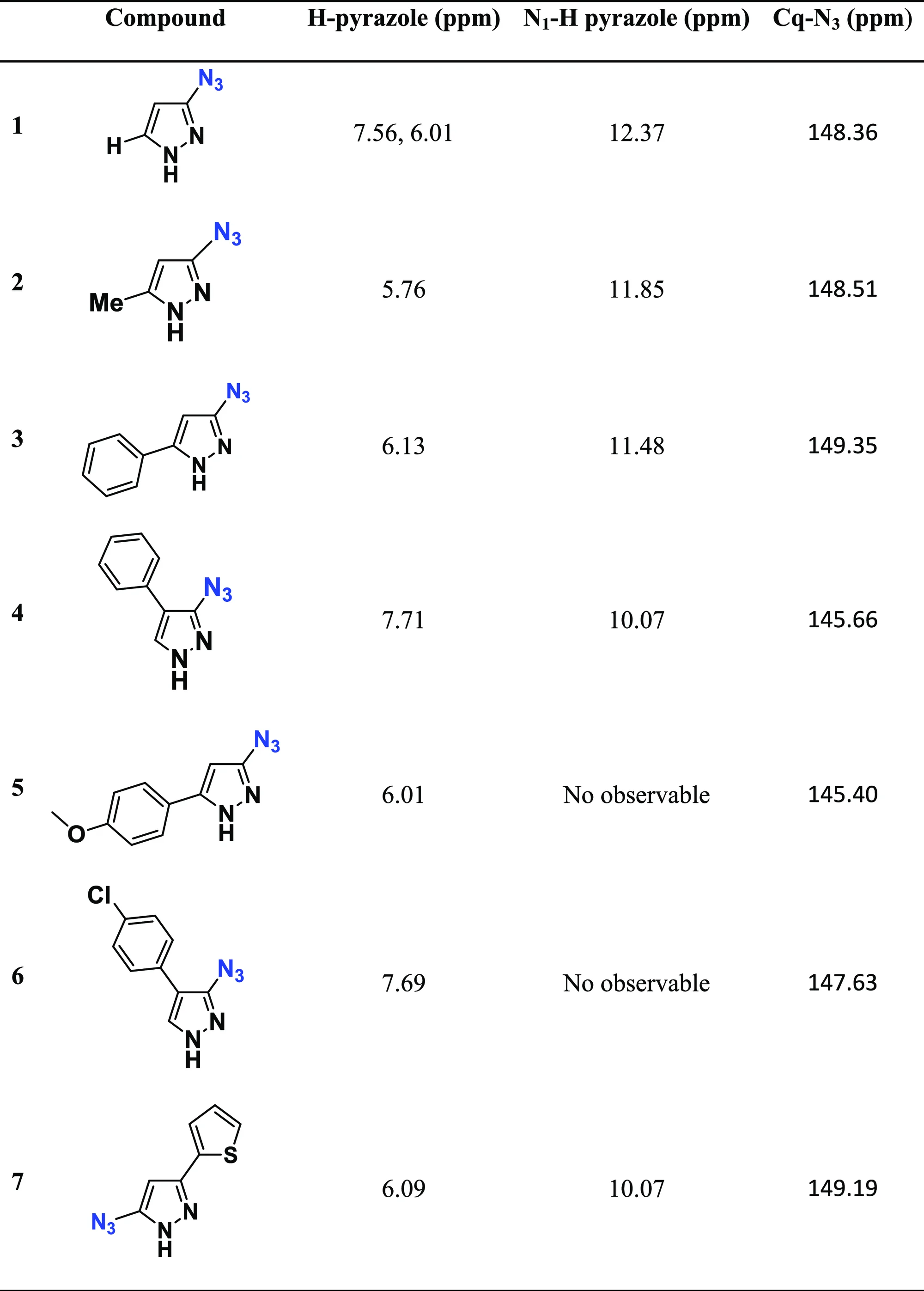
Selected Signals of Compounds **1–7** for ^1^H and ^13^C NMR at 300 MHz and 25°C in CDCl_3_

Moreover, the aromatic protons of the phenyl ring (compounds **3–6**) resonate at 7.43 and 7.41 ppm as multiplets within the aromatic zone of the spectra. In contrast, neither of the ^1^H NMR spectra of compounds **5** and **6** showed the signal for the pyrazole proton attached to the N1 position of the heterocyclic core. The absence of these signals is common for strongly acidic protons such as those found in pyrazoles and amines, as well as in other functional groups like alcohols and can also be explained by tautomeric equilibria.

The pyrazole proton of the compound manifests at 6.16 ppm as a singlet. Conversely, the connectivity patterns of pyrazoles **1–7** were ascertained through ^13^C NMR analysis, which yielded anticipated signals for the pyrazole carbons in all compounds.

Moreover, the connectivity patterns were determined by the presence of substituents, as evidenced by the ^13^C NMR spectra. For instance, the resonance of the quaternary carbon (Cq-N_3_) with the attached azido substituent (-N_3_) to the pyrazole core occurs within the range of 147.63 to 149.35 ppm (Table 1). Regarding the individual pattern of all other carbons for each compound in ^13^C NMR see the Supporting Information. Indeed, the synthesis of azidopyrazole derivatives has received minimal attention to date. Nevertheless, in 2020, Ewies^40^ derivatized azidopyrazole derivatives to yield complex azidopyrazoles with different functionalities, which have been identified as both cytotoxic compounds and antimicrobials. In comparison to that previous azidopyrazoles, the ^1^H NMR and ^13^C NMR characterization of compounds **1–7** is consistent with them.

The Direct Analysis in Real Time approach with positive polarity (MS-DART^+^) was used to conduct mass spectrometry experiments. These analyses indicated that the mass spectrum of compounds **1–7** corresponds to the anticipated molecular mass of the new azido pyrazole compounds.

High resolution mass spectra with positive scan of ESI were used to identify the protonated molecular ions for the corresponding compounds. AHRMS-EI­(+) spectra of the synthesized compounds are shown in Supporting Information.

Next, FT-IR spectra were measured for all compounds. The experimental results obtained from the Fourier-transform infrared (FT-IR) analysis have provided compelling evidence that can be used to identify the characteristic fingerprints of these compounds. These results indicate the presence of distinct functional groups (for more information see Supporting Information files). Concurrently, the FT-IR experiments have revealed the connectivity pattern of the compounds, with a particular emphasis on the direct detection of the azido group (-N_3_) on the compounds. Indeed, the asymmetric stretching band has been used as a distinct probe of the azido group due to its easy detection, its position where many other functional groups do not interfere, and its high level of transmittance on the IR spectra.^41^ The asymmetric stretching band for the azido group has been reported within the range of 2100–2260 cm^–1^ for a multitude of azido organic compounds. In this case, compounds **1–7** range 2111.19–2131.59 cm^–1^ (Table S1).

These experimental values provide unequivocal validation and confirmation of the installation of the azido group. This information aligns with the installation of the azido group. Furthermore, following the execution of earlier experiments, the FT-IR spectra of 1-azido adamantane was acquired. The compound under consideration exhibits a distinctly discernible absorption band at 2079.72 cm^–1^ attributable to the presence of an azido group which is very close to the values of the stretching band of the azido groups on the synthesized compounds. In addition to the direct observation of the azido group in the FT-IR spectra, the disappearance of the primary amine band on the precursors was also noted. For instance, the amino precursor **7P** has the amino group attached to its framework (Figure S29). The presence of the amino group can be discerned at a specific wavenumber of 3131 cm^–1^, as evidenced by the selected example depicted in SI, Figure S29. Therefore, the existence of the proposed azido pyrazole compounds **1–7** has been confirmed through a combination of multinuclear NMR analyses, mass spectrometry, and FT-IR experiments.

### Evaluation of Inhibitory Tests

The initial screening of the azido pyrazole compounds **1–7** was conducted at a concentration of 100 μg/mL, with the objective of assessing their efficacy against the fungal growth of *Rhizoctonia* sp., *F. oxysporum*, and *A. alternata* (Table S2). The screening process incorporated amino pyrazole precursors **3P** and **7P**, recognized as the most promising active precursors, along with the commercial fungicide Captan 50. This screening yielded significant findings, providing valuable insights into the condition’s characteristics. Amino pyrazole precursors **3P** and **7P** have demonstrated lower activity.

For instance, the commercially available compound 3-amino-5-phenyl pyrazole **3P** demonstrated activities against the three fungi, with the most effective result being 27.67% inhibition of *F. oxysporum*.

A comparable trend was observed with 3-tienyl-5-amino pyrazole **7P**, as its enhanced activity against *F. oxysporum* was recorded at 19.14% of inhibitory activity. Besides the screening of the amino pyrazole precursors. Regarding the newly synthesized compounds. For **1**, which contains no substituents in the pyrazole core, and **2**, which contains a methyl group on C5 of the pyrazole, exhibited very low activities.

In contrast, the arylated azido pyrazoles demonstrated notable inhibitory activity against the target fungi (compounds **3**, **4**, **6**, and **7**) (Figure 2). Indeed, the most favorable outcomes pertaining to the activity of compounds **3** and **7** resulted in 95 to 100% inhibition of the three fungi at 100 μg/mL (540.34 and 522.99 μM, respectively). A Structure Activity Relationship trend was obtained with the initial screening, and it is discussed in detail further with the aid of computational simulations. It is important to point out that compound **3**, an aryl azido-pyrazole, displays comparable activity to the commercially available fungicide Captan 50 at equivalent concentrations (100 μg/mL or 332.68 μM).

**Figure 2:**
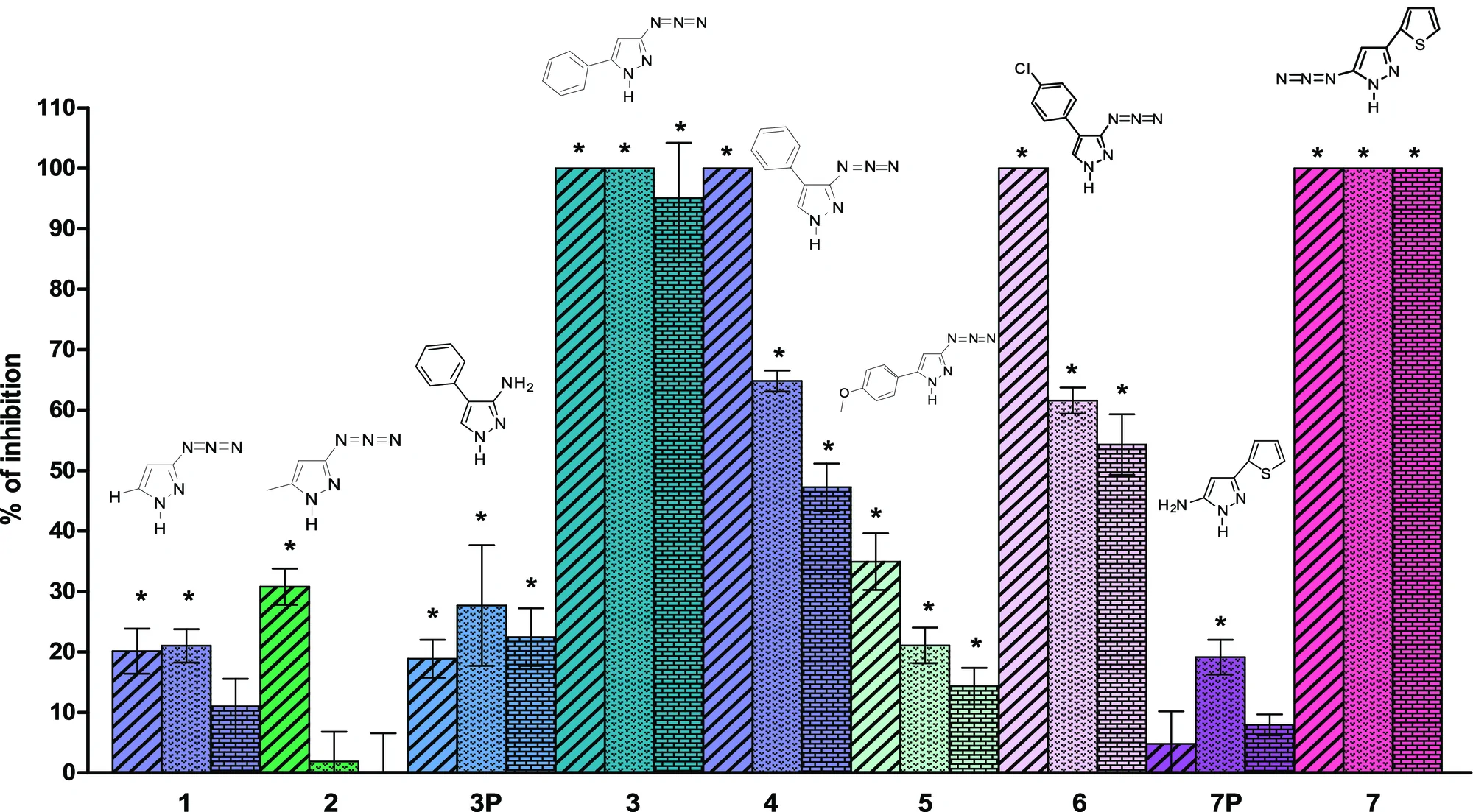
Antifungal activity of azido pyrazole derivatives **1–7** on the radial growth of *Rhizoctonia* sp. (
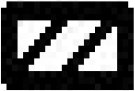
), *F. oxysporum* (
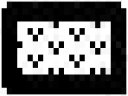
) and *A. alternata* (
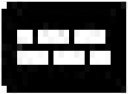
) at 100 μg/mL. Vertical bars represent mean ± SD, *n* = 4. (*) *p* < 0.05 compared with the negative control. Positive control Captan 50 inhibited completely the fungal growth at 100 μg/mL (332.68 μM).

This observation indicates a 100% inhibition of fungal growth. It was hypothesized that the use of silver nanoparticles would result in a substantial increase in the activity of the inhibitory essays.

In the experiment, the addition of 100 ng/mL of the silver nanoparticles (Ag-NPs), led to a substantial enhancement in the activity of compound **3** at a concentration of 10 μg/mL for *Rhizoctonia* sp.

The percentage of inhibition at this concentration was found to be 54.92% with AgNPs and 23.68% without AgNPs. This phenomenon can be attributed to a synergistic effect between compound **3** and AgNPs, yielding a satisfactory mixture for the purposes of this study.

Following the initial screening of compounds **1–7**, the selection of compounds **3** and **7** was based on the results of the antifungal activity at 100 μg/mL, which exhibited the greatest inhibitory effect on phytopathogenic fungi.

The determination of the IC_50_ value on fungal radial growth was conducted using a concentration range of 5 to 100 μg/mL for compounds **3** and **7** (27.02 to 540.34 and 26.15 to 522.99 μM, respectively), and 1 to 50 μg/mL (3.33 to 166.34 μM) for Captan 50. Table S3 compiled all concentrations used for the IC_50_ calculation.

Table 2 provides a synopsis of the IC_50_ values. Compounds **3** and **7** demonstrated higher IC_50_ values in comparison to Captan 50. Compound **3**, when used in conjunction with AgNPs, demonstrated a reduction in IC_50_ value for *Rizhoctonia* sp., as AgNPs themselves did not possess antifungal properties at a concentration of 100 ng/mL (1% of compound **3** concentration, Table S4). In the case of *F. oxysporum* and *A. alternata*, the addition of AgNPs did not improve the antifungal activity; instead, a decrease was observed. However, it has been demonstrated that the excessive utilization of commercial agrochemicals has led to the development of resistance among target fungi and the diminution of fungicide efficacy. Conversely, this practice has also contributed to detrimental effects on human health and environmental degradation.^42^

**Table 2:** Mean Inhibitory Concentration (IC_50_) Values of Compound **3**, **3** + NPs Ag, **7**, and Positive Control Captan 50 on Plant Pathogenic Fungi

Plant pathogen	3 (μg/mL ± SE^a^)	3 + NPs Ag (μg/mL ± SE^a^)	7 (μg/mL ± SE^a^)	Captan 50 (μg/mL ± SE^a^)
*Rhizoctonia* sp.	18.63 ± 1.08	9.65 ± 1.38	14.39 ± 1.09	4.26 ± 1.34
*F. oxysporum*	26.52 ± 1.14	56.52 ± 1.31	26.75 ± 1.36	9.77 ± 0.86
*A. alternata*	15.53 ± 1.14	31.57 ± 1.04	21.02 ± 1.37	2.57 ± 0.57

a SE: standard error.

The development of novel synthetic agrochemicals that are both effective and safe for the chemical protection of plants against fungal pathogens is imperative. The results obtained thus far support the designation of compound **3** as a promising candidate for future field tests in crop cultivation.

Figure 3 shows the growth inhibition of the phytopathogenic fungi caused by compound **3** at 5, 10, 15, and 30 μg/mL. The fungal growth inhibition exhibited a direct correlation with the concentration of the compound introduced to the culture medium.

**Figure 3:**
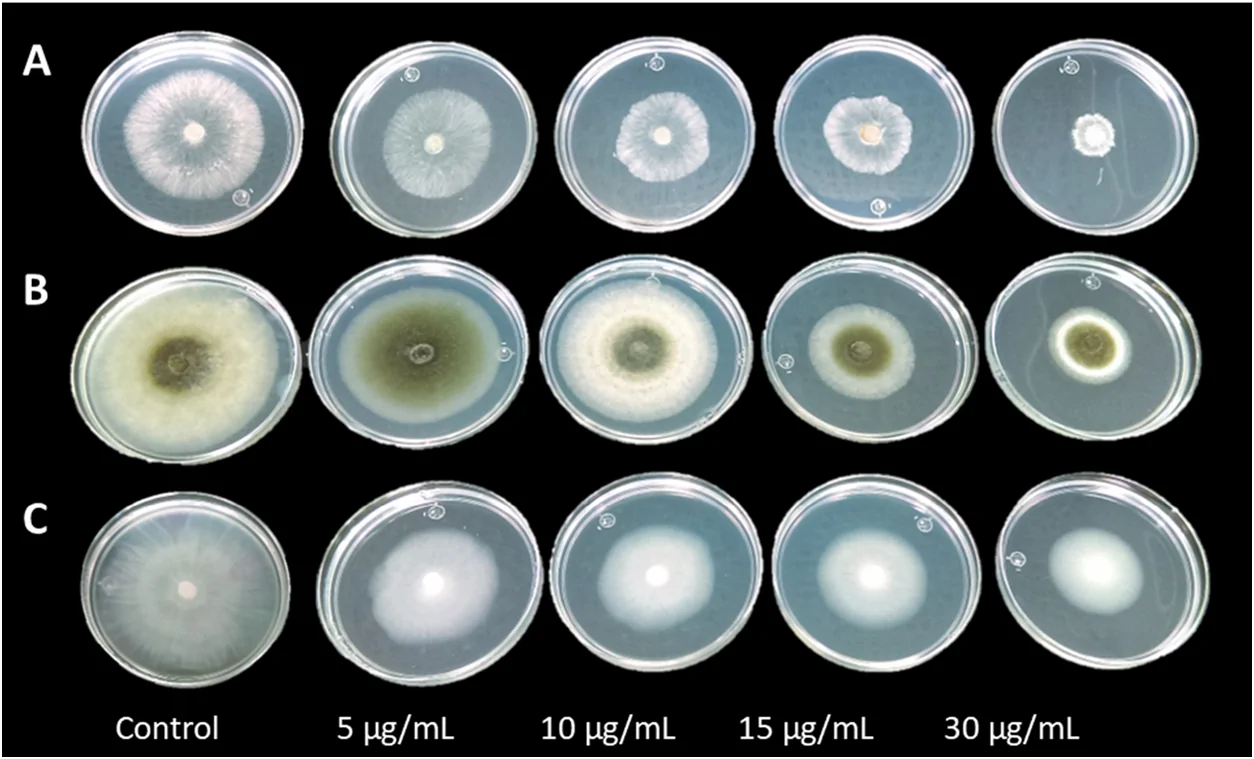
Inhibition on mycelial growth of the fungi *Rhizoctonia* sp. (A), *A. alternata*, (B) and *F. oxysporum* (C) caused by different concentrations of compound **3** at 2 days of growth for *Rhizoctonia* sp. and compound **7** days for *A. alternata* and *F. oxysporum*.

Concurrently, morphological alterations in the mycelium were discernible, as evidenced by a decline in mycelium density in *Rhizoctonia* sp. and an enhancement in pigmentation of the *A. alternata* colony (see Figure 3B).

### Molecular Alignment

Fungalysins represent a family of Zn­(II)-metalloproteinases (MEP36) identified in pathogenic fungi and certain bacteria. Although these proteases exhibit peptide motifs similar to those found in bacterial thermolysins, they share limited sequence homology with this extensively studied class of enzymes. Members of the M36 family function as extracellular metalloproteinases and play a significant role in the pathogenicity and virulence of *F. oxysporum*. The active site of MEP36 is defined by the Glu185 residue, while His184 and His188 are key residues involved in Zn­(II) coordination and are essential for protease functionality.

Given the critical nature of these residues and the enzyme’s contribution to fungal virulence, MEP36 is a promising molecular target, especially considering its role in degrading plant chitinases produced as a defense response.^43^

MEP36 enzymes are exclusive to fungi, and multiple sequence alignment reveals strong conservation within the catalytic core’s active site regions.^44^ These findings provide insight into the potential mechanism of action for pyrazole compounds against pathogens such as *F. oxysporum*, *A. alternata*, and *Rhizoctonia* species. Notably, the binding sites in *F. oxysporum* and *A. alternata* demonstrate substantial overlap, which may explain comparable inhibition test outcomes. Conversely, significant differences in the binding site alignments between *Rhizoctonia* and the other two fungi correspond to improved inhibition results, suggesting alternative binding interactions and potentially stronger compound affinities.^45^ Specifically, the sequence alignment identifies residue 275 as a point of divergence between *Rhizoctonia* sp. and the other fungi. Because the adjacent Val274 is the critical π-alkyl contact stabilizing compound **3**, this substitution at position 275 likely alters the local steric environment and flexibility of the hydrophobic pocket. This structural perturbation mechanistically explains the enhanced binding affinity and distinct IC_50_ trends observed for compound **3** against *Rhizoctonia* sp. relative to *F. oxysporum* and *A. alternata*.

The crystal structure of MEP36 is available in the Protein Data Bank (PDB) and was utilized in the present computational studies. Metalloproteases (MEPs) encompass several families of hydrolytic enzymes, most of which are Zn­(II)-dependent due to the presence of two histidine residues forming a HEXXH motif, as is the case for the M36 enzyme analyzed here (PDB ID: 7QFY). This motif consists of H184, E185, Y186, T187, and H188. *Previous re*search has explored the functions of various metalloproteinases, including MEP36, MEP35, and MEP43, across different pathogens.^46^ For instance, *F. oxysporum* produces an MEP36 protein, FoMEP1, that operates alongside the serine protease FoSep1 to degrade tomato-derived chitinases.^47^ Notably, azido pyrazoles specifically those substituted with aryl groups at their five-membered framework positions (**3**, **5**, **7**) have demonstrated promising inhibitory activities, exhibiting high affinity for both Zn­(II)-binding sites (H184 and H188) and the active site E185 Figure 4.

**Figure 4:**
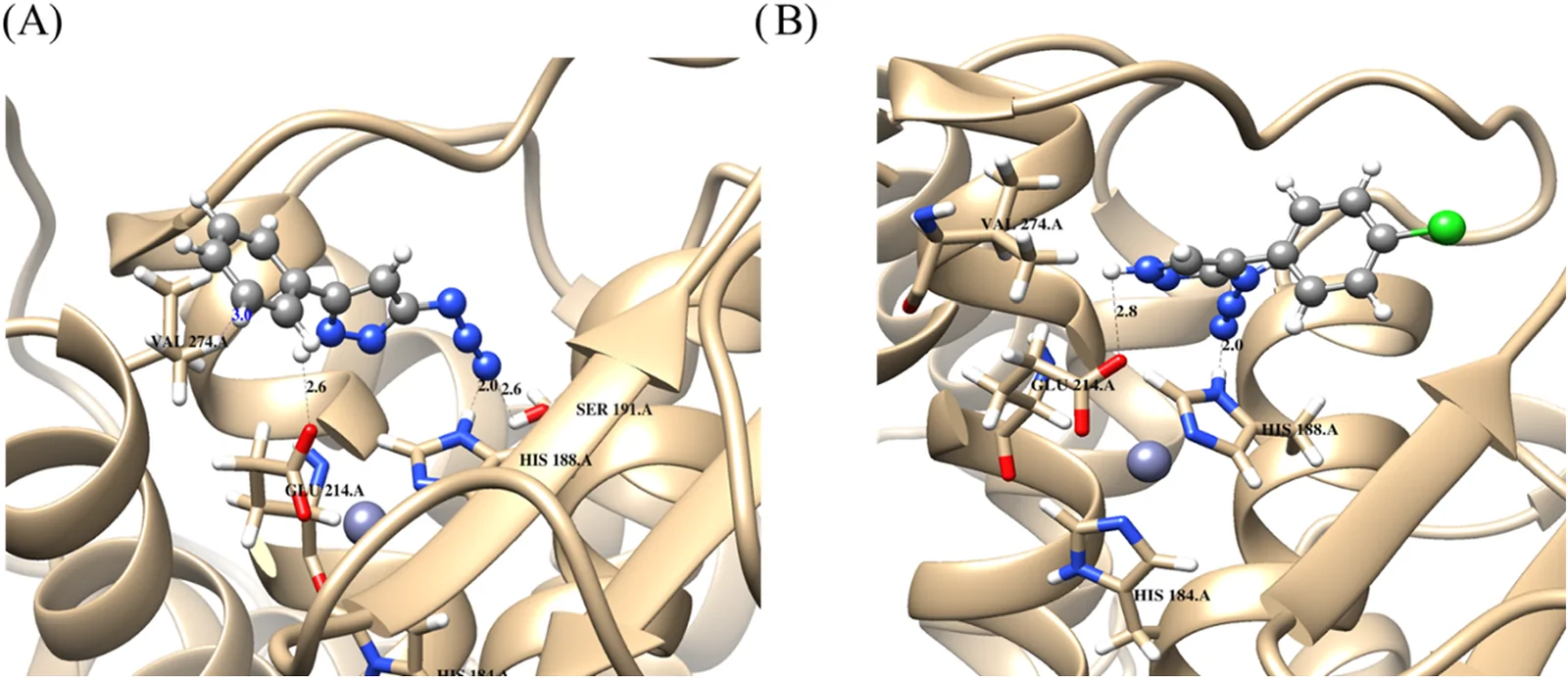
Binding site interactions of azido-pyrazole compounds: (A) compound **3** and (B) compound **6** within the active site of MEP36. Hydrogen bonds are shown in black and π-alkyl interaction in blue, with distances indicated in angstroms (Å).

### MEP36 Enzyme

Analysis of protein sequences shows that *F. oxysporum* and *A. alternata* share similar binding sites due to coinciding residues, in contrast with *Rhizoctonia*, which differs at positions 148, 181, 249, and 275 (Figure 5A–B). All three species display the conserved HEXXH Zn­(II)-binding motif and enzyme active site. Sequence differences may reflect changes in ligand-enzyme interactions. Figure 5 presents the MEP36 alignment, highlighting docking site selection based on residue function.

**Figure 5:**
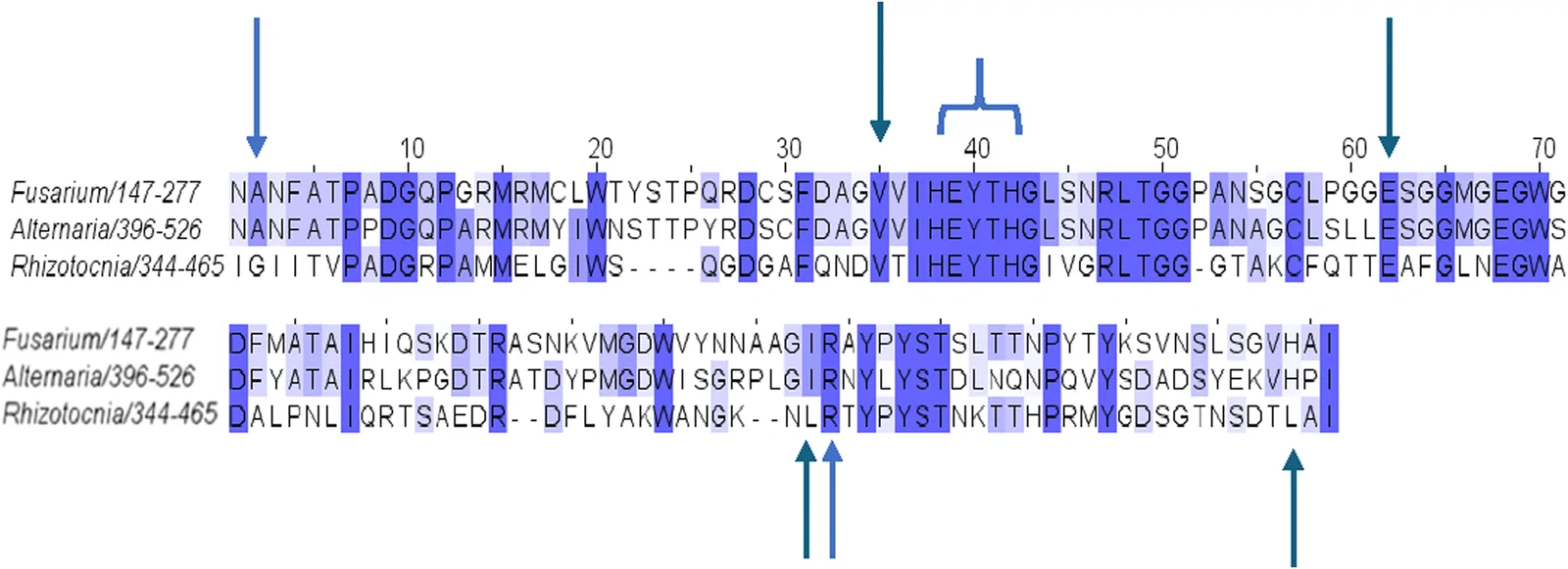
MEP36 alignment of *F. oxysporum*, *Rhizoctonia* and *A. alternata* is shown presented in the figure. The binding sites of the compounds with the enzyme are represented with blue arrows and the catalytic site with a square bracket.

Figure 6 shows the 3D structure of MEP36, with molecular docking targets marked in orange, the active site Glu 185 in pink, and Zn­(II)-binding His 184 and His 188 in yellow.

**Figure 6:**
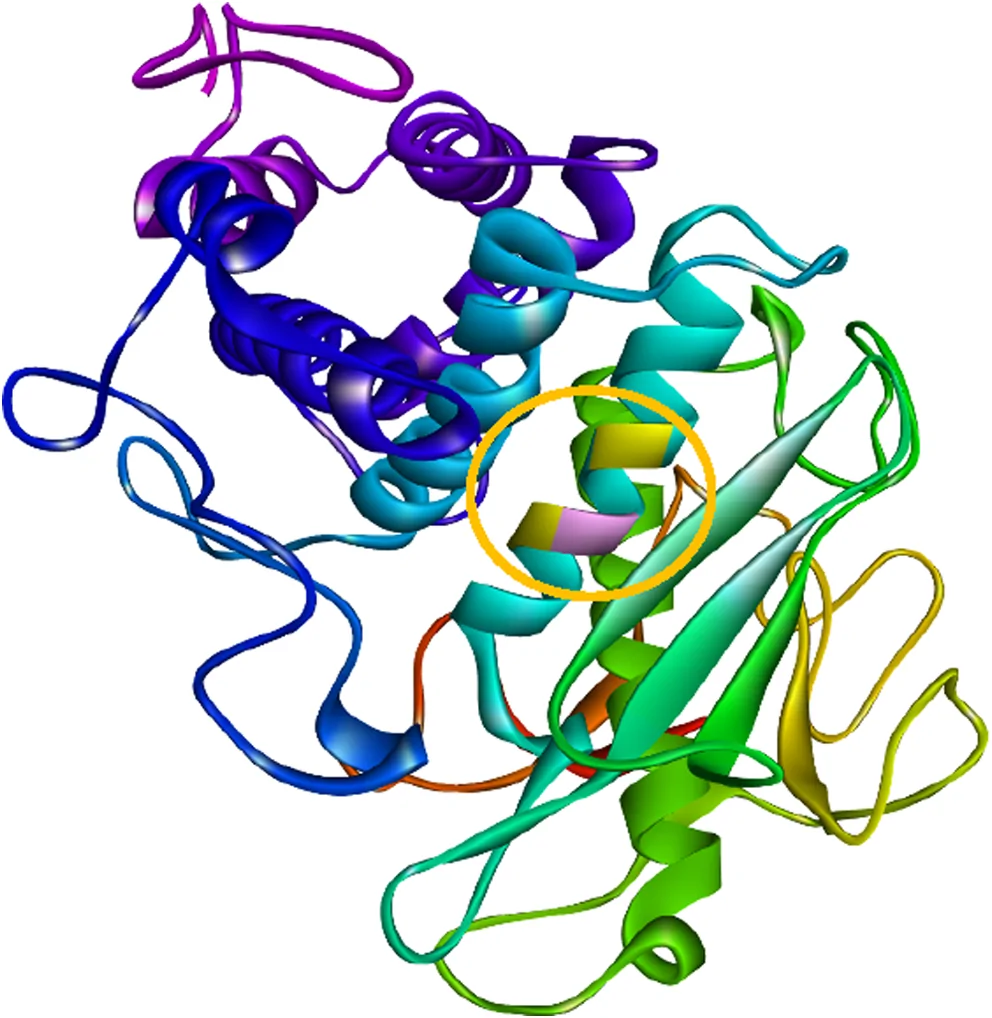
Binding sites. 3D representation of the MEP36 enzyme in blue color. We can observe, in pink, the active site of m36, represented by residue Glu 185, inside the orange circle. In yellow, we see the Zn­(II) binding sites in residues His 184 and 188, located within the yellow circle.

### Molecular Docking

To evaluate how azidation affects molecular binding affinity, azido-pyrazole compounds **1–7** and their amino precursors (**1P–7P**) were docked to Zn­(II)-metalloproteinase MEP36 (PDB: 7QFY) (Tables S5–S6).

Table 3 summarizes the consensus docking scores. The azido derivatives consistently showed more favorable (more negative) binding energies than their amino counterparts, indicating improved interaction with the catalytic site. Notable examples include compounds **3** (−6.8 vs −6.1 kcal/mol), **5** (−6.7 vs −6.4 kcal/mol), and **7** (−6.3 vs −5.8 kcal/mol). The greatest binding affinity improvements occurred in compounds with initially lower affinities, such as compound **1** (−5.3 vs −4.1 kcal/mol) and compound **2** (−5.7 vs −4.5 kcal/mol). Overall, azidation enhances binding affinity, especially in low-affinity scaffolds, with a consistent trend observed across the series. Moreover, all azido derivatives outperformed the azide anion itself, which exhibited a weighted binding affinity of −3.0 kcal/mol.

**Table 3:**
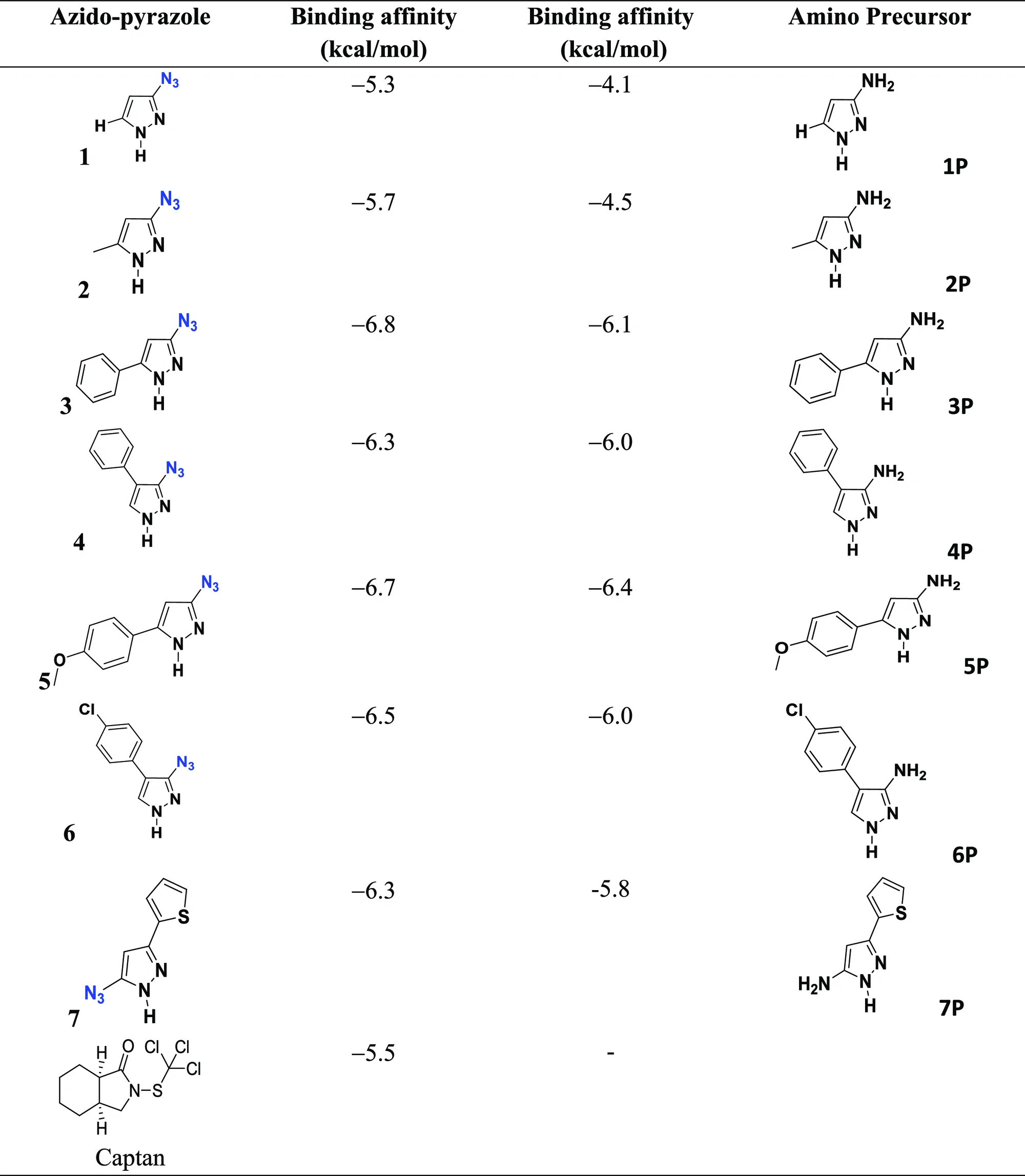
Consensus Binding Affinities (kcal/mol) Obtained by Molecular Docking for Azido-Pyrazole Compounds **1–7** (azide) and Their Corresponding Amino Precursors **1P-7P** (Amino Precursor) against the Zn­(II) Metalloproteinase MEP36 from *F. oxysporum* (PDB ID: 7QFY)

As depicted in Figure 7 and Figure S30–S32, all pyrazole-derived compounds demonstrate a comparable binding configuration within the active site of the MEP36 metalloproteinase. Molecular docking analyses indicate that each ligand occupies the same binding pocket, stabilized by two to three hydrogen bonds with catalytic residues. Glu214 (2.6 Å), His188 (2.0 Å), and Ser191 (2.6 Å)—the latter two involved in Zn­(II) coordination—act as points of interaction for the ligands. The consistent interaction pattern indicates that variations in binding affinity among the complexes are mainly influenced by the type and spatial arrangement of substituents on the pyrazole group.

**Figure 7:**
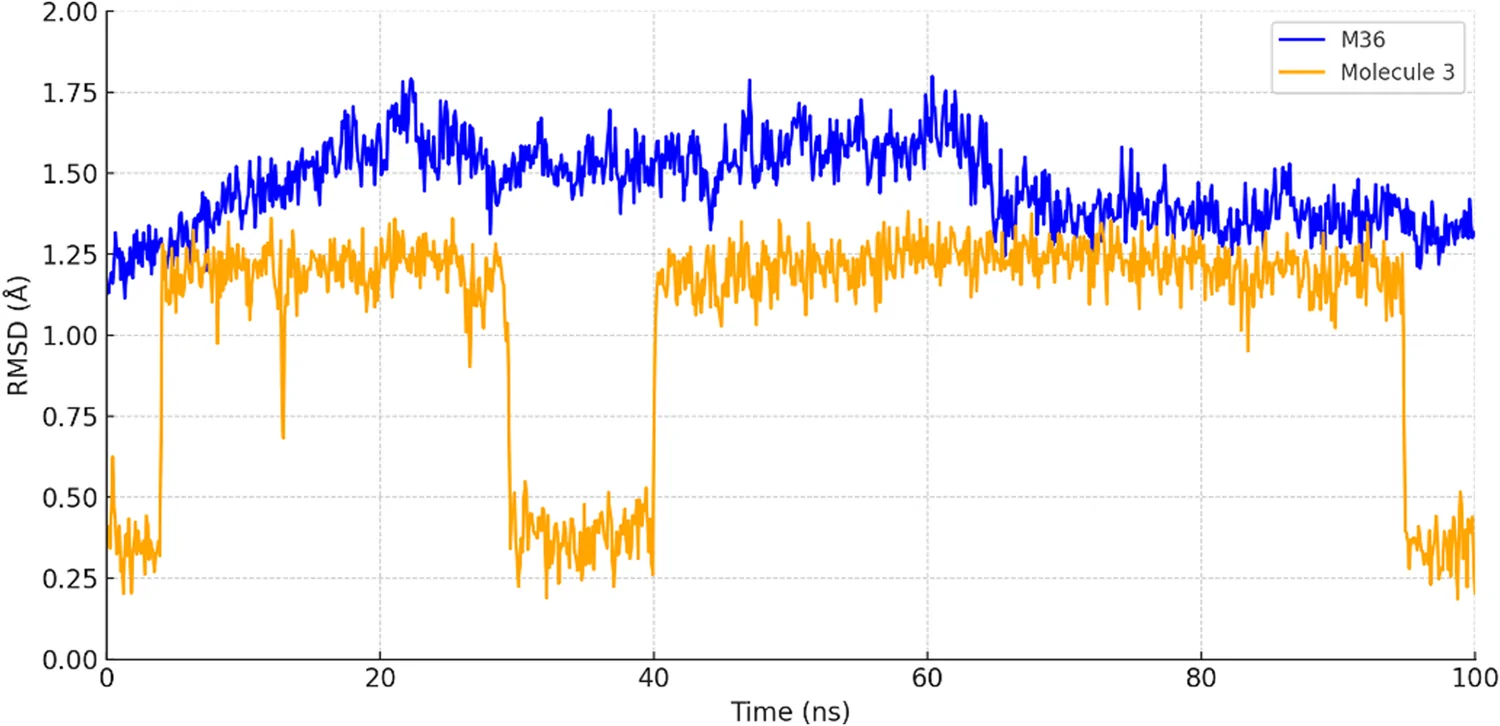
Root Mean Square Deviation (RMSD) profiles over 100 ns of molecular dynamics simulation for complexes MEP36 (blue) and **3** (orange). RMSD values are reported in Angstroms as a function of time (ns).

For compound **3**, its higher binding affinity is attributed to favorable π-alkyl interactions between its phenyl group and the Val274 residue, which contributes to the stabilization of the hydrophobic cavity. The π-alkyl interaction observed for compound **3** is absent in compounds **1**, **2**, **4**, and **6** due to the arrangement of their substituents, whereas compound **5** exhibited an interaction pattern and binding affinity similar to those of compound **3**. Notably, amino acid sequence alignment reveals that the residue corresponding to Val274 in *F. oxysporum* is replaced by Thr274 in *Rhizoctonia*, while the adjacent residue, His275, is replaced by Leu275. These sequence substitutions could contribute to differences in the physicochemical environment of the binding site, thereby influencing the interaction with the ligand. However, the observed IC_50_ values do not show a direct correlation with the conservation of Val274 and His275, given that *A. alternata* and *F. oxysporum* conserve these residues yet exhibit distinct antifungal activities. Although these residues may contribute to compound binding, they are not the sole determinants of the observed species-dependent activity.

Other sequence differences within or near the binding region, as well as species-specific biological factors, could also contribute to the differences in antifungal activity.

### RSMD Molecular Dynamic Simulations

RMSD profiles from molecular dynamics simulations show that MEP36 maintains structural stability over 100 ns, with RMSD values between 1.1 and 1.5 Å.

Compound **3**, the top ligand, displays moderate RMSD fluctuations (0.25–1.25 Å) but remains stable in the active site.

The abrupt drops in RMSD observed during the trajectory (e.g., at ∼30 ns and ∼95 ns) correspond to transient rotameric adjustments of the phenyl ring, which briefly samples an alternative hydrophobic contact before returning to its stable π-alkyl interaction with Val274. These fast structural realignments do not disrupt the core hydrogen bond network, confirming a stable binding mode.

These findings indicate a strong and persistent interaction between compound **3** and MEP36, supporting its potential as an effective inhibitor.

Furthermore, the Linear Interaction Energy (LIE) estimations calculated from the trajectory support these findings. The interaction energy components highlight that the binding of compound **3** is predominantly driven by van der Waals interactions (⟨*V*_vdW_⟩ = −50.61 kcal/mol), which is consistent with the critical π-alkyl interactions observed with Val274, compared to a smaller electrostatic contribution (⟨*V*_ele_⟩= −0.64 kcal/mol). The resulting free energy of binding (Δ*G*_bind_) of −8.46 ± 0.45 kcal/mol corroborates the thermodynamic stability predicted by molecular docking.

As illustrated in Figures 7–8, three principal hydrogen bond distances were tracked throughout a 100 ns molecular dynamics simulation. The GLU214 O···H (pyrazole) interaction (black curve) remained highly stable (∼2.2–2.5 Å), reflecting a robust and persistent contact that effectively anchors the ligand within the binding pocket. The SER191 O···N (azide) interaction (red curve) exhibited moderate stability (∼3.0–4.5 Å), indicating its function as a secondary stabilizing factor. Conversely, the HIS188 NH···N (azide) interaction (green curve) displayed greater variability (∼4.0–6.5 Å), which is consistent with transient bonding behavior.

**Figure 8:**
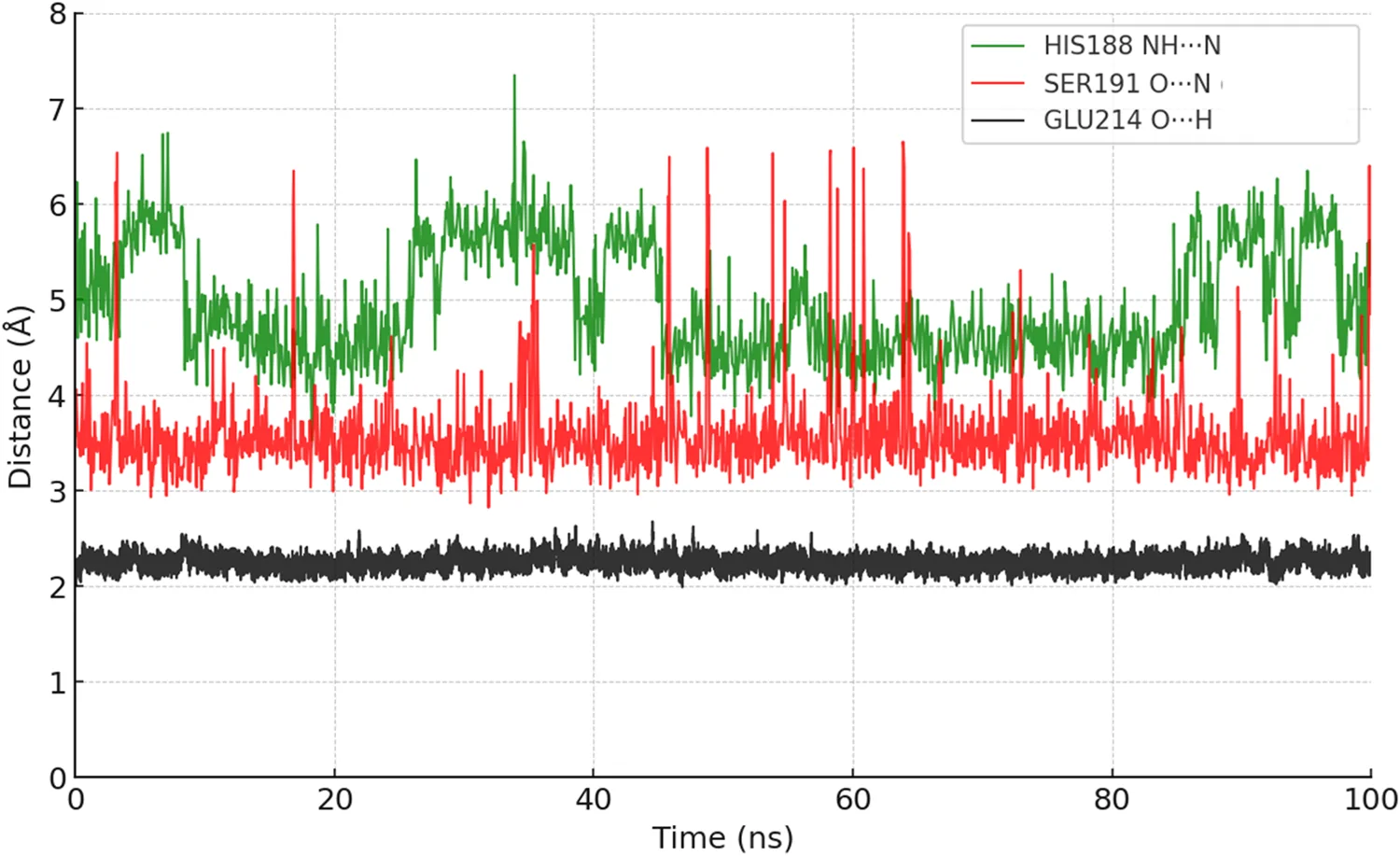
Time evolution of key hydrogen bond distances during the molecular dynamics between molecules **3** and MEP36. The green curve corresponds to the interaction between HIS188 NH···N (azide), the red curve shows SER191 O···N (azide), and the black curve depicts GLU214 O···H (pyrazole). Distances are shown in angstroms (Å) over a 100 ns simulation window.

This variability may stem from the intrinsic flexibility of the azide moiety and the propensity of HIS188 to coordinate with the Zn­(II) center, as inferred from the weaker interactions observed during the molecular docking analysis. Collectively, these findings support a dual anchoring mechanism characterized by dynamic modulation at the azide terminus.

## Conclusions

This study presents a short yet conclusive SAR model consisting of seven novel azido pyrazoles, which were evaluated for antifungal activity against three phytopathogenic fungi*:* *F. oxysporum*, *Rhizoctonia sp*., and *A. alternata*. Among these compounds, analogs **3** and **7** showed notable growth-inhibitory effects with mean IC_50_ values for radial fungal growth as follows: *F. oxysporum* (26.52 ± 1.14 and 26.75 ± 1.36 μg/mL); *Rhizoctonia sp*. (18.63 ± 1.08 and 14.39 ± 1.09 μg/mL); and *A. alternata* (15.53 ± 1.14 and 21.02 ± 1.37 μg/mL), respectively.

A significant synergistic effect was observed when compound **3** (10 μg/mL) was combined with Ag-nanoparticles (10 nm, 100 ng/mL).

The experimental findings from this study were corroborated by computational simulations, encompassing molecular docking and dynamic studies. These findings suggest that compounds **3** and **7** have the potential as candidates against plant pathogenic fungi and provide molecular insights into its probable mode of action in *F. oxysporum*.

Furthermore, this study showed that atom economy can be used to design simple small molecules in one step, leading to promising new fungicides. Using the rationalized SAR approach, novel azido pyrazoles were developed and tested as antifungals. These efforts advance the search for effective, environmentally friendly agrochemicals for plant protection and resistance management.

## Supplementary Material



## Notes

^1^H, ^13^C NMR, HRMS, and IR-ATR spectra for all compounds, docking calculations, and biological studies (PDF)
