# Supplementary material for: Synthesis, SAR Analysis, and Antifungal Potency against Plant Pathogenic Fungi of New Azidopyrazoles
Source: ACS Omega. 2026 Sep 22;11(39):60170–83. doi: 10.1021/acsomega.6c07653 (PMC13647939; doi:10.1021/acsomega.6c07653)
Supplement: Supplementary file 1 [file ao6c07653_si_001.pdf]

Synthesis, SAR Analysis, and Antifungal Potency against Plant Pathogenic Fungi of New Azidopyrazoles

Daniel Canseco-González[1], Rosa E. Sánchez-Fernández[2], Elizabeth Navarro-Cerón[2], Stephania Olivares-Sánchez[2], Alejandro O. Viviano-Posadas[3], Alejandro Dorazco-González[3*], Ricardo Parra Unda[4], Gilmar López-Armenta[4], Adrián L. Orjuela[5], and Jorge Alí-Torres[6].*

[1]SECIHTI-Laboratorio Nacional de Investigación y Servicio Agroalimentario y Forestal
Universidad Autónoma Chapingo Texcoco de Mora, CP 56230. México.
[2]Laboratorio Nacional de Investigación y Servicio Agroalimentario y Forestal, Universidad Autónoma Chapingo Texcoco de Mora, CP 56230. México.
[3]Instituto de Química, Universidad Nacional Autónoma de México, Circuito Exterior, Ciudad Universitaria, México CDMX, CP 04510, México.
[4]Unidad de Investigaciones en Salud Publica "Dra. Kaethe Willlms", Facultad de Ciencias Químico-Biológicas, Universidad Autónoma de Sinaloa, Culiacán, Sinaloa, CP. 80013, México.
[5]Instituto de Investigaciones Científicas y Servicios de Alta Tecnología (INDICASAT AIP), Panamá City, Panamá.
[6]Departamento de Química, Universidad Nacional de Colombia - Sede Bogotá, 111321, Colombia.
Corresponding autor: D. C.-G. jdcanseco@secihti.mx, A. D.-G. adg@unam.mx

SUPPLEMENTARY MATERIAL

Fig. S1 ^{1}H-NMR of compound **1** in $CDCl_3$ at 25ºC.

Fig. S2 ^{13}C-NMR of compound **1** in $CDCl_3$ at 25ºC.

Fig. S3 HRMS-ESI(+) experiment of compound **1**.

Fig. S4 FT-IR experiment of compound **1**.

Fig. S5 ^{1}H-NMR of compound **2** in $CDCl_3$ at 25ºC.

Fig. S6 ^{13}C-NMR of compound **2** in $CDCl_3$ at 25ºC.

Fig. S7 HRMS-ESI(+) experiment of compound **2**.

Fig. S8 FT-IR experiment of compound **2**.

Fig. S9 ^{1}H-NMR of compound **3** in $CDCl_3$ at 25ºC.

Fig. S10 ^{13}C-NMR of compound **3** in $CDCl_3$ at 25ºC**.**

Fig. S11 HRMS-ESI(+) experiment of compound **3**.

Fig. S12 FT-IR experiment of compound **3**.

Fig. S13 ^{1}H-NMR of compound **4** in $CDCl_3$ at 25ºC.

Fig. S14 ^{13}C-NMR of compound **4** in $CDCl_3$ at 25ºC.

Fig. S15 HRMS-ESI(+) experiment of compound **4**.

Fig. S16 FT-IR experiment of compound **4**.

Fig. S17 ^{1}H-NMR of compound **5** in $CDCl_3$ at 25ºC.

Fig. S18 ^{13}C-NMR of compound **5** in $CDCl_3$ at 25ºC.

Fig. S19 HRMS-ESI(+) experiment of compound **5**.

Fig. S20 FT-IR experiment of compound **5**.

Fig. S21 ^{1}H-NMR of compound **6** in $CDCl_3$ at 25ºC.

Fig. S22 ^{13}C-NMR of compound **6** in $CDCl_3$ at 25ºC.

Fig. S23 HRMS-ESI(+) experiment of compound **6**.

Fig. S24 FT-IR experiment of compound **6**.

Fig. S25 ^{1}H-NMR of compound **7** in $CDCl_3$ at 25ºC.

Fig. S26 ^{13}C-NMR of compound **7** in $CDCl_3$ at 25ºC.

Fig. S27 HRMS-ESI(+) experiment of compound **7**.

Fig. S28 FT-IR experiment of compound **7P**.

Fig. S29 FT-IR experiment of compound **7**.

Fig. S30 Image of silver nanoparticles at a scale of 50nm and 100 nm by TEM.

Fig. S31 Binding site interactions of azido-pyrazole compounds: **1** (blue), **2** (pink), **3** (green), **4** (orange), **5** (white), **6** (magenta), and **7** (yellow) within the active site of MEP36. Hydrogen bonds are shown in black, with bond distances indicated in angstroms (Å).

Fig. S32 Binding site interactions of the amino-pyrazole precursors **1P-7P**: A (blue), B (pink), C (green), D (orange), E (white), F (magenta), and G (yellow).

Fig. S33 Binding site interactions of the anion azide. within the active site of MEP36. Hydrogen bonds are shown in black, with bond distances indicated in angstroms (Å).

Table S1 Asymmetric stretching band of compounds **1-7** and 1-azido adamantane in FT-IR experiments.

Table S2 Antifungal activity of azido pyrazole derivatives **1 – 7** and precursors on the radial growth of *Rhizoctonia* sp., *Fusarium oxysporum* and *Alternaria alternata* at 100 µg/mL.

Table S3 Concentrations used for the IC_{50} calculation.

Table S4 *In vitro* IC_{50} values of synthesized compounds **3**, **7** with or without silver nanoparticles.

Table S5 Docking scores obtained with AutoDock Vina, Smina, Qvina2, and Qvinaw for azido-pyrazole compounds against MEP36 (PDB ID: 7QFY), along with simple and extended consensus values (kcal/mol).

Table S6 Docking scores obtained with AutoDock Vina, Smina, Qvina2, and Qvinaw for amino-pyrazole precursors against MEP36 (PDB ID: 7QFY), along with simple and extended consensus values (kcal/mol).

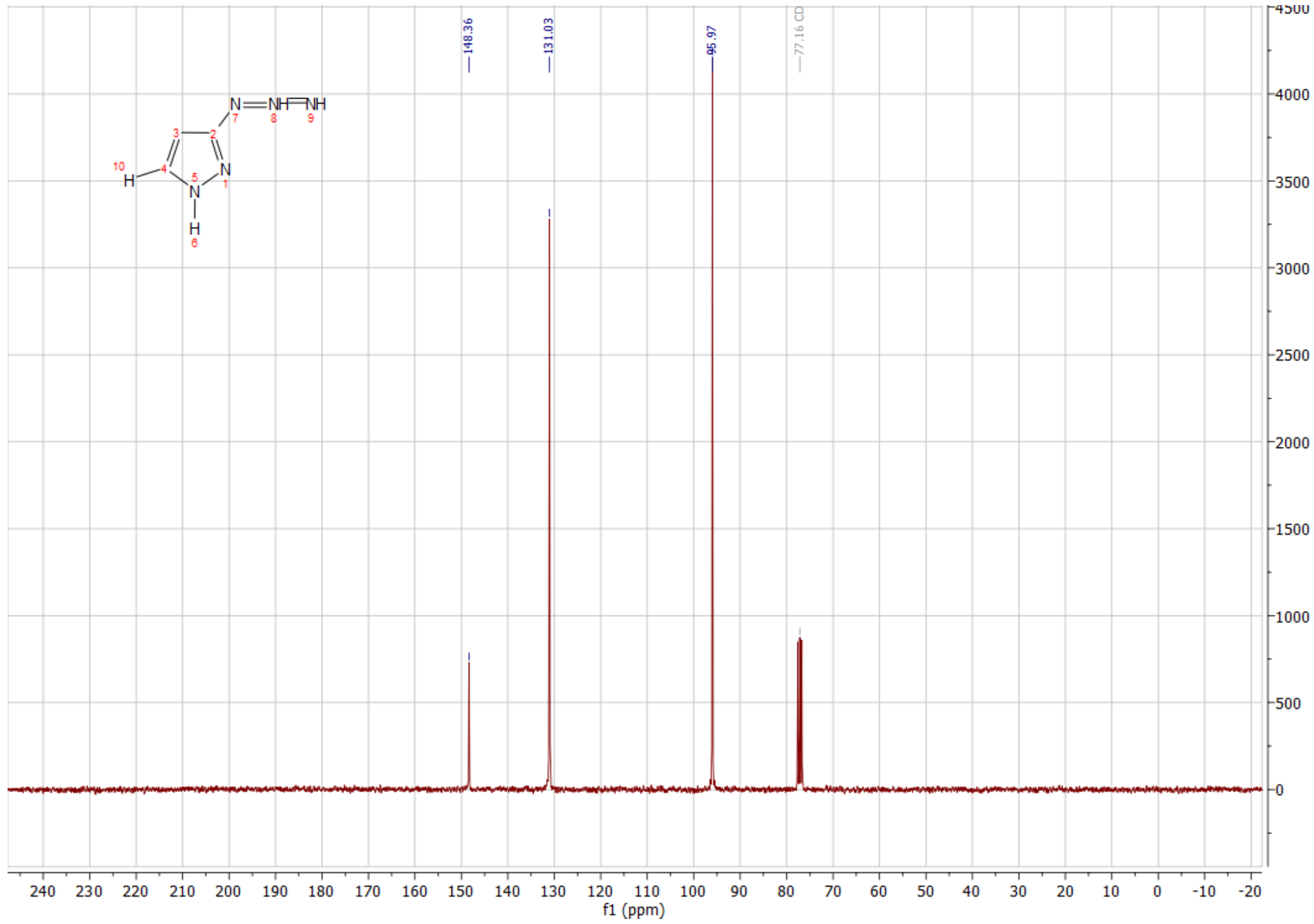

Fig. S1 ^{1}H-NMR of compound **1** in $CDCl_3$ at 25 °C.

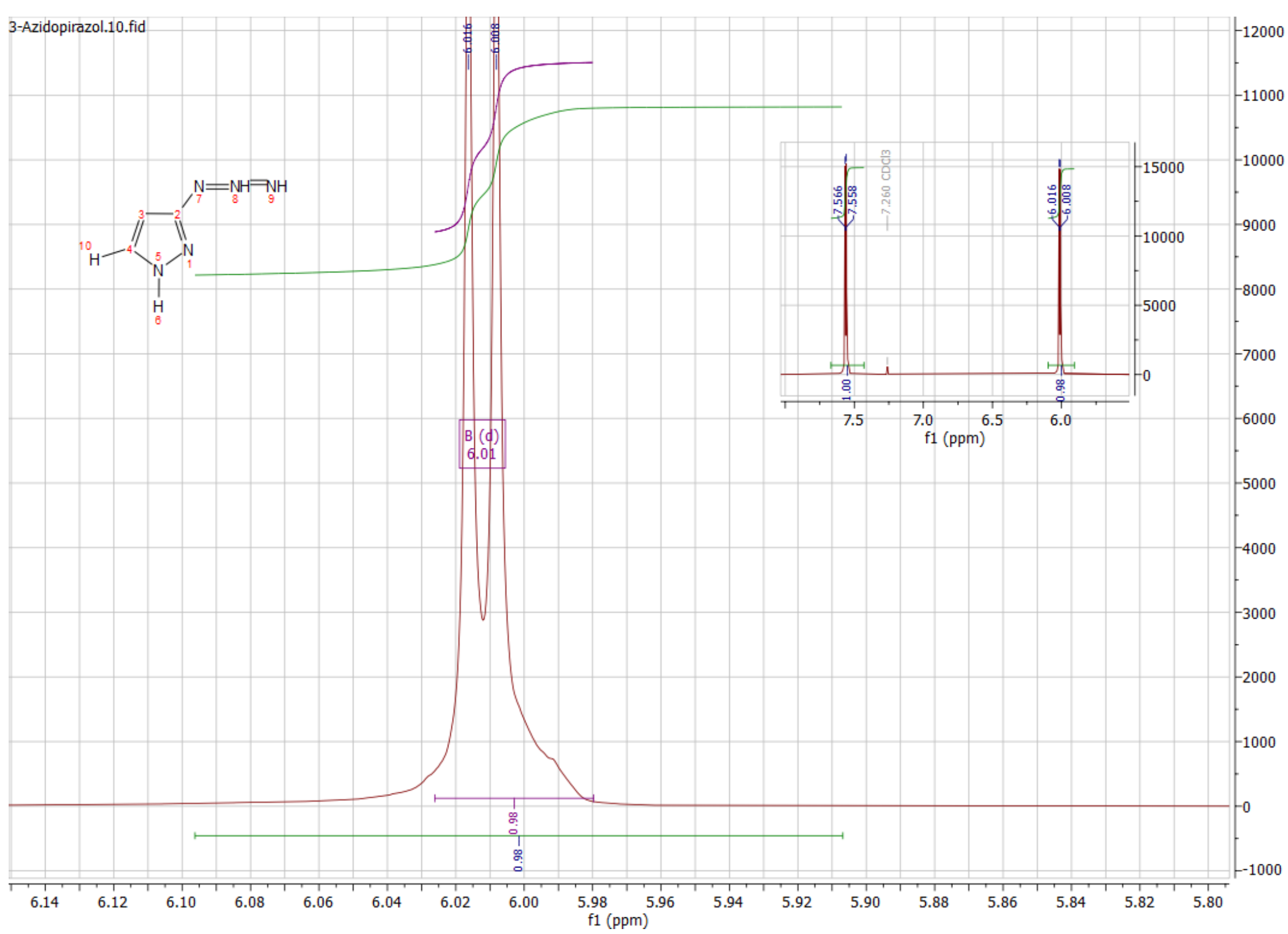

Fig. S2 ^{13}C-NMR of compound **1** in $CDCl_3$ at 25 °C.

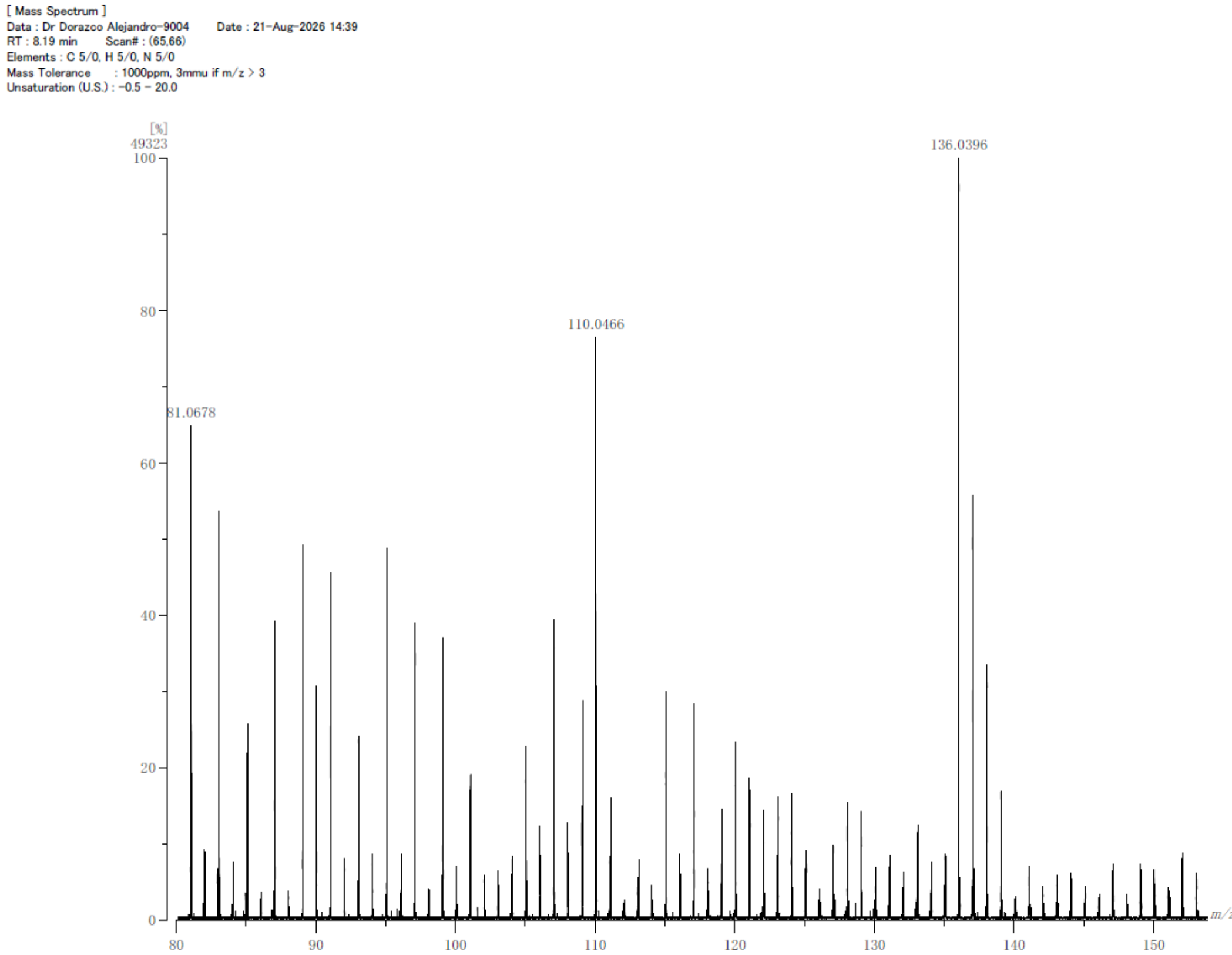

Fig. S3 HRMS-EI(+) experiment of compound **1**.

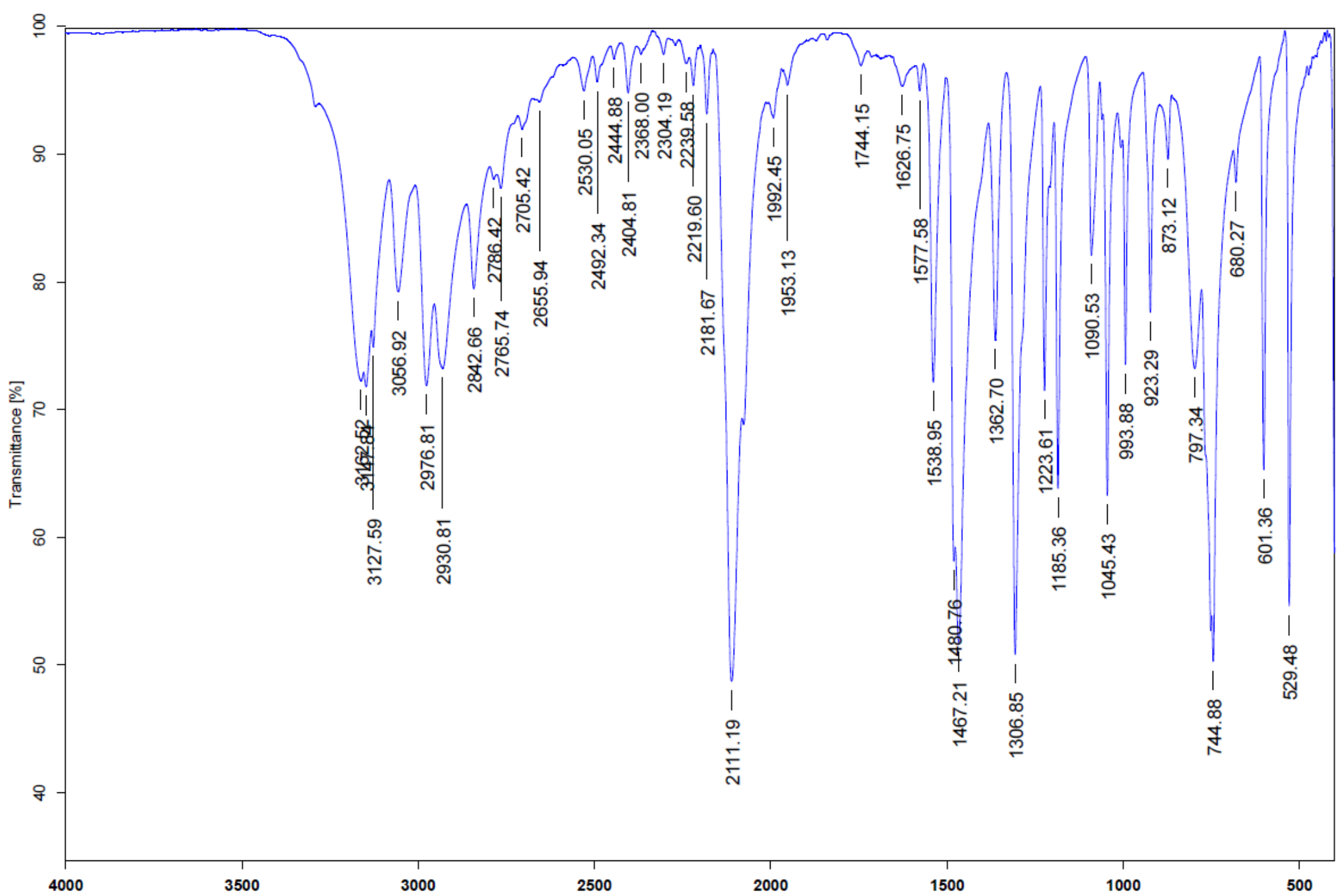

Fig. S4 FT-IR experiment of compound **1**.

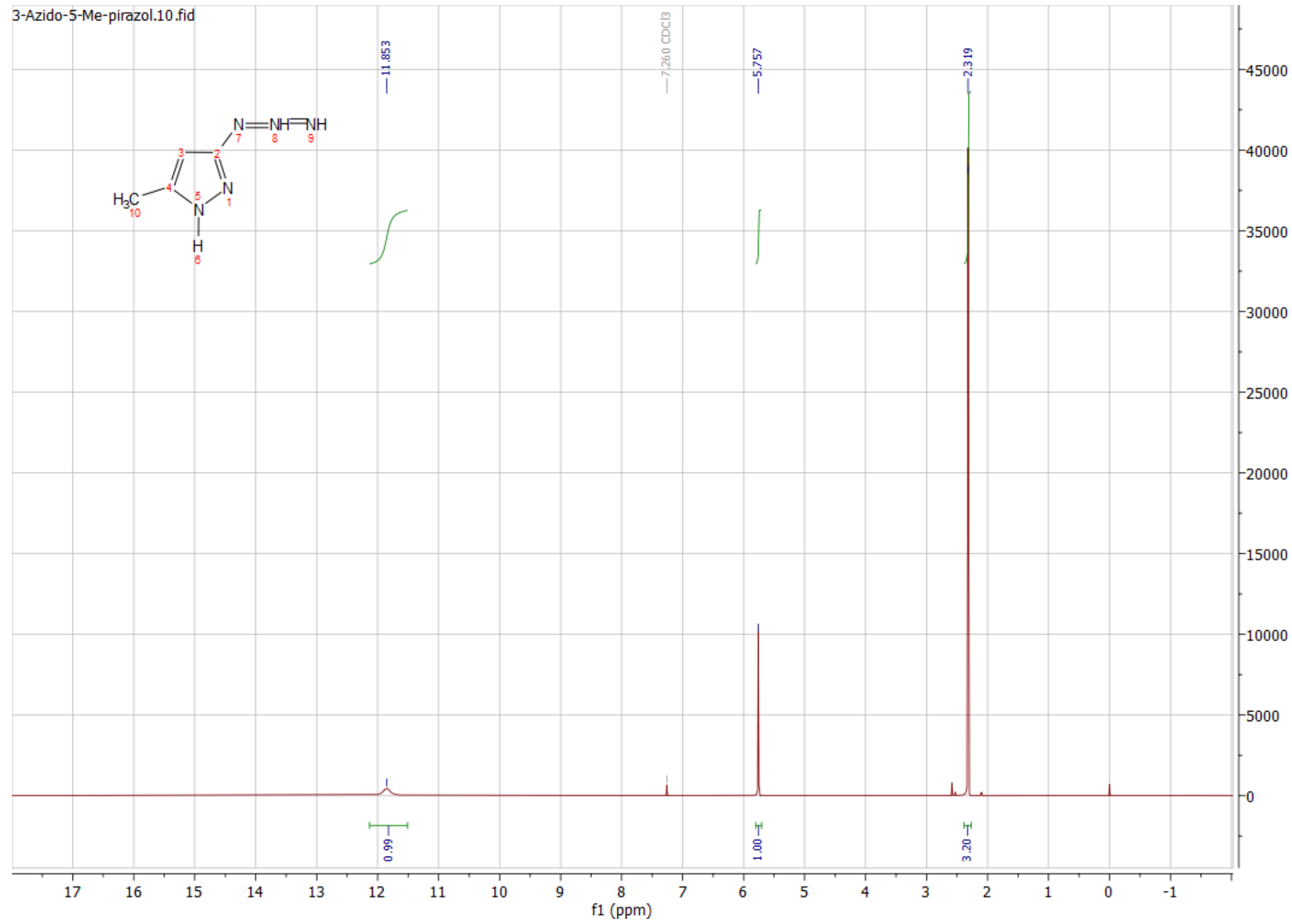

Fig. S5 ^{1}H-NMR of compound **2** in $CDCl_3$ at 25 °C.

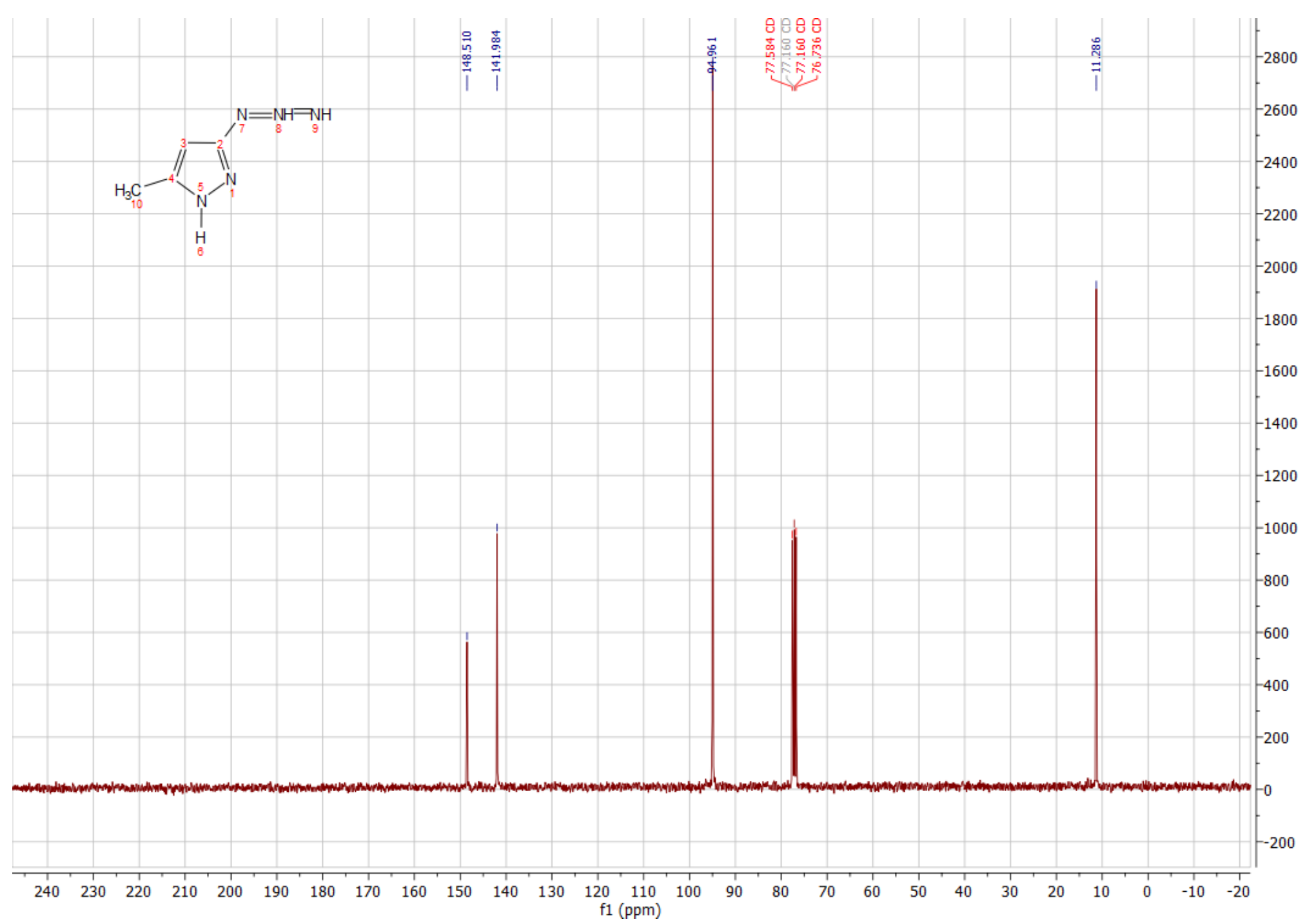

Fig. S6 ^{13}C-NMR of compound **2** in $CDCl_3$ at 25 °C.

INSTITUTO DE QUIMICA, UNAM
LABORATORIO DE ESPECTROMETRIA DE MASAS

Data:46123 VAN-1
Sample Name:Dr Dorazco Alejandro / Operador: Carmen Garcia
Description:
Ionization Mode:ESI+
History:Determine m/z[Peak Detect[Centroid,30,Area];Correct Base[5.0%]];Correct Base[5.0%];Average(MS[1] 3..3)

Acquired:8/17/2026 10:28:17 AM
Operator:AccuTOF
Mass Calibration data:CAL_PEG_600_26-
Created:8/17/2026 10:40:43 AM
Created by:AccuTOF

Charge number:1
Element:^{12}C:0 .. 10, ^{1}H:0 .. 10, ^{14}N:1 .. 5

Tolerance:3.00(ppm), 5.00 .. 15.00(mmu)

Unsaturation Number:-2.0 .. 50.0 (Fraction:Both)

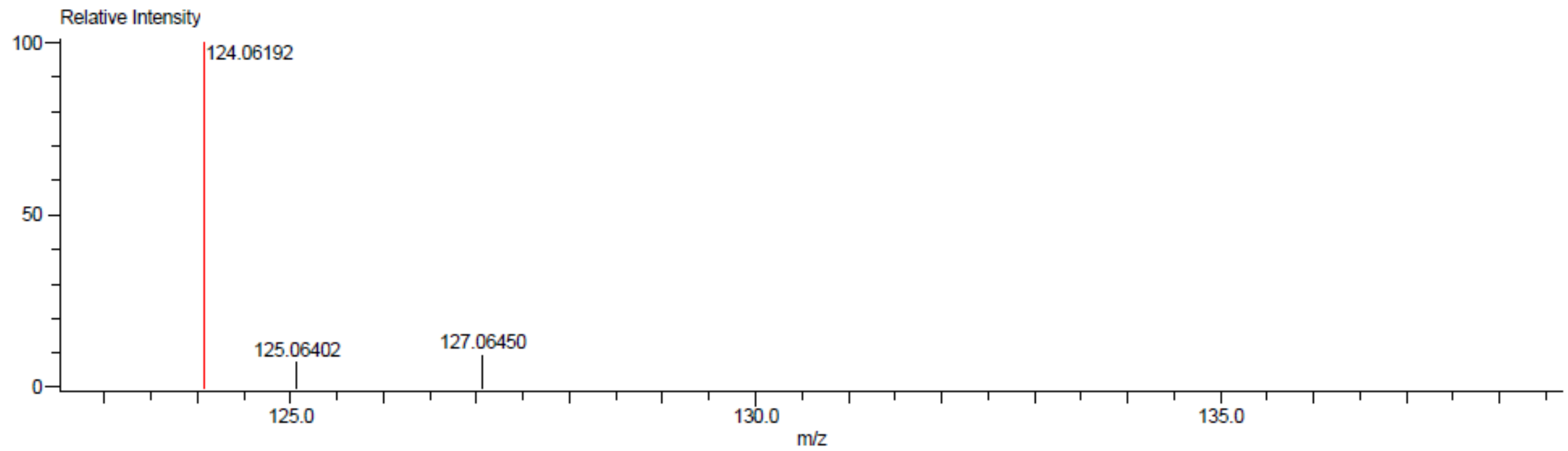

Mass	Intensity	Calc. Mass	Mass Difference (mmu)	Mass Difference (ppm)	Possible Formula	Unsaturation Number
124.06192	234422.38	124.06232	-0.40	-3.19	$^{12}C_4{}^{1}H_6{}^{14}N_5$	4.5

Fig. S7 HRMS-DART(+) experiment of compound **2**.

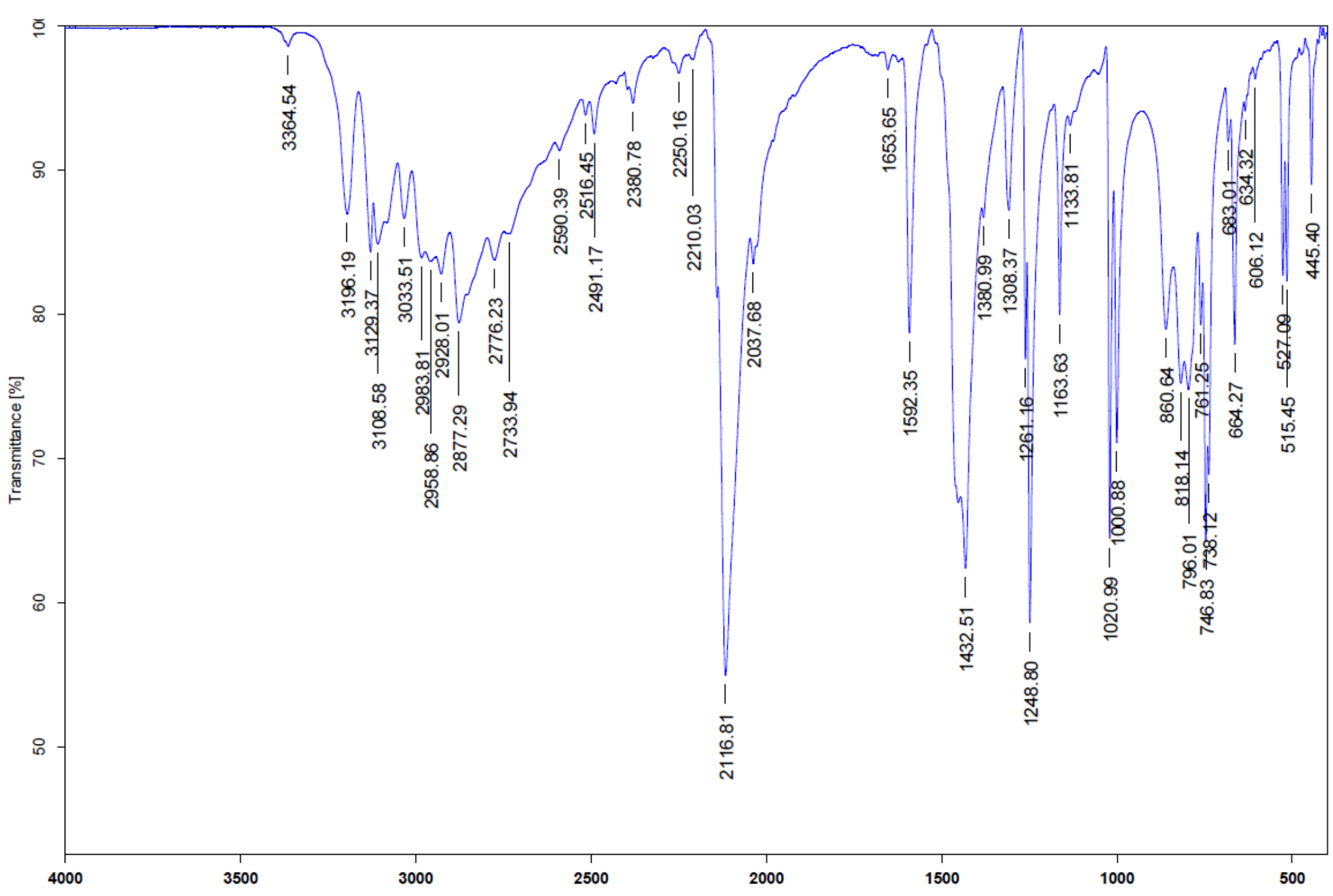

Fig. S8 FT-IR experiment of compound **2**.

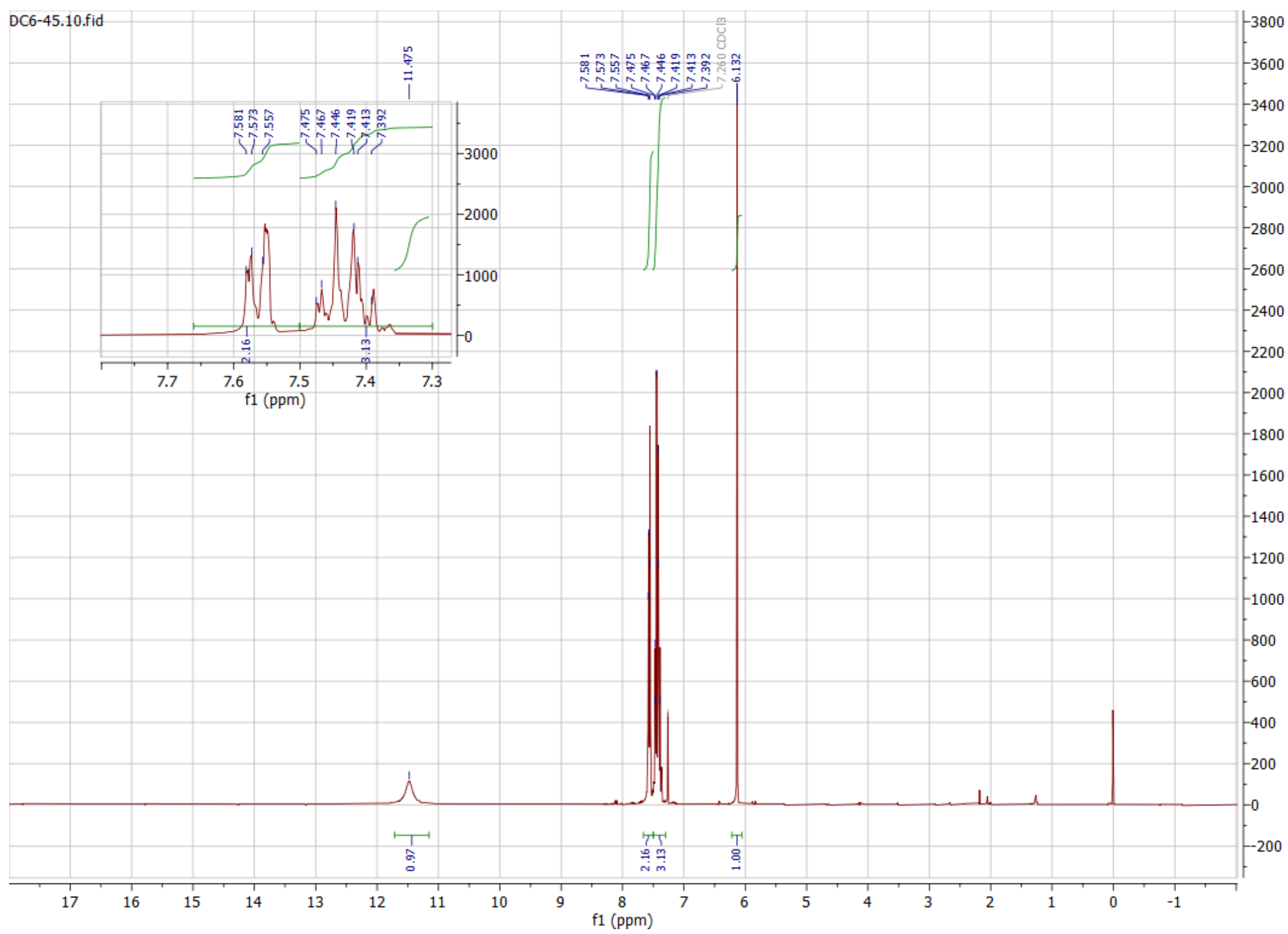

Fig. S9 ^{1}H-NMR of compound **3** in $CDCl_3$ at 25 °C.

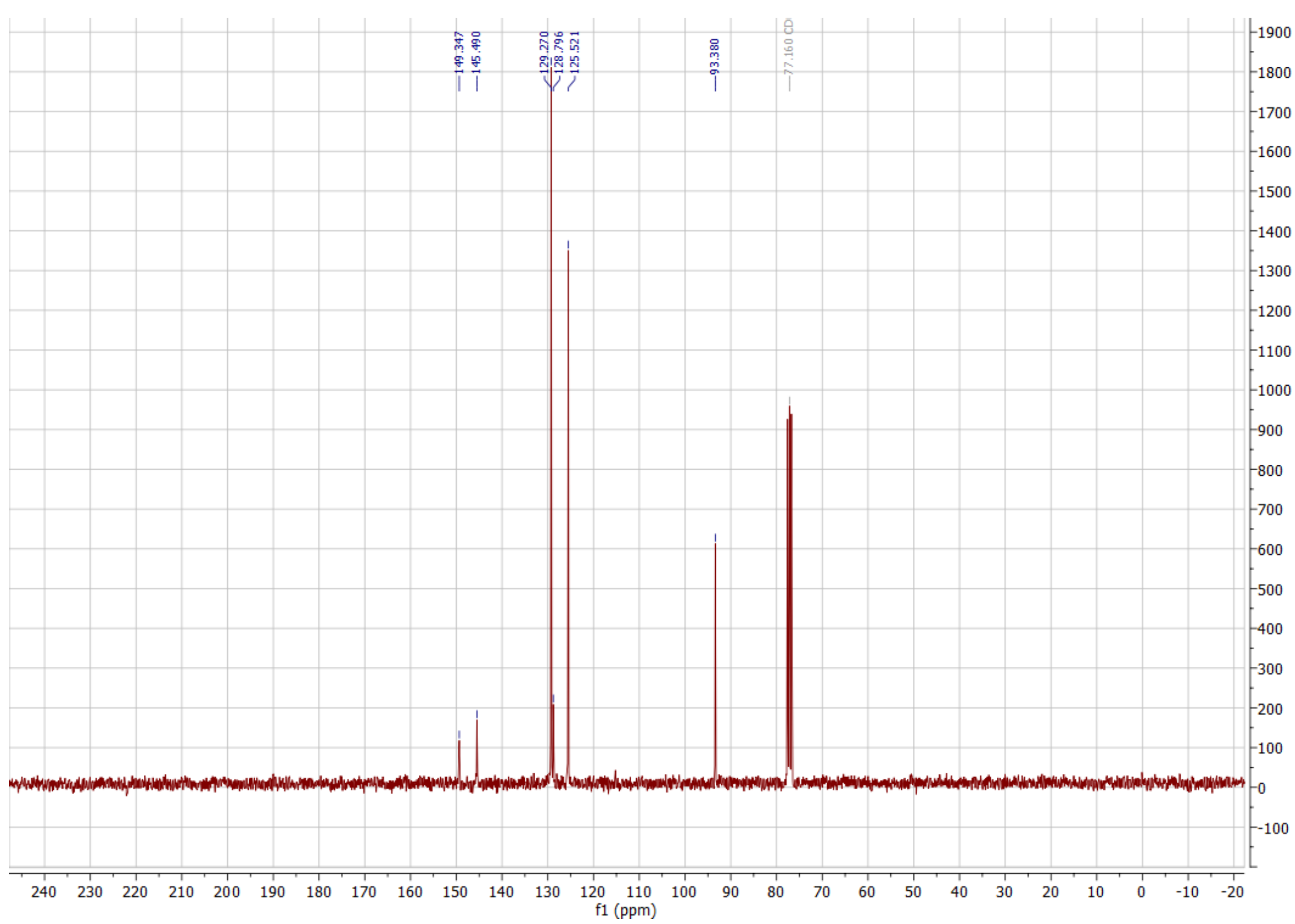

Fig. S10 ^{13}C-NMR of compound **3** in $CDCl_3$ at 25 °C**.**

INSTITUTO DE QUIMICA, UNAM
LABORATORIO DE ESPECTROMETRIA DE MASAS

Data:27_MAA-2
Sample Name:Dr. Dorazco Alejandro / Operador: Carmen Garcia
Description:
Ionization Mode:ESI+
History:Determine m/z[Peak Detect[Centroid,30,Area];Correct Base[5.0%]];Correct Base[5.0%];Average(MS[1] 1..1)

Acquired:1/11/2024 2:51:43 PM
Operator:AccuTOF
Mass Calibration data:CAL_PEG_600_26-
Created:8/17/2026 8:38:41 AM
Created by:AccuTOF

Charge number:1
Element:^{12}C:0 .. 10, ^{1}H:0 .. 10, ^{14}N:1 .. 5

Tolerance:3.00(ppm), 5.00 .. 15.00(mmu)

Unsaturation Number:-2.0 .. 50.0 (Fraction:Both)

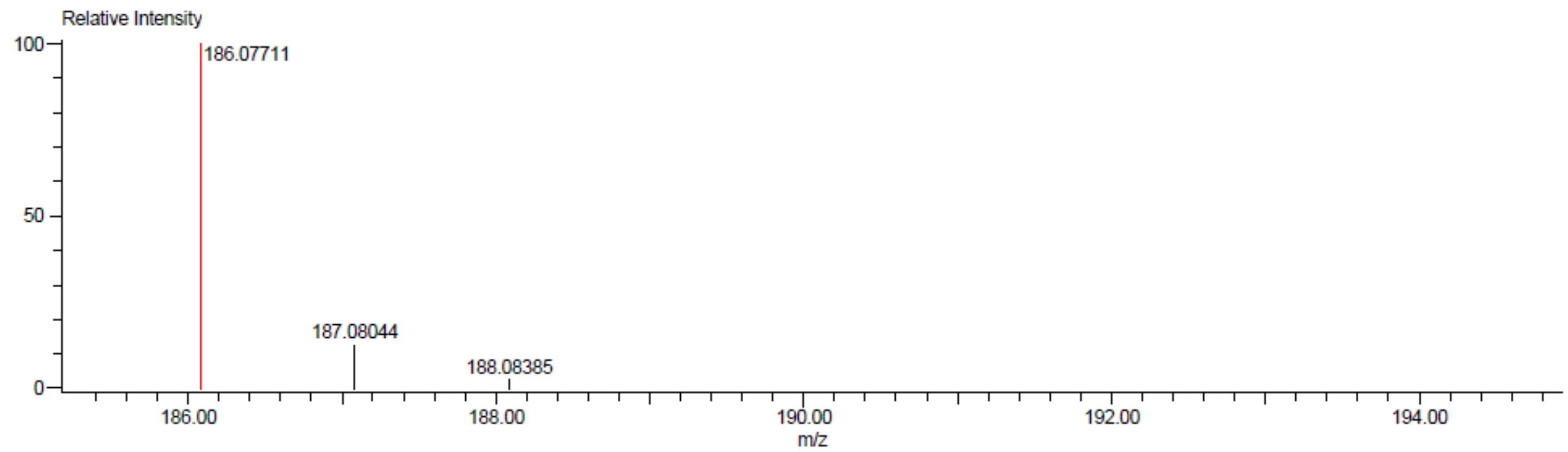

Mass	Intensity	Calc. Mass	Mass Difference (mmu)	Mass Difference (ppm)	Possible Formula	Unsaturation Number
186.07711	395932.50	186.07797	-0.86	-4.63	$^{12}C_9{}^{1}H_8{}^{14}N_5$	8.5

Fig. S11 HRMS-DART(+) experiment of compound **3**.

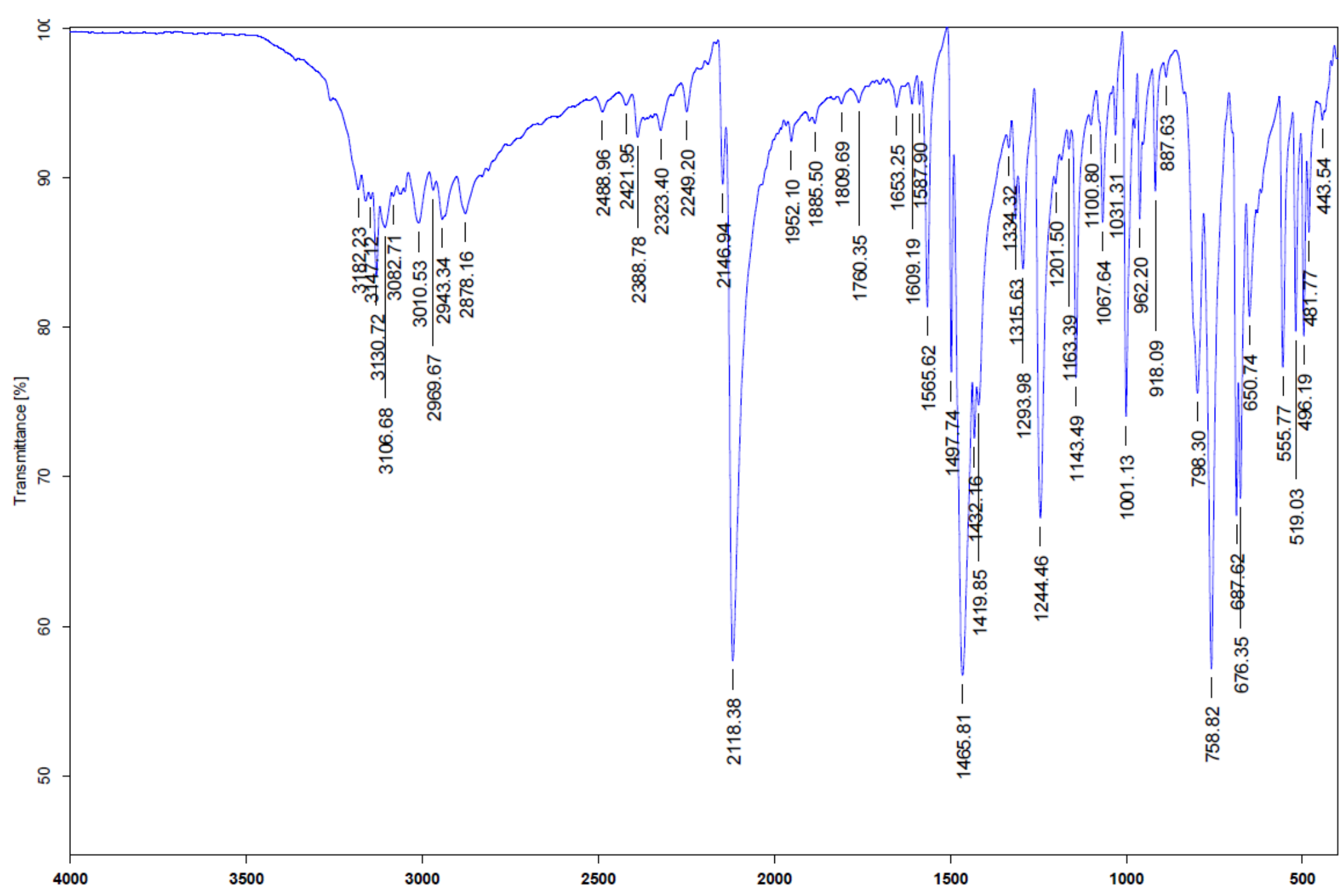

Fig. S12 FT-IR experiment of compound **3**.

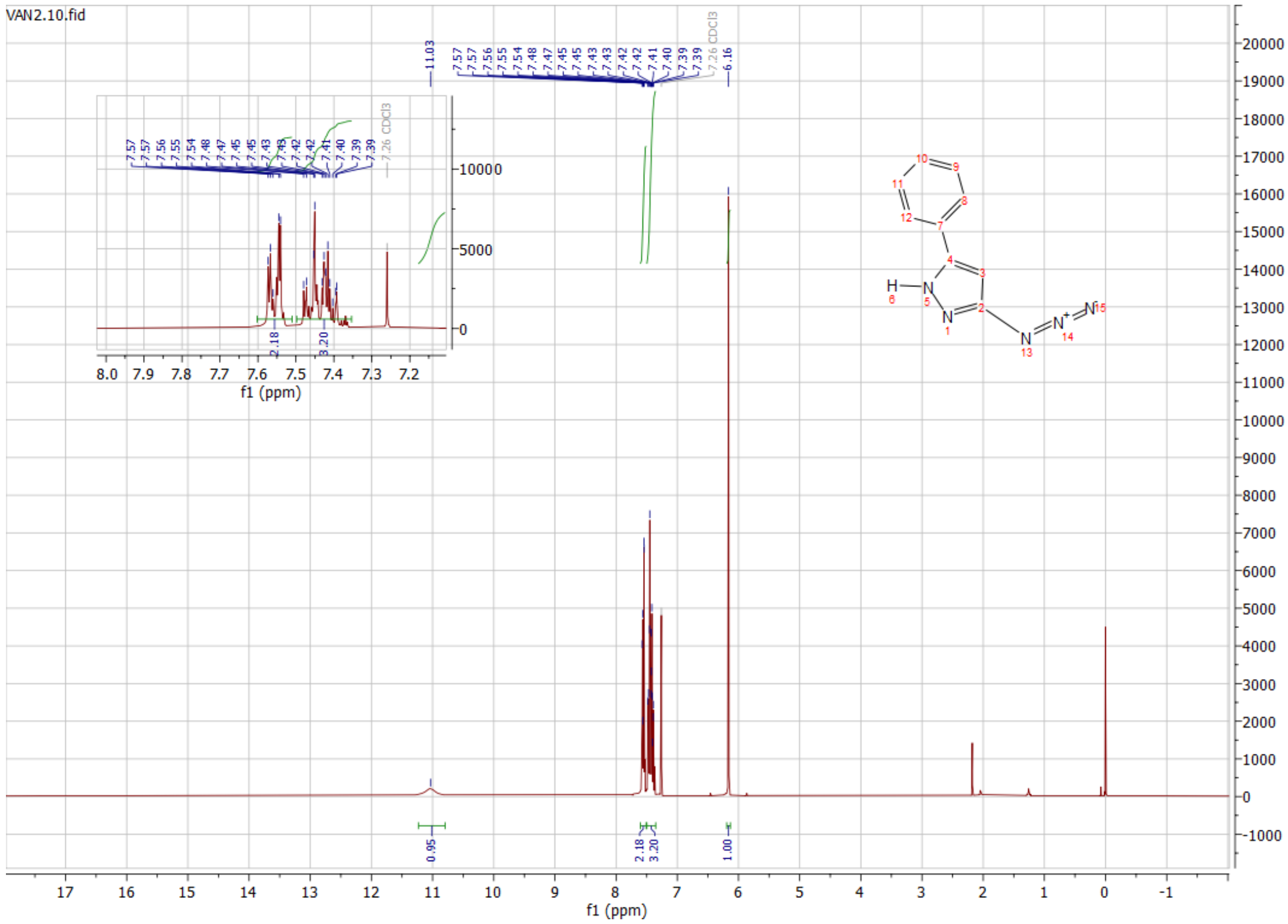

Fig. S13 ^{1}H-NMR of compound **4** in $CDCl_3$ at 25 °C.

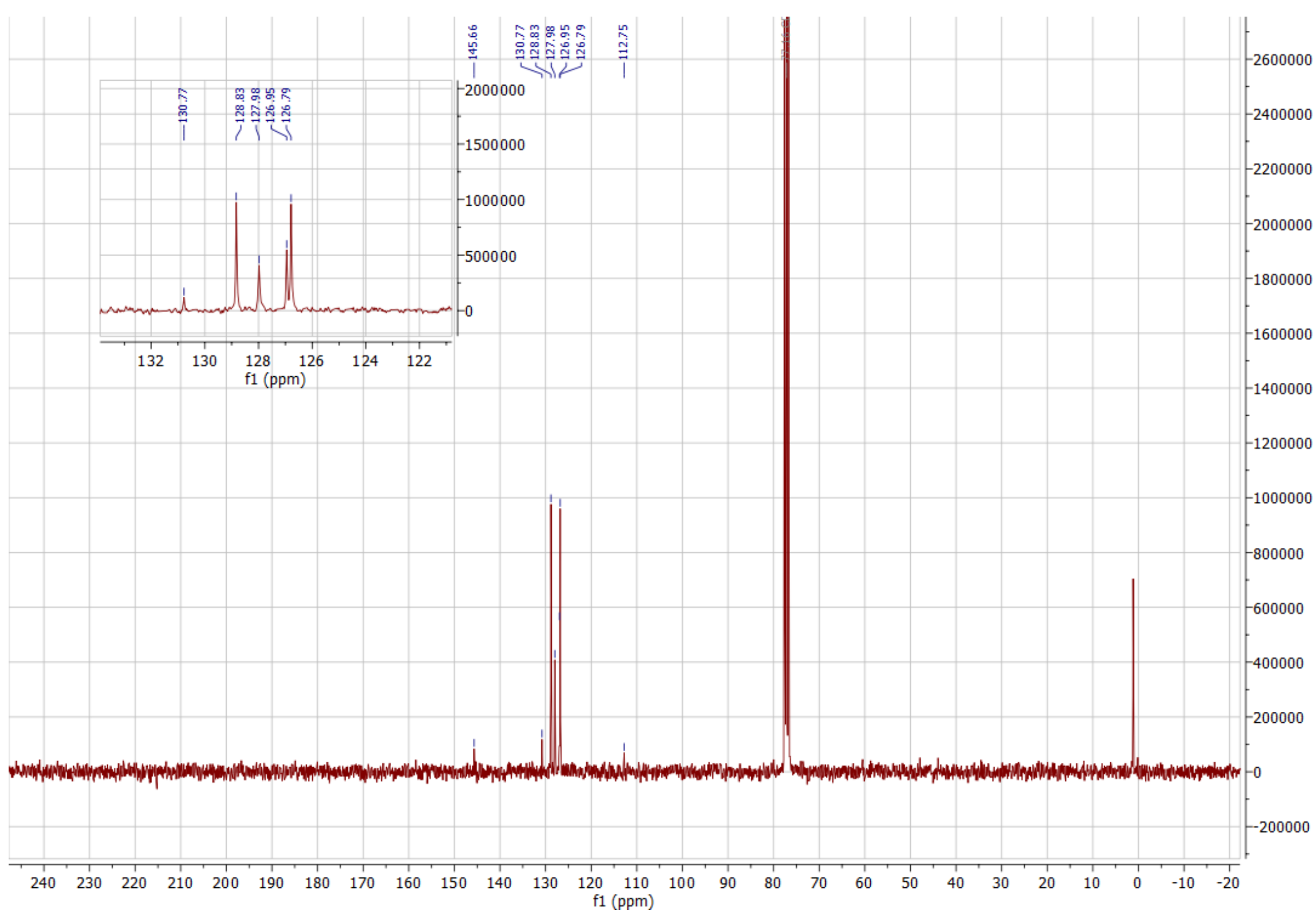

Fig. S14 ^{13}C-NMR of compound **4** in $CDCl_3$ at 25 °C.

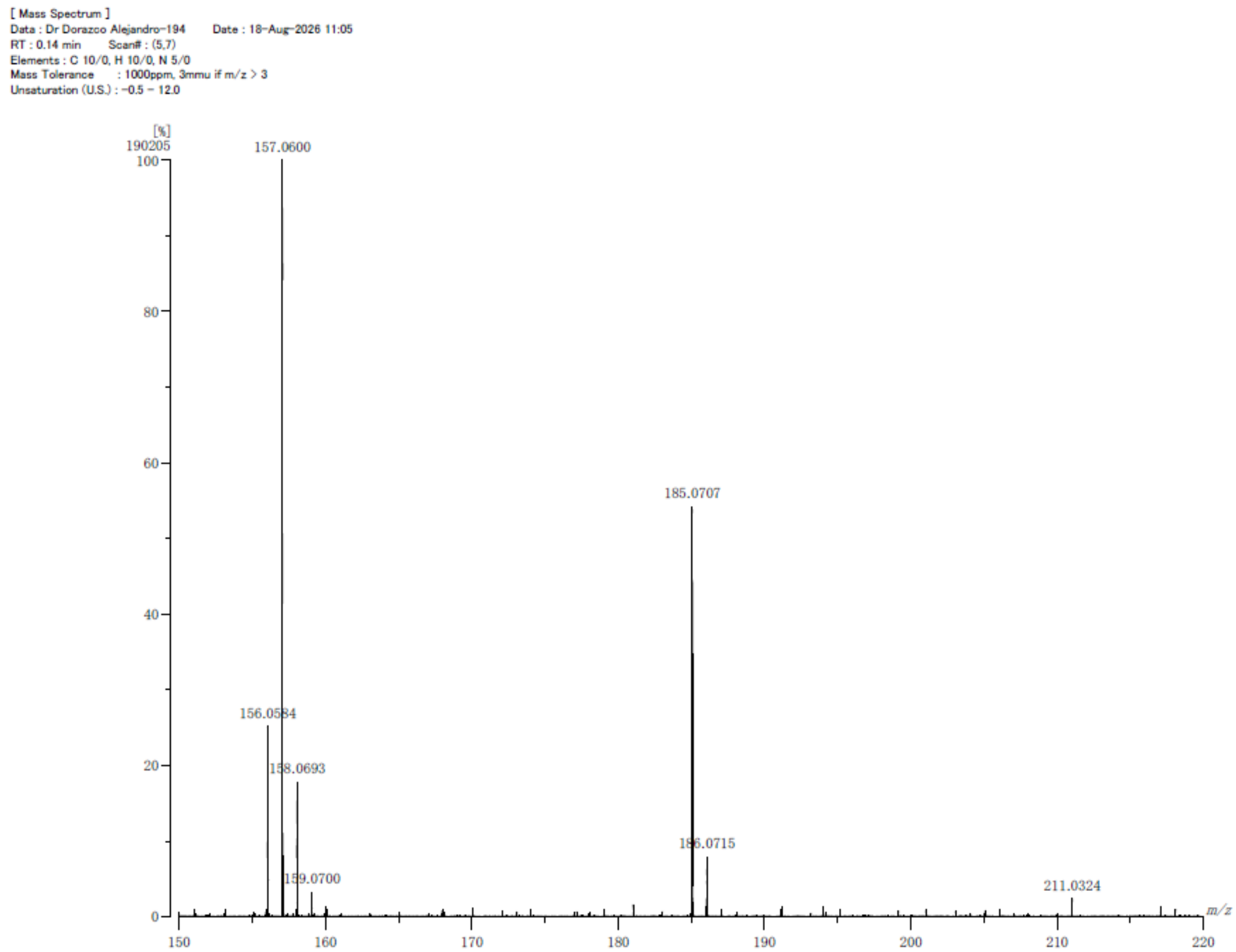

Fig. S15 HRMS-DART(+) experiment of compound **4**.

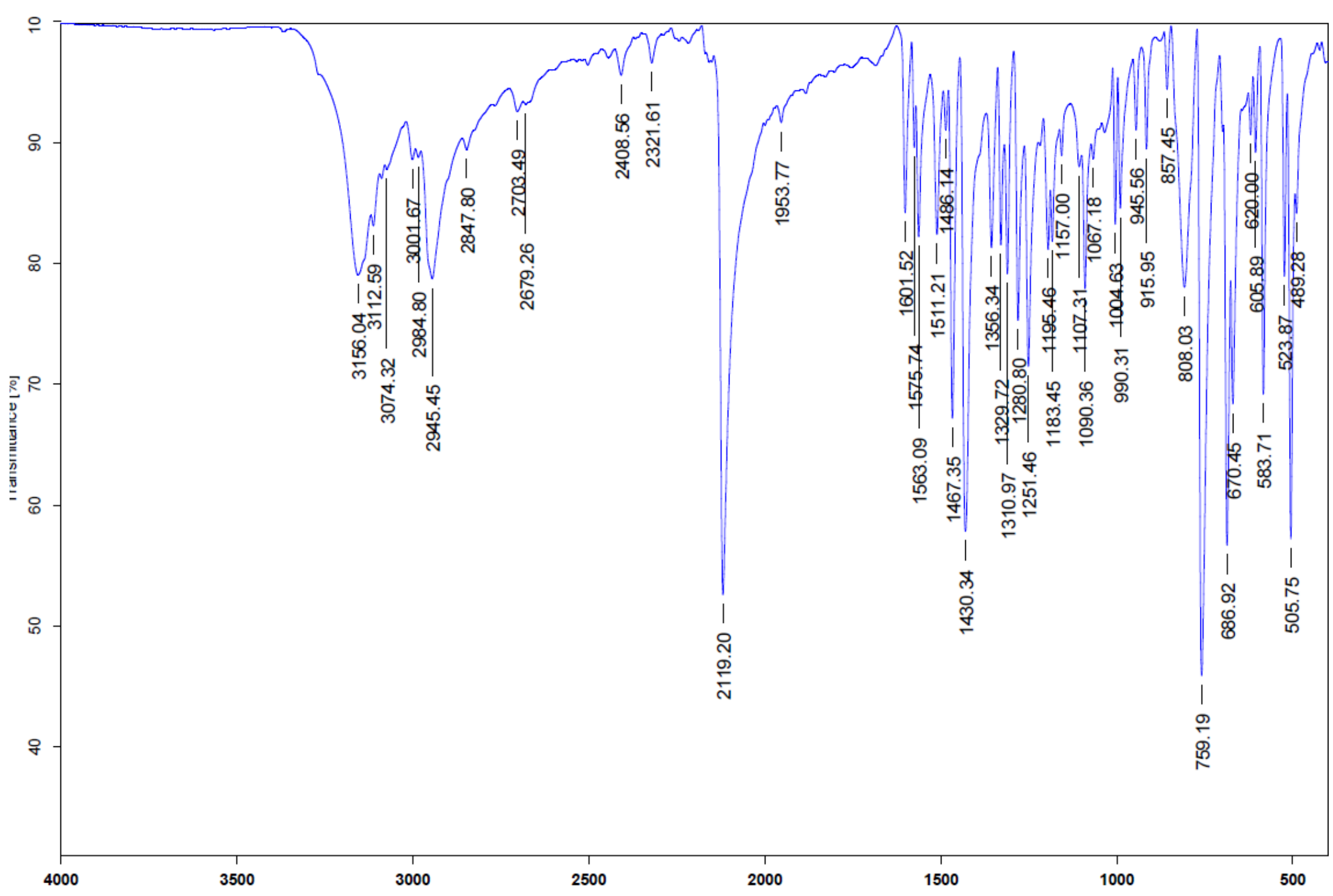

Fig. S16 FT-IR experiment of compound **4**.

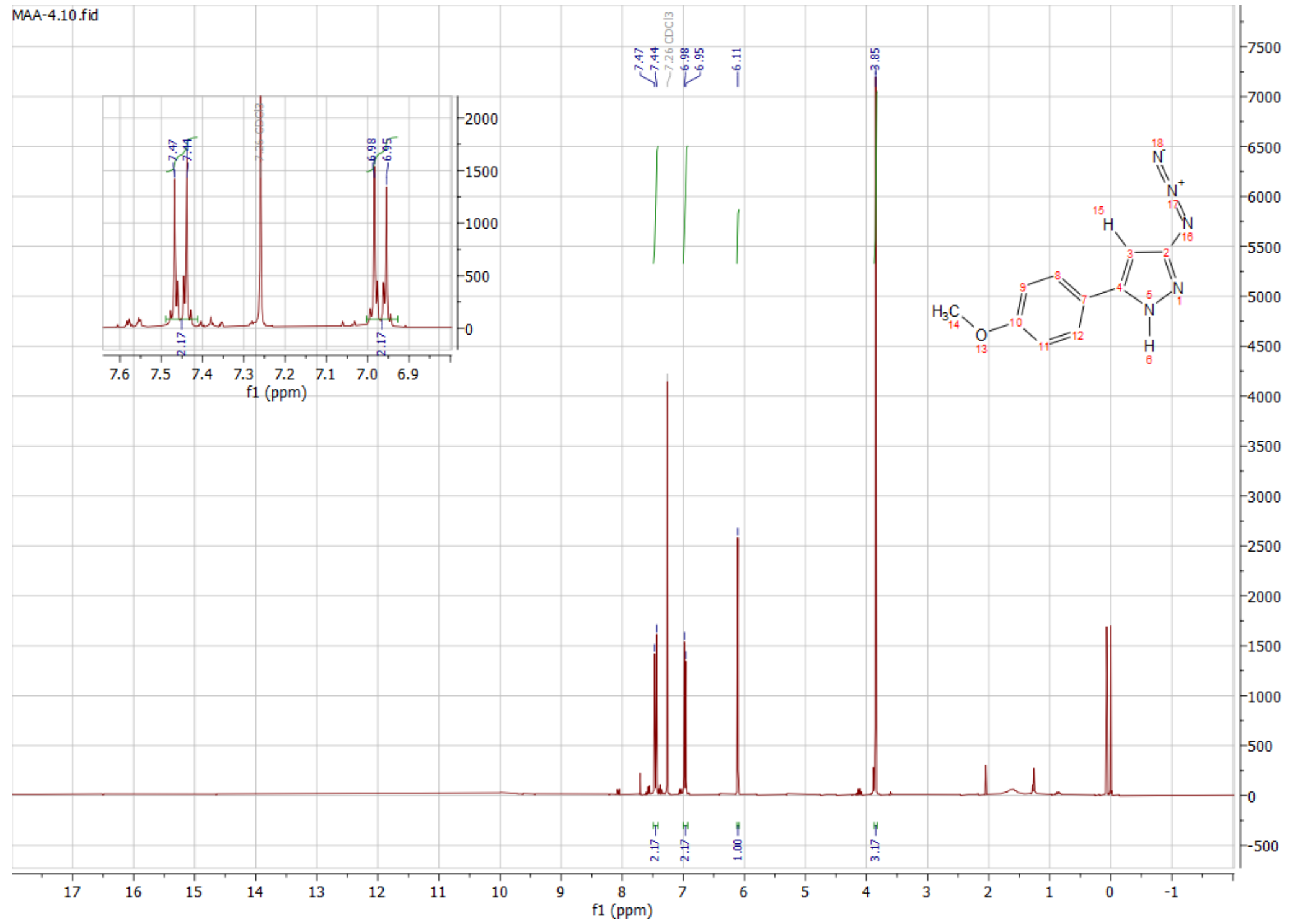

Fig. S17 ^{1}H-NMR of compound **5** in $CDCl_3$ at 25 °C.

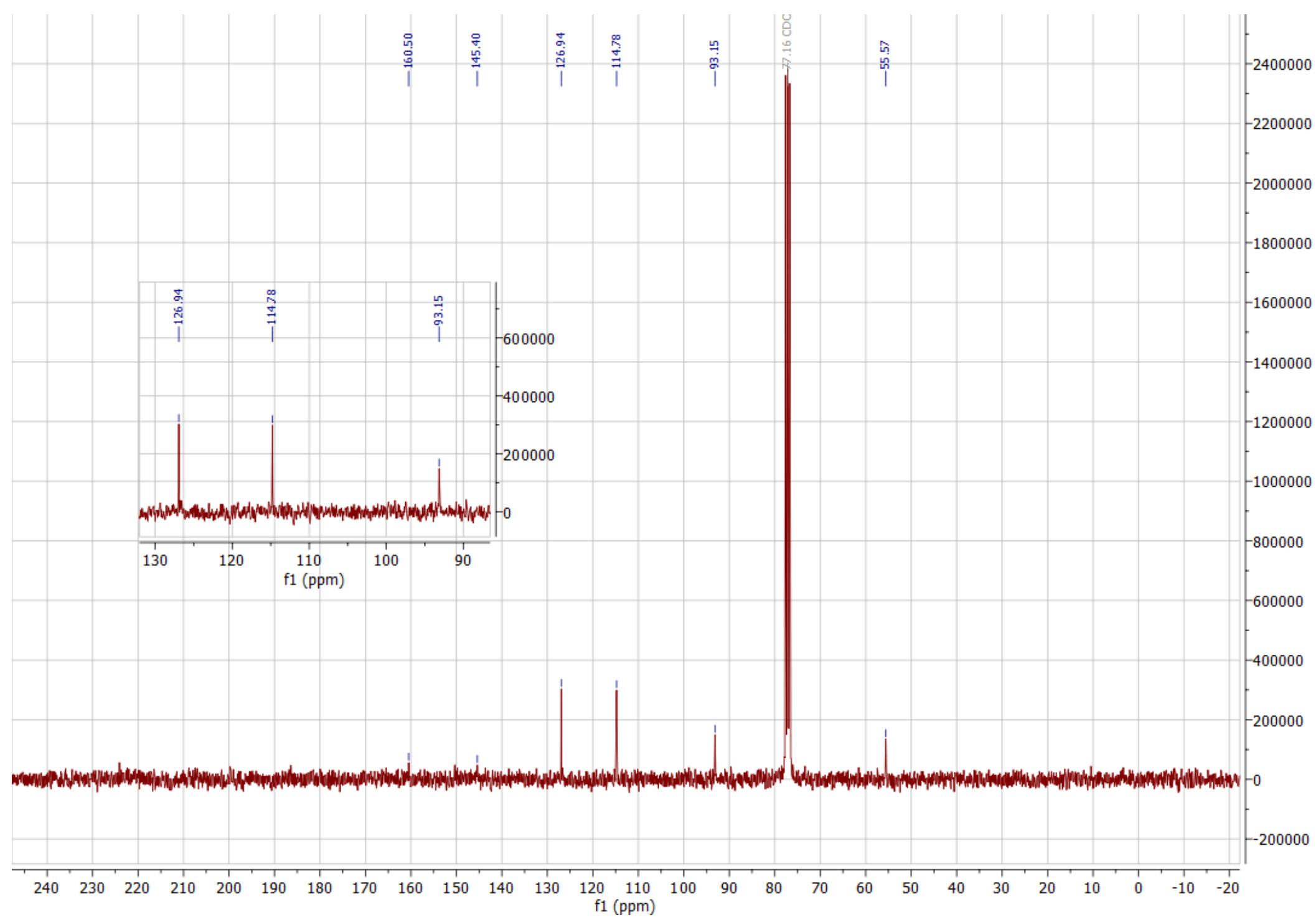

Fig. S18 ^{13}C-NMR of compound **5** in $CDCl_3$ at 25 °C.

INSTITUTO DE QUIMICA, UNAM
LABORATORIO DE ESPECTROMETRIA DE MASAS

Data:46127_MAA-4
Sample Name:Dr Dorazco Alejandro / Operador: Carmen Garcia
Description:
Ionization Mode:ESI+
History:Determine m/z[Peak Detect[Centroid,30,Area];Correct Base[5.0%]];Correct Base[5.0%];Average(MS[1] 1..1)

Acquired:8/14/2026 2:43:28 PM
Operator:AccuTOF
Mass Calibration data:CAL_PEG_600_26-
Created:8/14/2026 3:34:10 PM
Created by:AccuTOF

Charge number:1
Element:^{12}C:0 .. 10, ^{1}H:0 .. 10, ^{14}N:1 .. 5, ^{16}O:1 .. 1

Tolerance:3.00(ppm), 5.00 .. 15.00(mmu)

Unsaturation Number:-2.0 .. 50.0 (Fraction:Both)

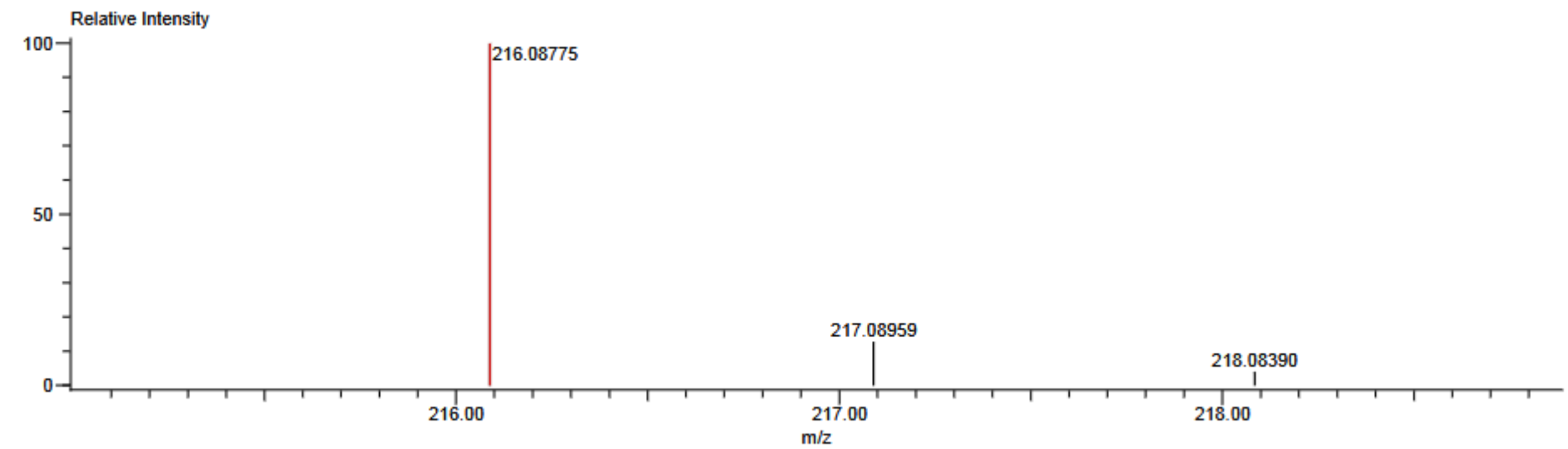

Mass	Intensity	Calc. Mass	Mass Difference (mmu)	Mass Difference (ppm)	Possible Formula	Unsaturation Number
216.08775	621734.35	216.08853	-0.79	-3.65	$^{12}C_{10}{}^{1}H_{10}{}^{14}N_{5}{}^{16}O_{1}$	8.5

Fig. S19 HRMS-DART(+) experiment of compound **5**.

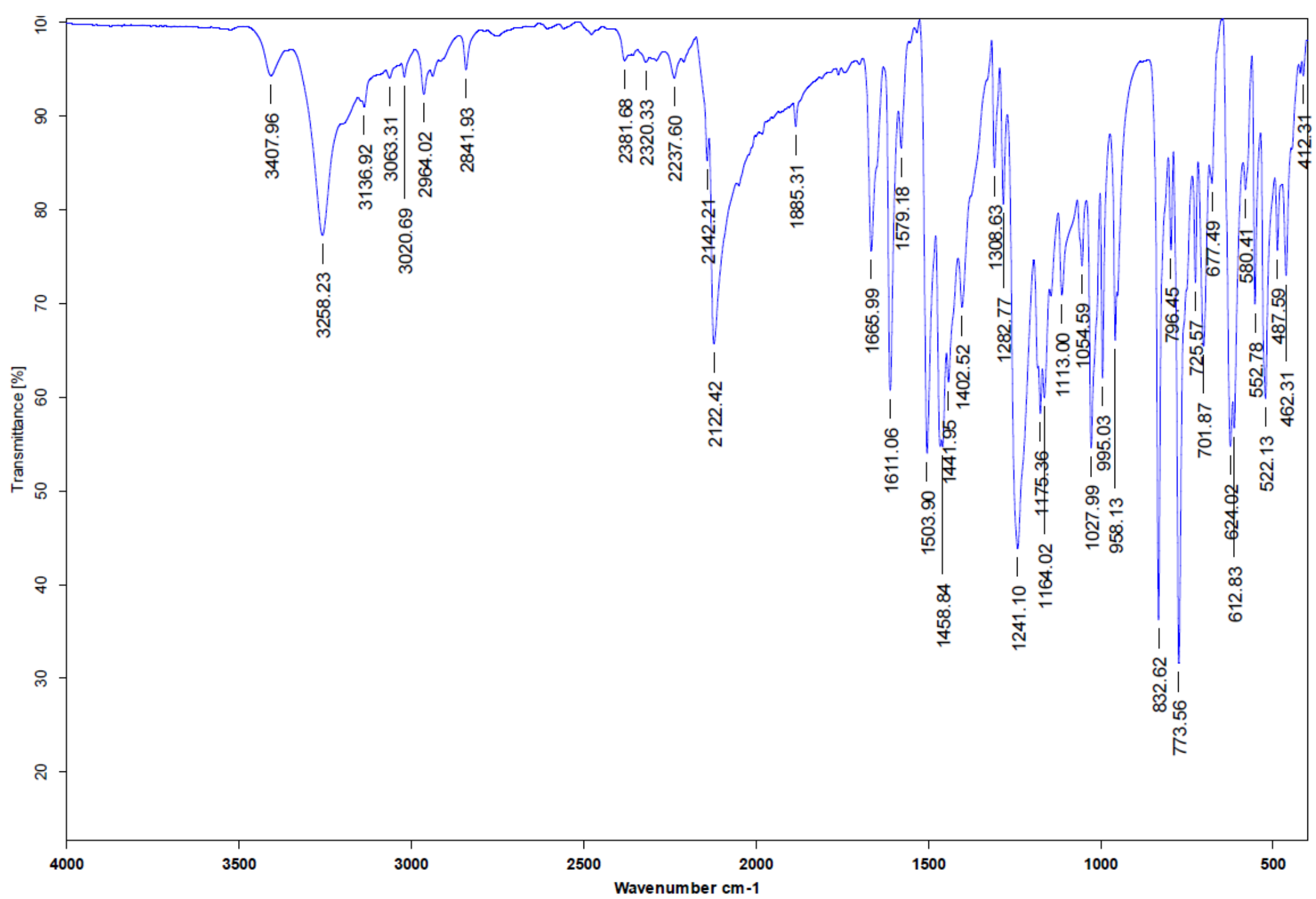

Fig. S20 FT-IR experiment of compound **5**.

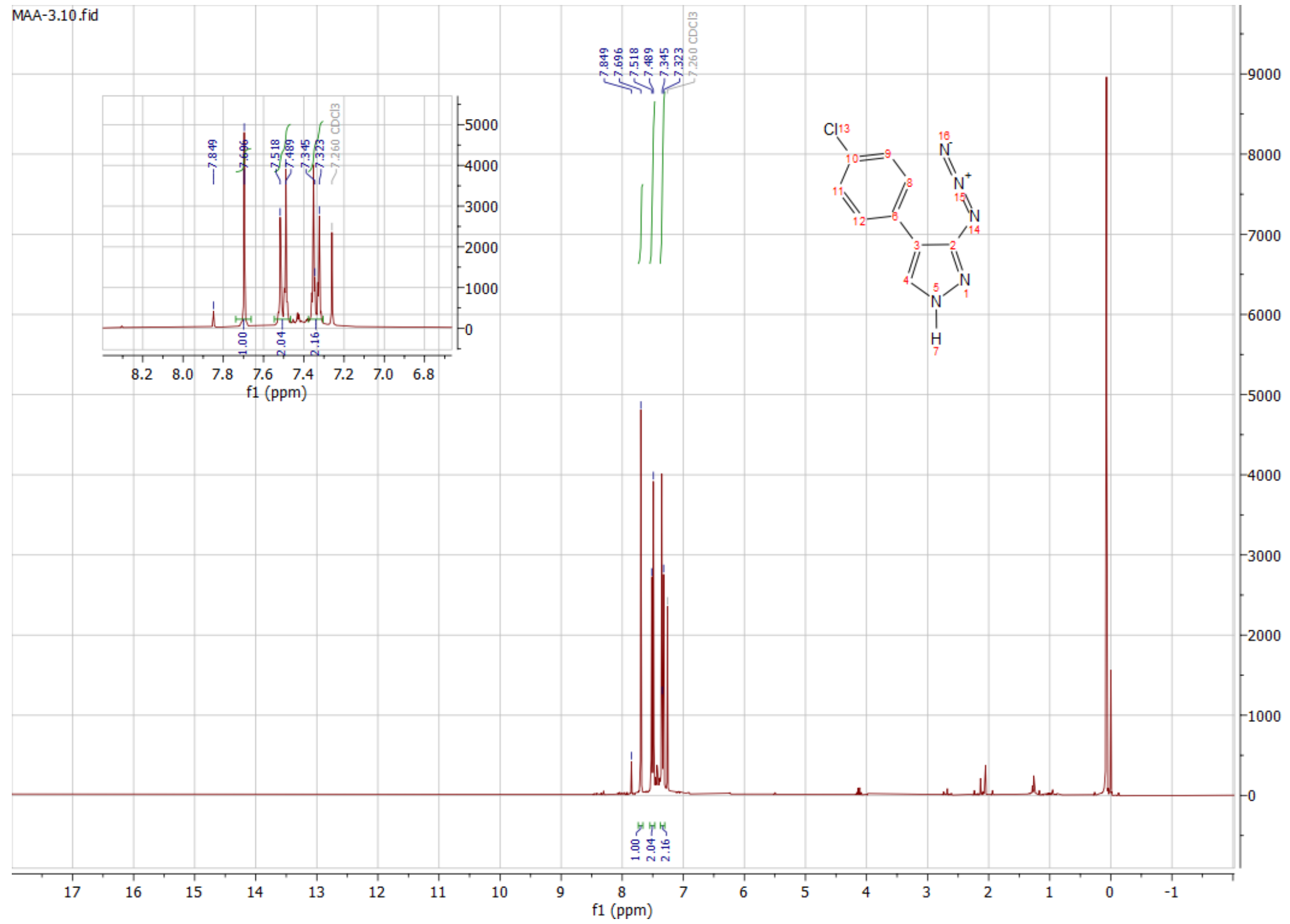

Fig. S21 ^{1}H-NMR of compound **6** in $CDCl_3$ at 25 °C.

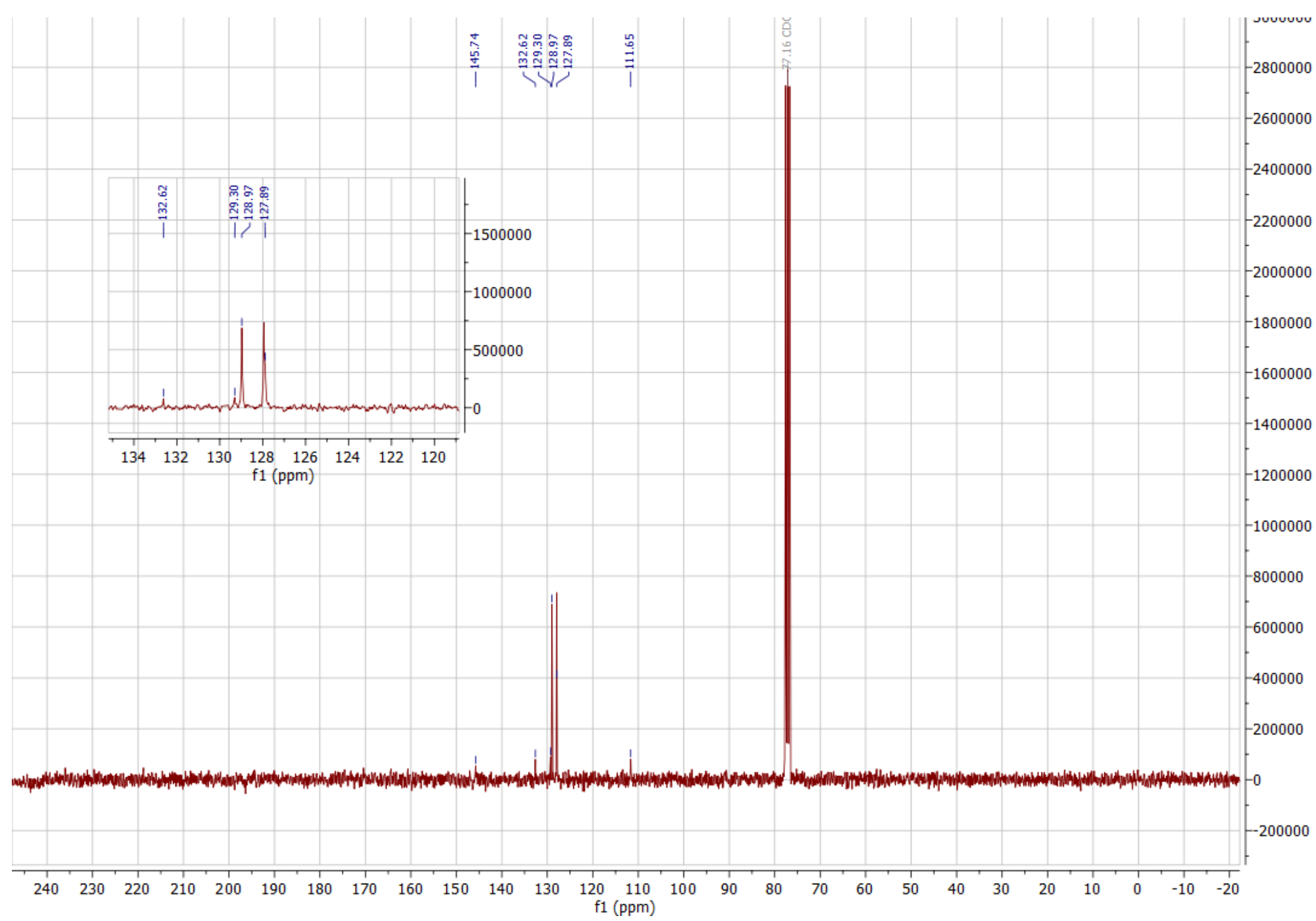

Fig. S22 ^{13}C-NMR of compound **6** in $CDCl_3$ at 25 °C.

INSTITUTO DE QUIMICA, UNAM
LABORATORIO DE ESPECTROMETRIA DE MASAS

Data:46128_MAA-3
Sample Name:Dr Dorazco Alejandro / Operador: Carmen Garcia
Description:
Ionization Mode:ESI+
History:Determine m/z[Peak Detect[Centroid,30,Area];Correct Base[5.0%]];Correct Base[5.0%];Average(MS[1] 1..1)

Acquired:8/14/2026 2:47:27 PM
Operator:AccuTOF
Mass Calibration data:CAL_PEG_600_26-
Created:8/17/2026 8:06:11 AM
Created by:AccuTOF

Charge number:1
Element:^{12}C:0 .. 10, ^{1}H:0 .. 10, ^{35}Cl:1 .. 1, ^{14}N:1 .. 5

Tolerance:3.00(ppm), 5.00 .. 15.00(mmu)

Unsaturation Number:-2.0 .. 50.0 (Fraction:Both)

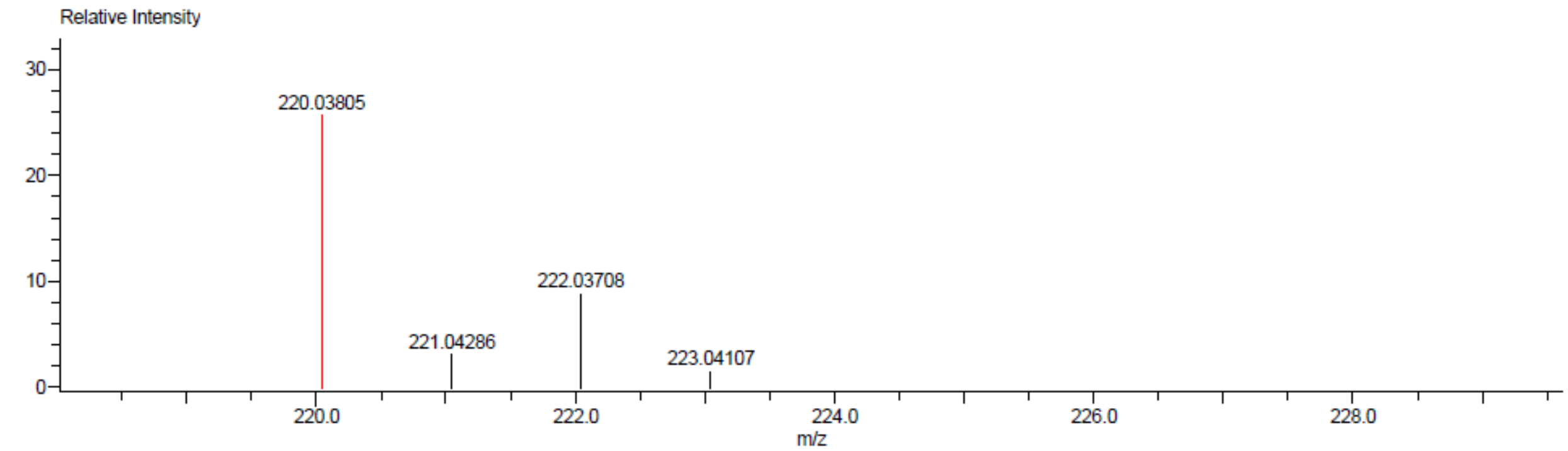

Mass	Intensity	Calc. Mass	Mass Difference (mmu)	Mass Difference (ppm)	Possible Formula	Unsaturation Number
220.03805	52794.00	220.03900	-0.95	-4.31	$^{12}C_9{}^{1}H_7{}^{35}Cl_1{}^{14}N_5$	8.5

Fig. S23 HRMS-DART(+) experiment of compound **6**.

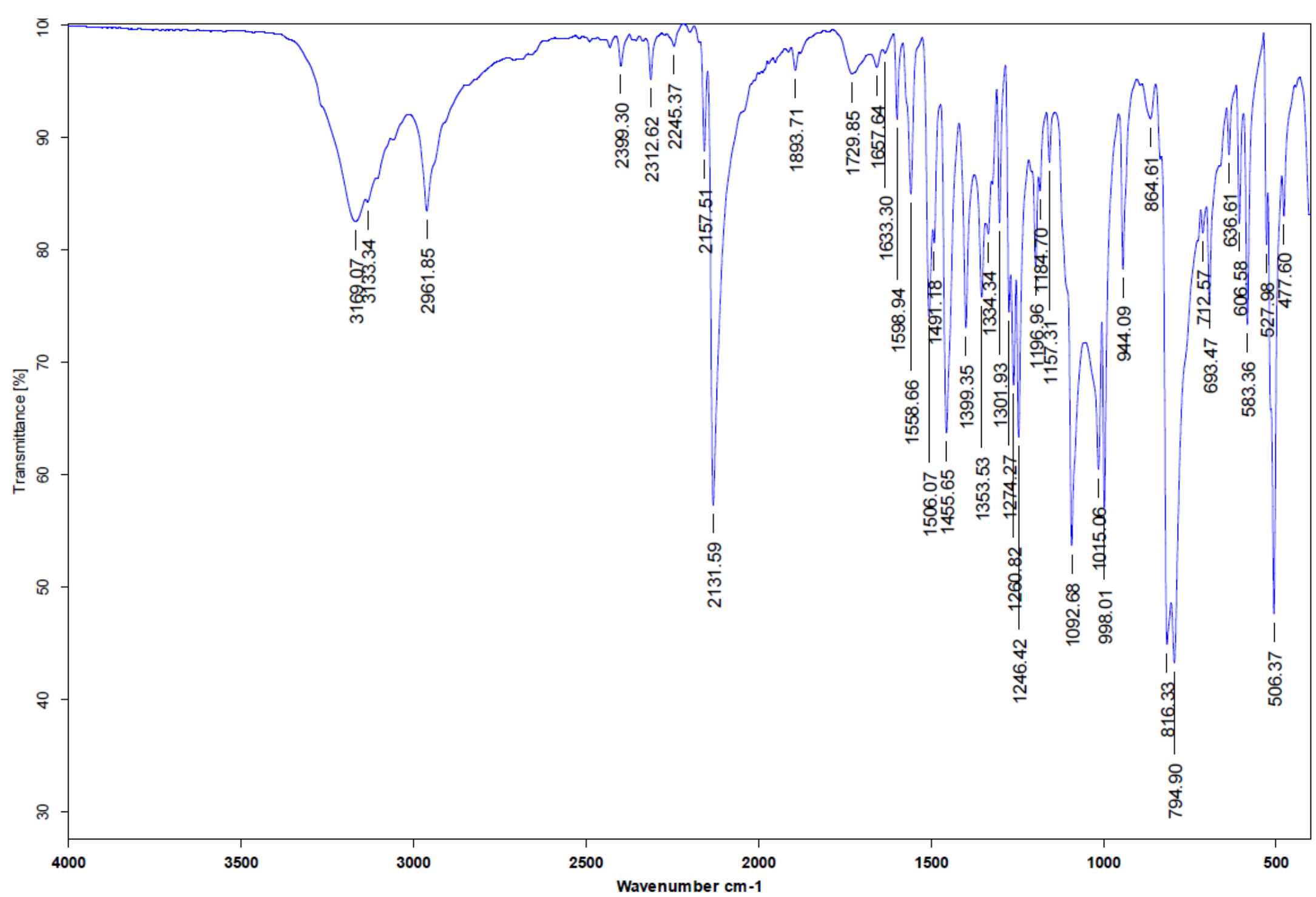

Fig. S24 FT-IR experiment of compound **6**.

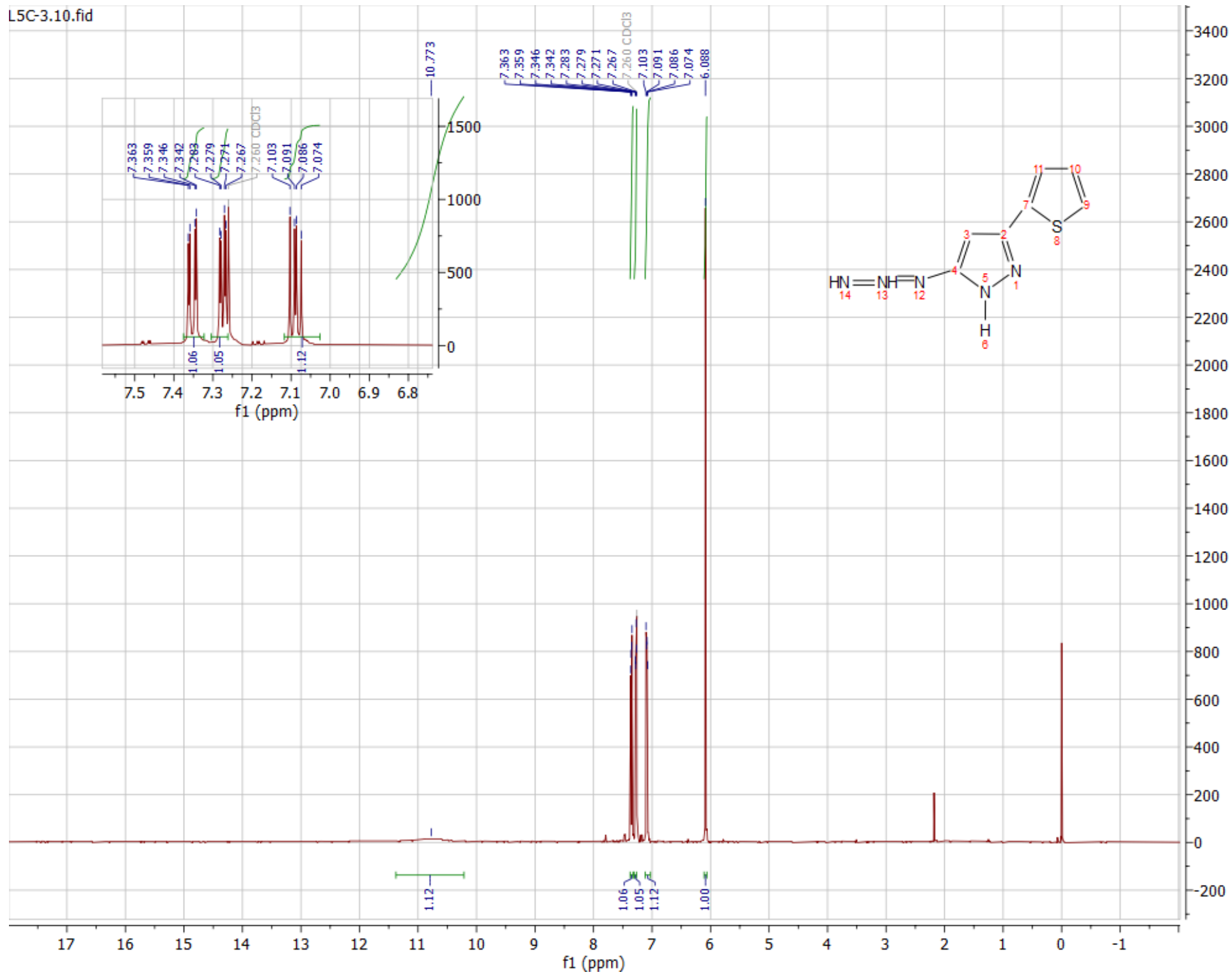

Fig. S25 ^{1}H-NMR of compound **7** in $CDCl_3$ at 25 °C.

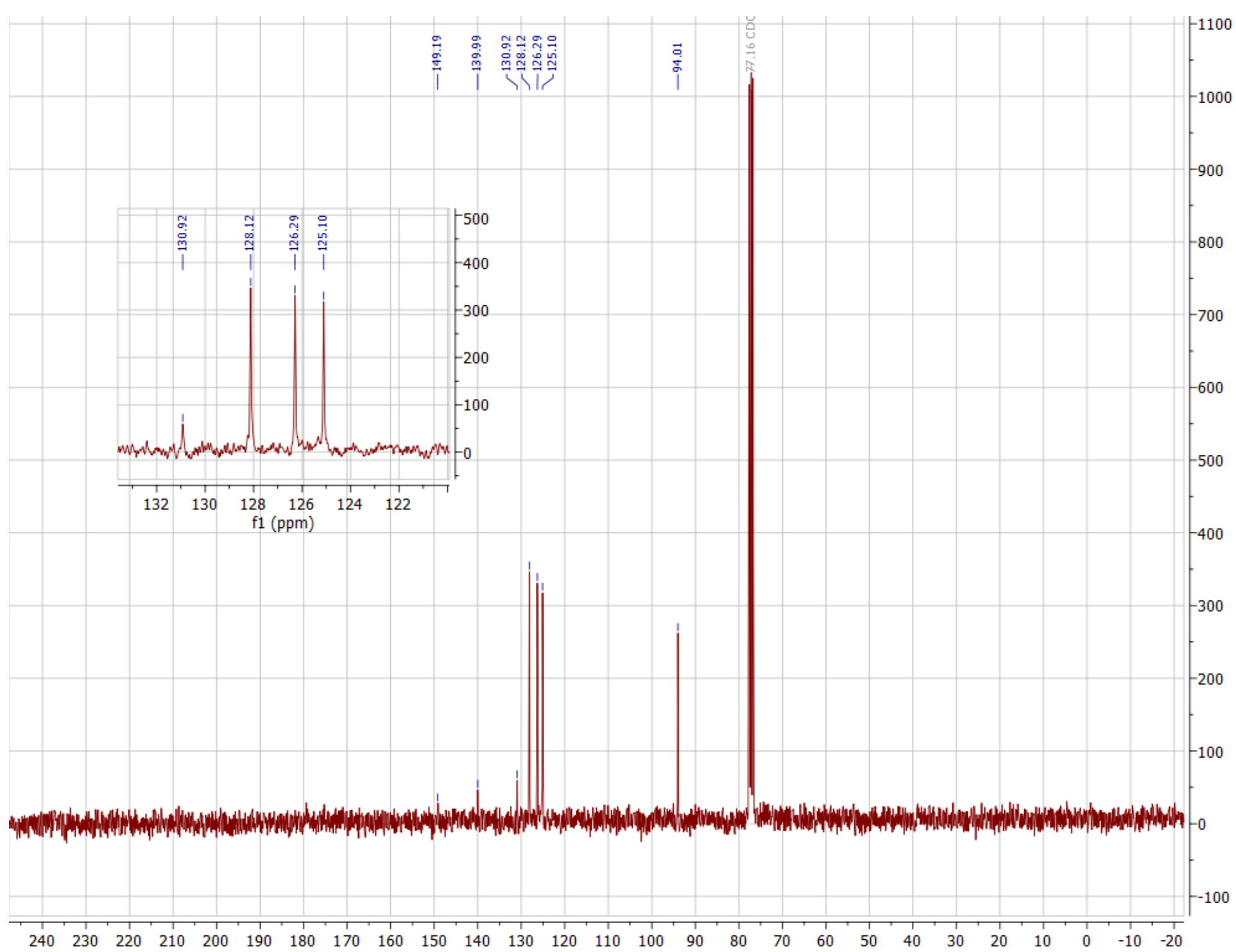

Fig. S26 ^{13}C-NMR of compound **7** in $CDCl_3$ at 25 °C.

INSTITUTO DE QUIMICA, UNAM
LABORATORIO DE ESPECTROMETRIA DE MASAS

Data:46129_KPAC-CBL-2
Sample Name:Dr Dorazco Alejandro / Operador: Carmen Garcia
Description:
Ionization Mode:ESI+
History:Determine m/z[Peak Detect[Centroid,30,Area];Correct Base[5.0%]];Correct Base[5.0%];Average(MS[1] 1..1)

Acquired:8/14/2026 2:50:41 PM
Operator:AccuTOF
Mass Calibration data:CAL_PEG_600_26-
Created:8/17/2026 8:22:06 AM
Created by:AccuTOF

Charge number:1
Element:^{12}C:0 .. 7, ^{1}H:0 .. 10, ^{14}N:1 .. 5, ^{32}S:0 .. 1

Tolerance:3.00(ppm), 5.00 .. 15.00(mmu)

Unsaturation Number:-2.0 .. 50.0 (Fraction:Both)

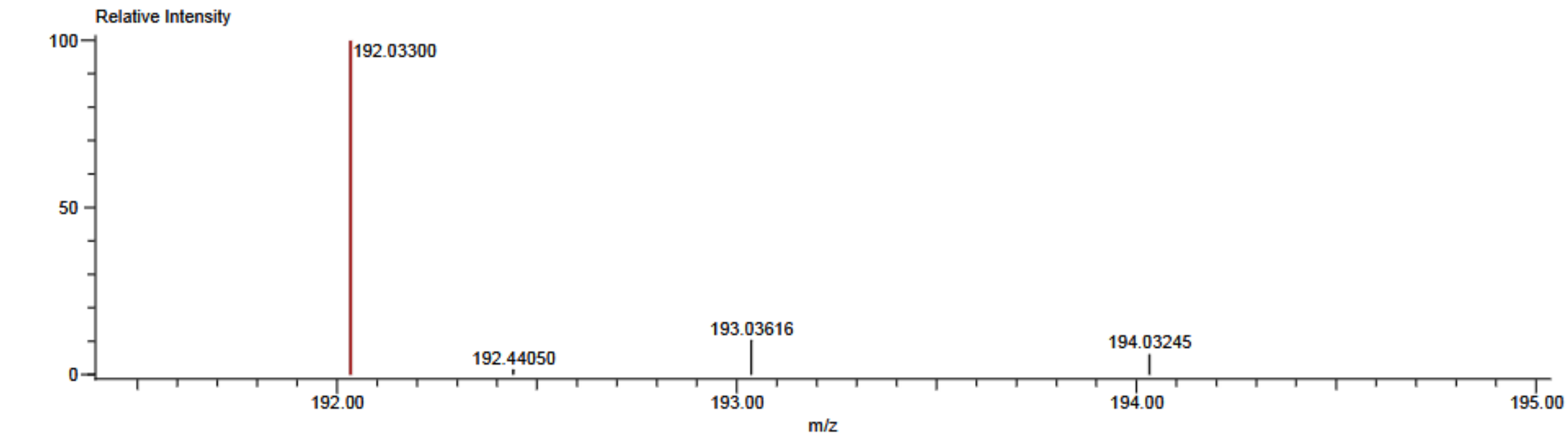

Mass	Intensity	Calc. Mass	Mass Difference (mmu)	Mass Difference (ppm)	Possible Formula	Unsaturation Number
192.03300	67118.25	192.03439	-1.39	-1.25	$^{12}C_7{}^{1}H_6{}^{14}N_5{}^{32}S_1$	7.5

Fig. S27 HRMS-DART(+) experiment of compound **7**.

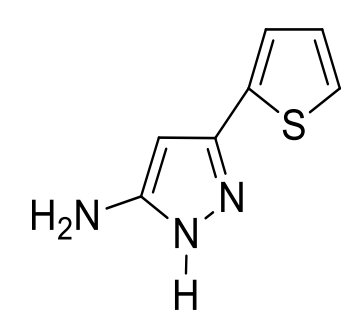

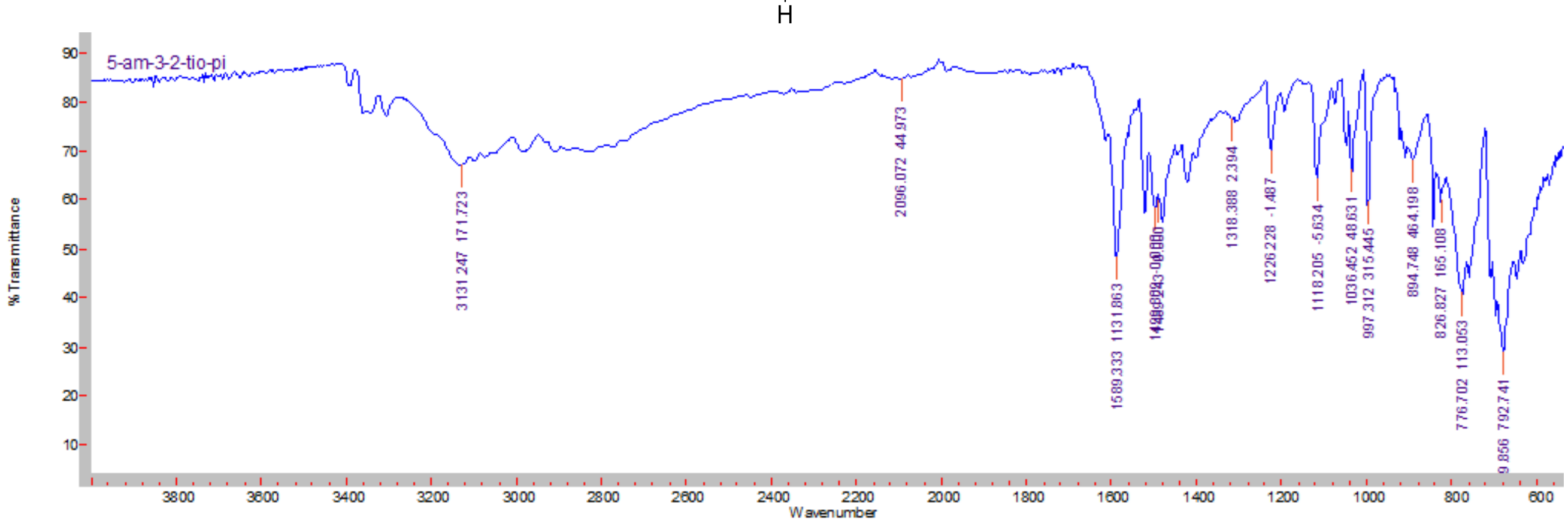

Fig. S28 FT-IR experiment of compound **7P**.

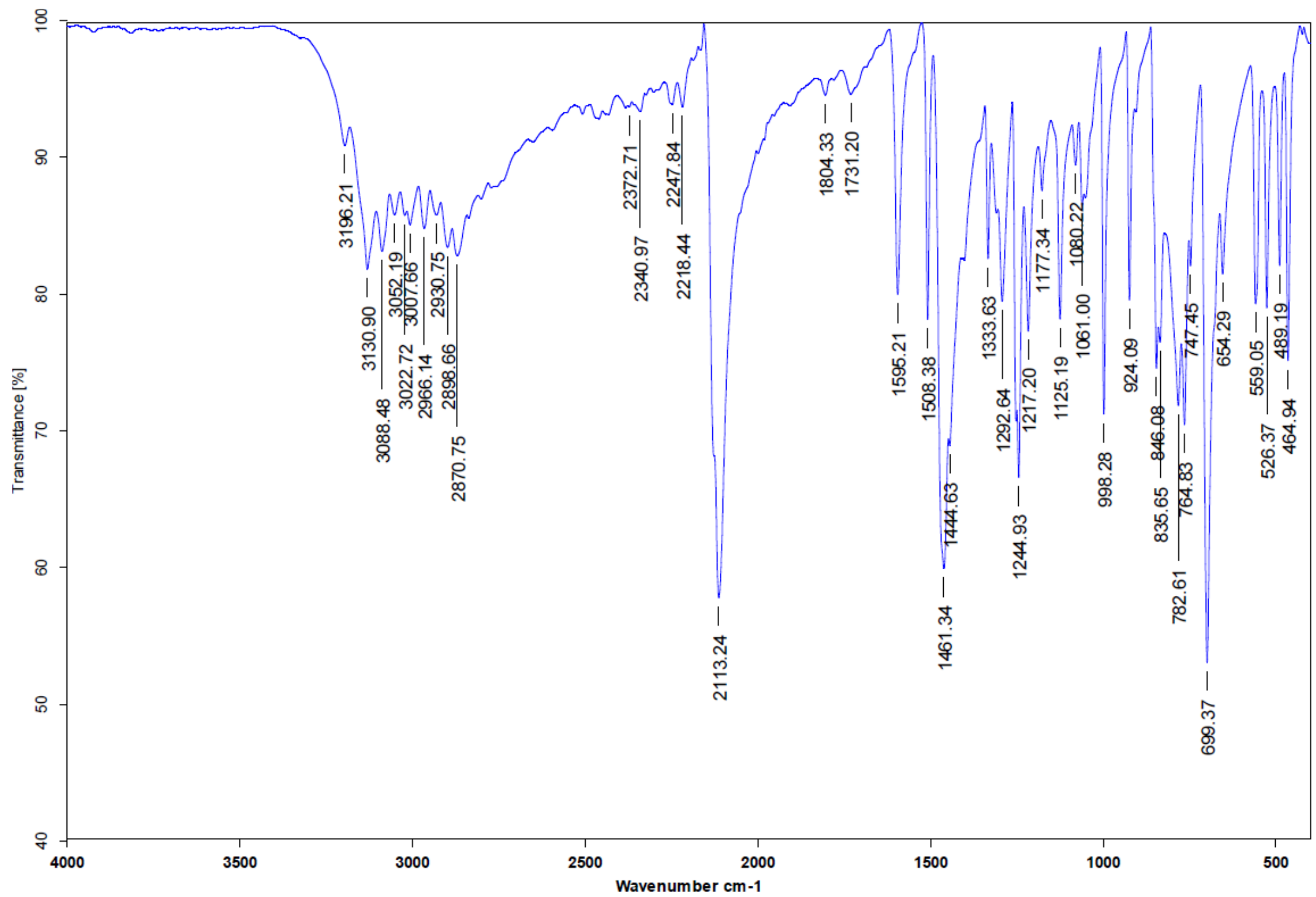

Fig. S29 FT-IR experiment of compound **7**.

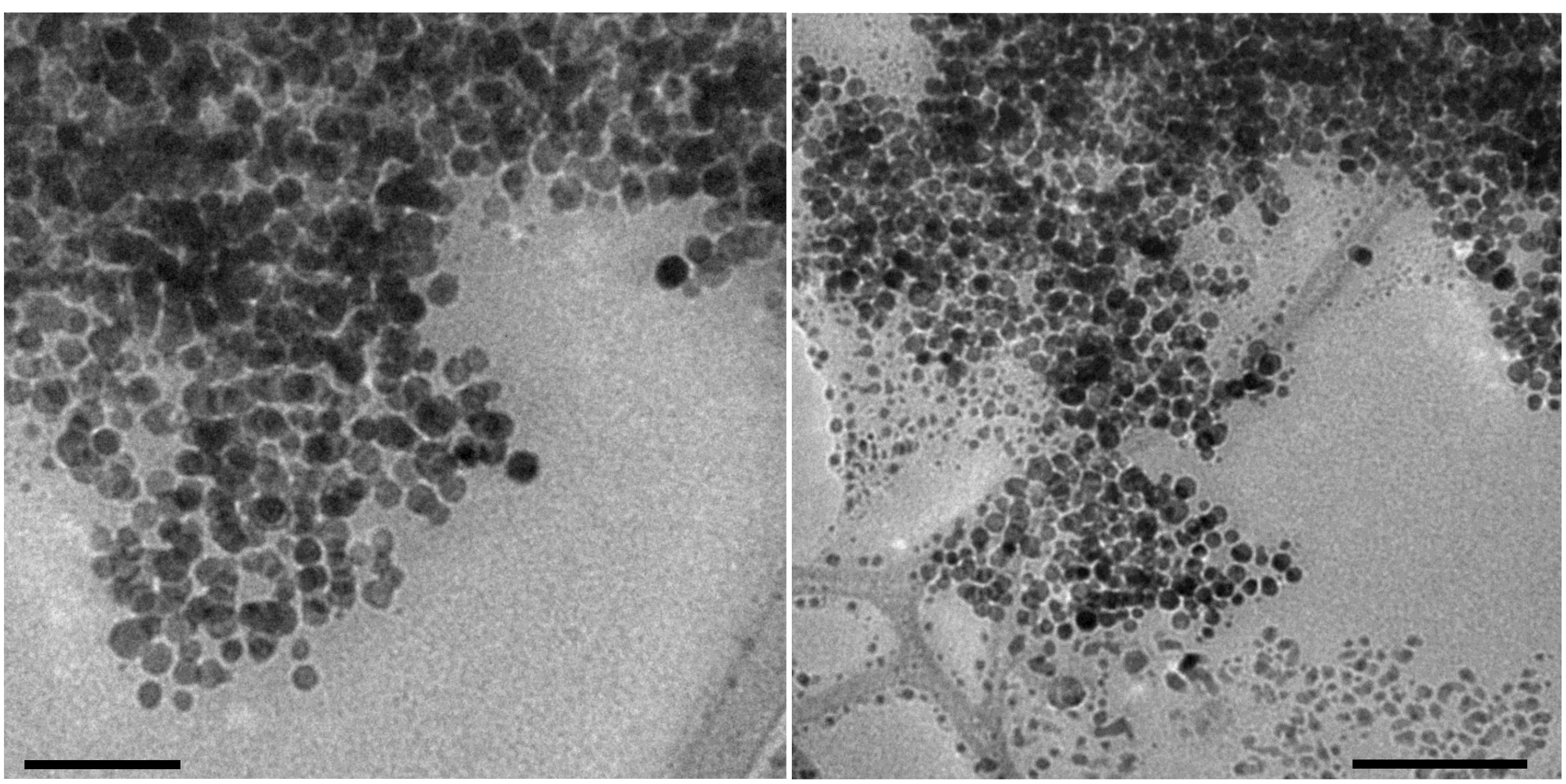

Fig. S30 Image of silver nanoparticles at a scale of 50 nm and 100 nm by TEM

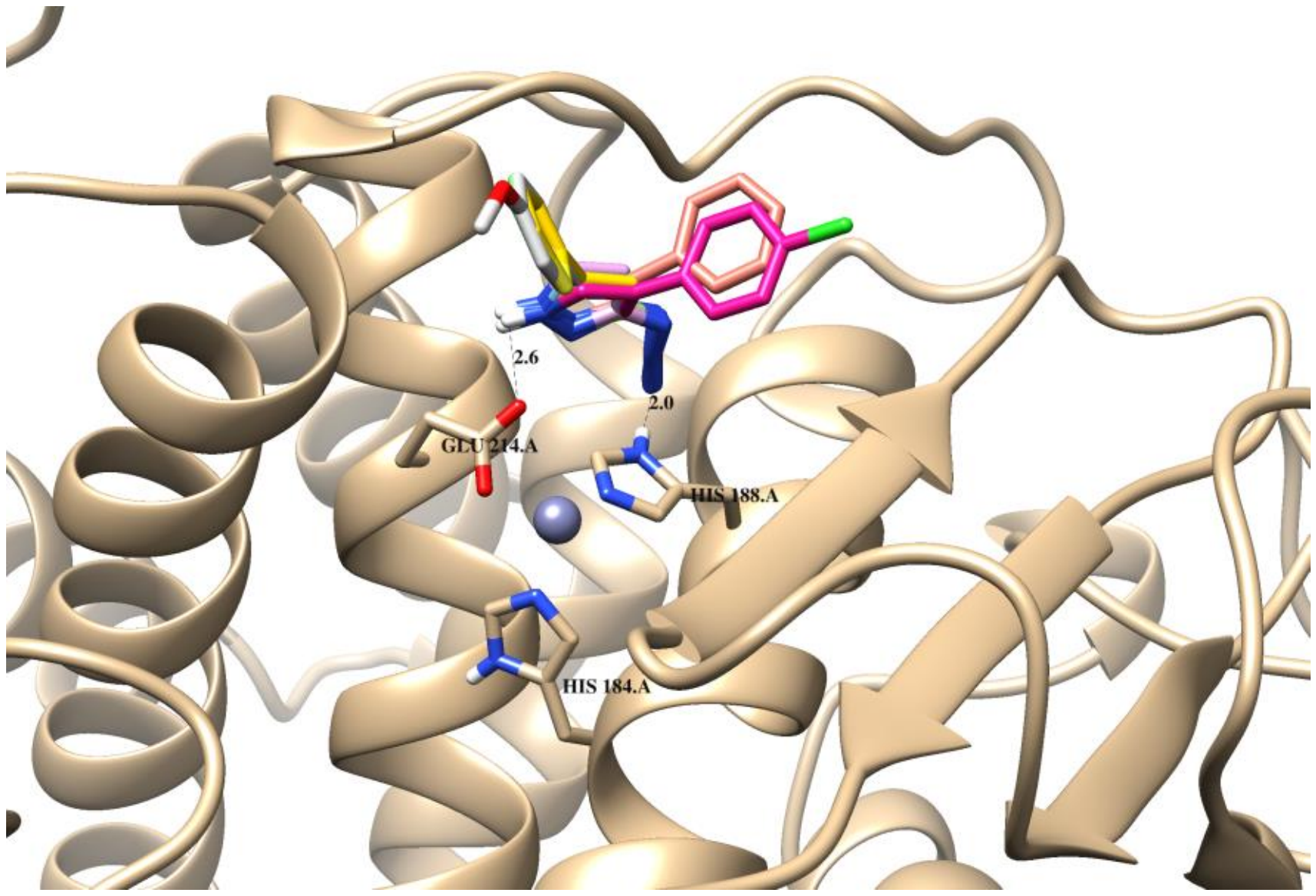

Fig. S31. Binding site interactions of azido-pyrazole compounds: **1** (blue), **2** (pink), **3** (green), **4** (orange), **5** (white), **6** (magenta), and **7** (yellow) within the active site of MEP36. Hydrogen bonds are shown in black, with bond distances indicated in angstroms (Å).

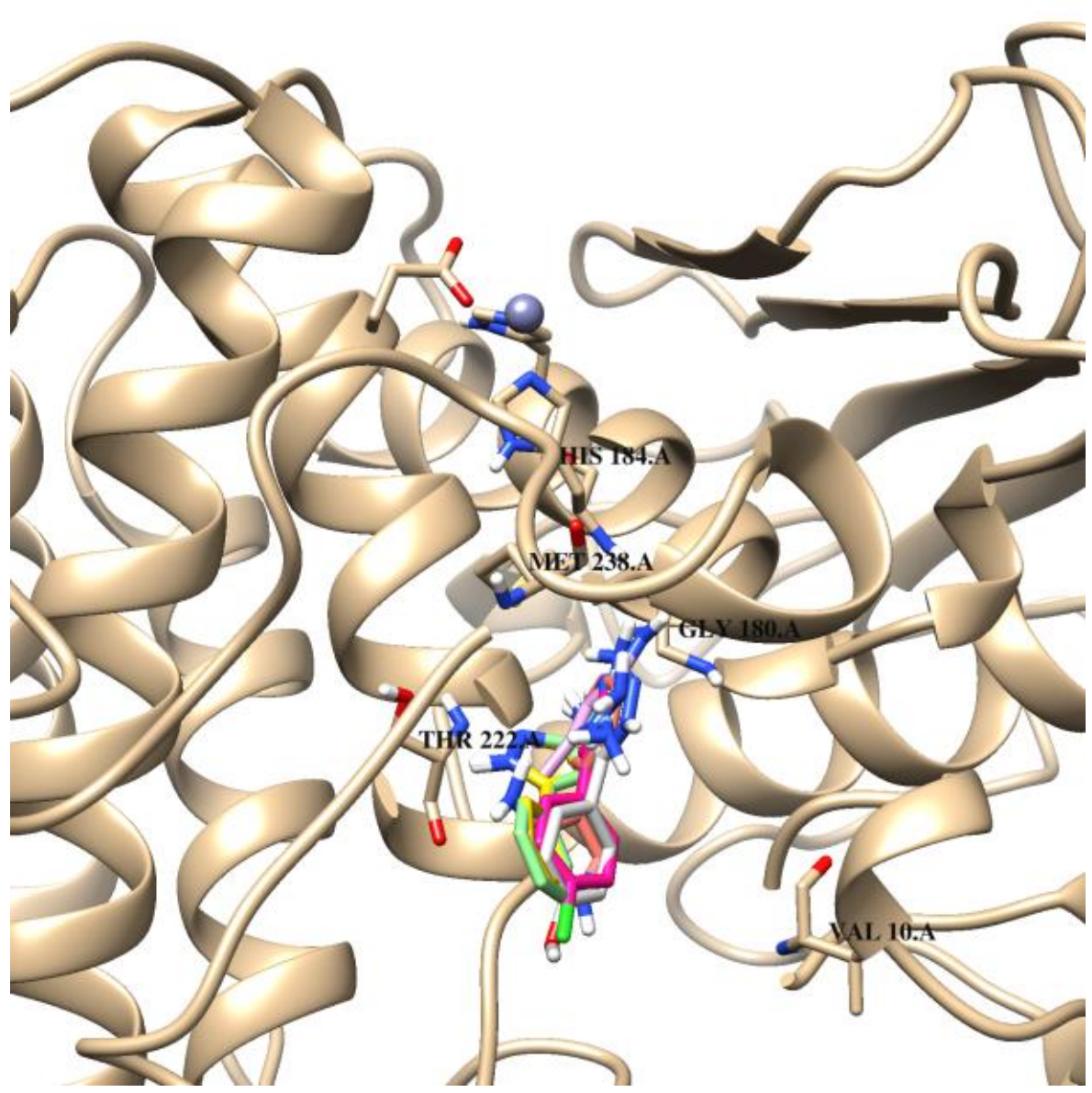

Figure S32. Binding site interactions of the amino-pyrazole precursors **P1**-**P7**: A (blue), B (pink), C (green), D (orange), E (white), F (magenta), and G (yellow).

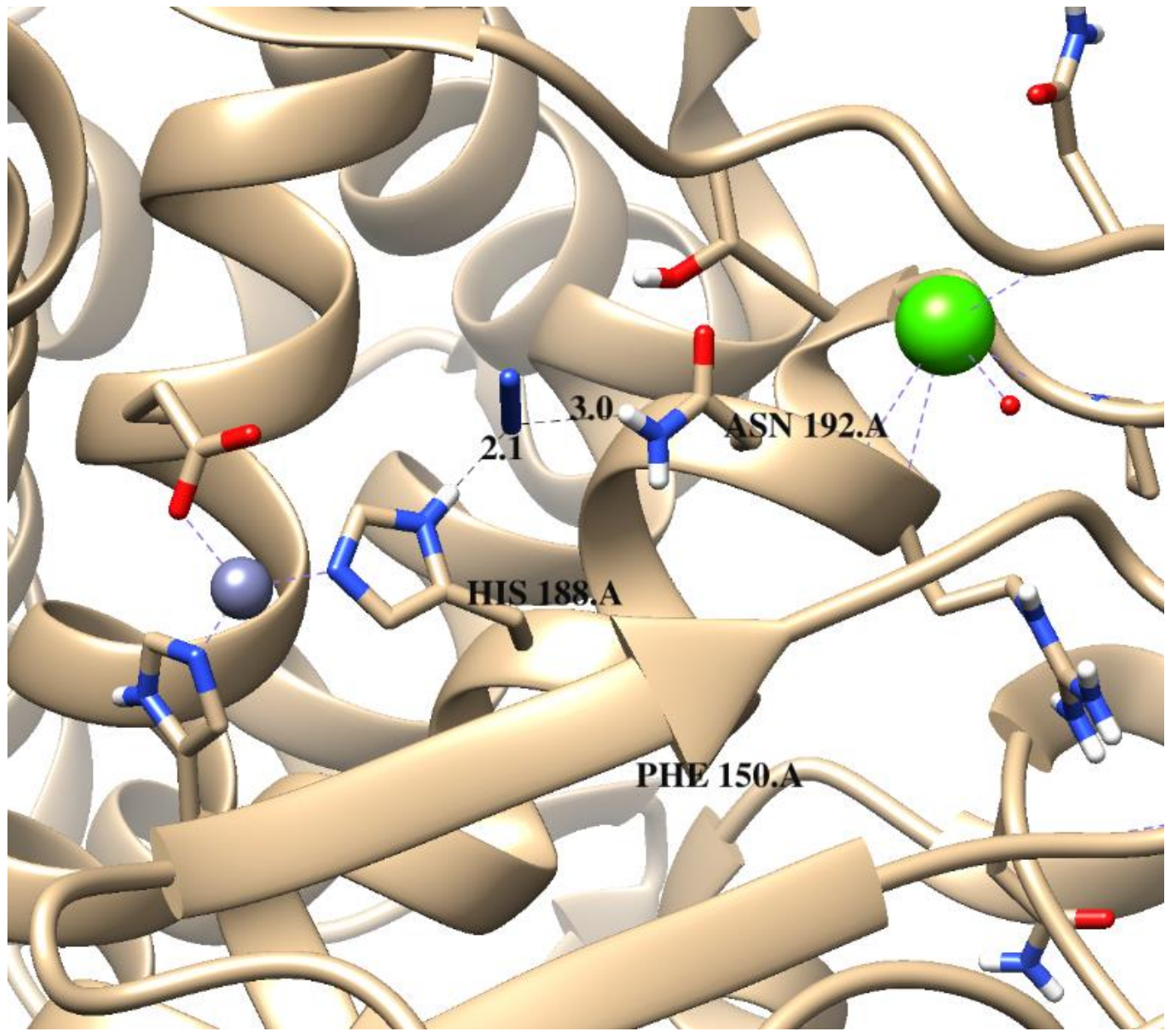

Figure S33. Binding site interactions of the anion azide N_3^-. within the active site of MEP36. Hydrogen bonds are shown in black, with bond distances indicated in angstroms (Å).

Table S1. Asymmetric stretching band of compounds **1-7** and 1-azido adamantane in FT-IR experiments.

**Compound**	A.S. band $-N_3$ group (cm^{-1})
**1**	2111.19
**2**	2116.81
**3**	2118.38
**4**	2119.20
**5**	2122.42
**6**	2131.59
**7**	2113.24
1-Azido Adamantane 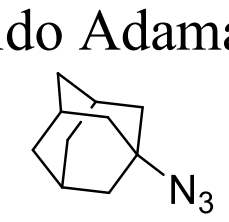	2079.72

EVALUATION OF INHIBITORY TESTS

Table S2. Antifungal activity of azido pyrazole derivatives **1 – 7** and precursors on the radial growth of *Rhizoctonia* sp., *Fusarium oxysporum* and *Alternaria alternata* at 100 µg/mL.

P: Precursor. SD: standard deviation. n = 4. (*) Statistically significative data (ANOVA, $p < 0.05$).

**Compound**	***Rhizoctonia* sp. (% inib. ± SD)**	***Fusarium oxysporum* (% inib. ± SD)**	***Alternaria alternata* (% inib. ± SD)**
**1**	20.13 ± 3.72*	21.01 ± 2.76*	11.01 ± 4.54
**2**	30.81 ± 2.97*	1.88 ± 4.92	0.16 ± 6.40
**3**	100.00 ± 0.00*	100.00 ± 0.00*	95.07 ± 9.12*
**4**	100.00 ± 0.00*	64.81 ± 1.71*	47.26 ± 3.89*
**5**	34.93 ± 4.70*	21.06 ± 2.96*	14.33 ± 3.04*
**6**	100.00 ± 0.00*	61.57 ± 2.15*	54.27 ± 5.01*
**7**	100.00 ± 0.00*	100.00 ± 0.00*	100.00 ± 0.00*
**3P**	18.87 ± 3.12*	27.67 ± 9.98*	22.44 ± 4.75*
**7P**	4.78 ± 5.40	19.14 ± 2.87*	7.93 ± 1.72
**Positive control Captan 50®**	100.00 ± 0.00*	100.00 ± 0.00*	100.00 ± 0.00*

Table S3. Concentrations used for the IC_{50} calculation.

Compound	Concentration (µg/mL)	*Rhizoctonia* sp. (% inib. ± SD)	*Fusarium oxysporum* (% inib. ± SD)	*Alternaria alternata* (% inib. ± SD)
	5	10.18 ± 5.27	17.66 ± 5.61	15.54 ± 4.51
	10	23.68 ± 5.25	27.65 ± 3.46	41.04 ± 4.82
	15	36.05 ± 11.31	23.77 ± 7.28	58.57 ± 6.37
**3**	30	67.11 ± 3.55	44.32 ± 4.37	64.14 ± 7.52
	50	100.00 ± 0.00	73.55 ± 3.56	70.79 ± 1.84
	75	100.00 ± 0.00	100.00 ± 0.00	81.00 ± 2.05
	100	100.00 ± 0.00	100.00 ± 0.00	93.43 ± 10.18
	1	35.75 ± 12.68	-0.70 ± 3.25	1.20 ± 4.34
	5	45.85 ± 12.41	12.53 ± 5.65	2.80 ± 3.39
**3 + AgNPs**	10	54.92 ± 11.97	22.35 ± 2.17	12.80 ± 4.10
	15	65.54 ± 4.28	24.83 ± 2.62	24.00 ± 5.06
	25	69.69 ± 6.64	32.71 ± 4.06	40.00 ± 1.48
	5	21.18 ± 8.81	7.81 ± 2.12	13.72 ± 1.81
	10	32.57 ± 0.00	10.71 ± 0.00	18.90 ± 1.13
	15	50.57 ± 4.18	30.36 ± 0.00	31.10 ± 1.72
**7**	25	66.74 ± 0.84	52.23 ± 3.91	51.52 ± 0.86
	50	100.00 ± 0.00	69.64 ± 0.00	100.00 ± 0.00
	75	100.00 ± 0.00	76.34 ± 14.66	100.00 ± 0.00
	100	100.00 ± 0.00	100.00 ± 0.00	100.00 ± 0.00
	1	26.97 ± 5.66	20.22 ± 1.72	45.93 ± 4.34
	5	50.60 ± 11.20	35.92 ± 0.00	49.22 ± 6.41
**Positive control Captan 50**	10	63.01 ± 1.42	44.57 ± 3.60	60.85 ± 4.96
	15	68.97 ± 5.18	42.20 ± 9.92	62.79 ± 4.14
	25	100.00 ± 0.00	100.00 ± 0.00	96.49 ± 6.50
	50	100.00 ± 0.00	100.00 ± 0.00	87.80 ± 8.19

SD: standard deviation. n = 4.

Table S4. *In vitro* IC_{50} values of synthesized compounds **3**, **7** with or without silver nanoparticles

**Compound**	**Microorganism**	**HillSlope**	R^2	**IC_{50} ± EE (µM)**	**95% Confidence interval**
**3**	*Rhizoctonia* sp.	2.236	0.9744	100.66 ± 5.84	14.98 - 23.17 19.32 - 36.40 11.06 - 21.80
	Fusarium oxysporum	1.770	0.9041	143.30 ± 6.16	
	Alternaria alternata	1.060	0.9407	83.91 ± 6.16	
**3 + AgNPs**	*Rhizoctonia* sp.	1.7660	0.9913	52.14 ± 7.46	0.1517- 614.200
**7**	*Rhizoctonia* sp.	1.799	0.9654	75.26 ± 5.70	11.37 - 18.21 20.63 - 34.68 16.33 - 27.04
	Fusarium oxysporum	1.635	0.9646	139.90 ± 7.11	
	Alternaria alternata	2.406	0.9621	109.93 ± 7.16	
**Positive control Captan 50**	*Rhizoctonia* sp.	0.9822	0.9522	14.17 ± 4.46	1.863 - 9.747 4.025 - 23.750 0.4655 - 14.270
	Fusarium oxysporum	1.3920	0.9482	32.50 ± 2.86	
	Alternaria alternata	0.5526	0.9241	8.55 ± 1.90	

MOLECULAR DOCKING

Table S5. Docking scores obtained with AutoDock Vina, Smina, Qvina2, and Qvinaw for azido-pyrazole compounds against MEP36 (PDB ID: 7QFY), along with simple and extended consensus values (kcal/mol).

Compound	Vina	Smina	Qvina2	Qvinaw	Consensus
**NaN_3**	-3.0	-3.1	-3.0	-3.0	-3.0
**1**	-5.2	-5.3	-5.4	-5.2	-5.3
**2**	-5.6	-5.7	-5.8	-5.6	-5.7
**3**	-6.7	-6.8	-6.9	-6.8	-6.8
**4**	-6.2	-6.3	-6.4	-6.2	-6.3
**5**	-6.6	-6.7	-6.8	-6.6	-6.7
**6**	-6.4	-6.5	-6.6	-6.4	-6.5
**7**	-6.2	-6.3	-6.4	-6.2	-6.3

Table S6. Docking scores obtained with AutoDock Vina, Smina, Qvina2, and Qvinaw for amino-pyrazole precursors against MEP36 (PDB ID: 7QFY), along with simple and extended consensus values (kcal/mol).

Compound	Vina	Smina	Qvina2	Qvinaw	Consensus
**1P**	-4.0	-4.1	-4.2	-4.0	-4.1
**2P**	-4.4	-4.5	-4.6	-4.4	-4.5
**3P**	-6.0	-6.1	-6.2	-6.0	-6.1
**4P**	-5.9	-6.0	-6.1	-6.0	-6.0
**5P**	-6.3	-6.4	-6.5	-6.4	-6.4
**6P**	-5.9	-6.0	-6.1	-6.0	-6.0
**7P**	-5.7	-5.8	-5.9	-5.8	-5.8
